# Woodward–Hoffmann’s *Stereochemistry of Electrocyclic Reactions*: From Day 1 to the *JACS* Receipt Date (May 5, 1964 to November 30, 1964)

**DOI:** 10.1021/acs.joc.5b01792

**Published:** 2015-10-27

**Authors:** Jeffrey I. Seeman

**Affiliations:** Department of Chemistry, University of Richmond, Richmond, Virginia 23173, United States

## Abstract

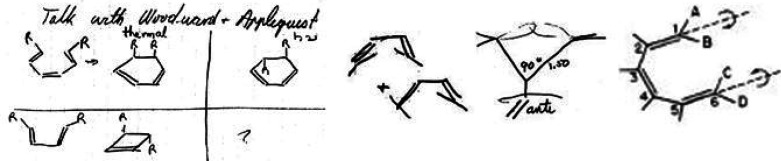

The publication in January 1965 of the first Woodward–Hoffmann paper, *The Stereochemistry of Electrocyclic Reactions*, ushered into organic chemistry both an explanation of the stereochemistry and “allowedness” or “forbiddenness” of concerted reactions and an impetus for untold numbers of research projects. In the current paper, details of the collaboration between R. B. Woodward and R. Hoffmann, from when they first met to discuss the solution to the “no-mechanism problem” to the date their first paper was received in the offices of the *Journal of the American Chemical Society*, will be discussed and analyzed. The primary focus will be on the historically relevant extant documents from the early 1960s. These include Hoffmann’s laboratory notebooks describing his research, including his extended Hückel calculations used to explain and predict the stereochemistry of electrocyclic reactions. Drafts of the *Stereochemistry of Electrocyclic Reactions* paper and letters and notes by Woodward, Jerome Berson, and others will further illuminate the development of this first Woodward–Hoffmann paper.

## I Introduction

**Figure 1:**
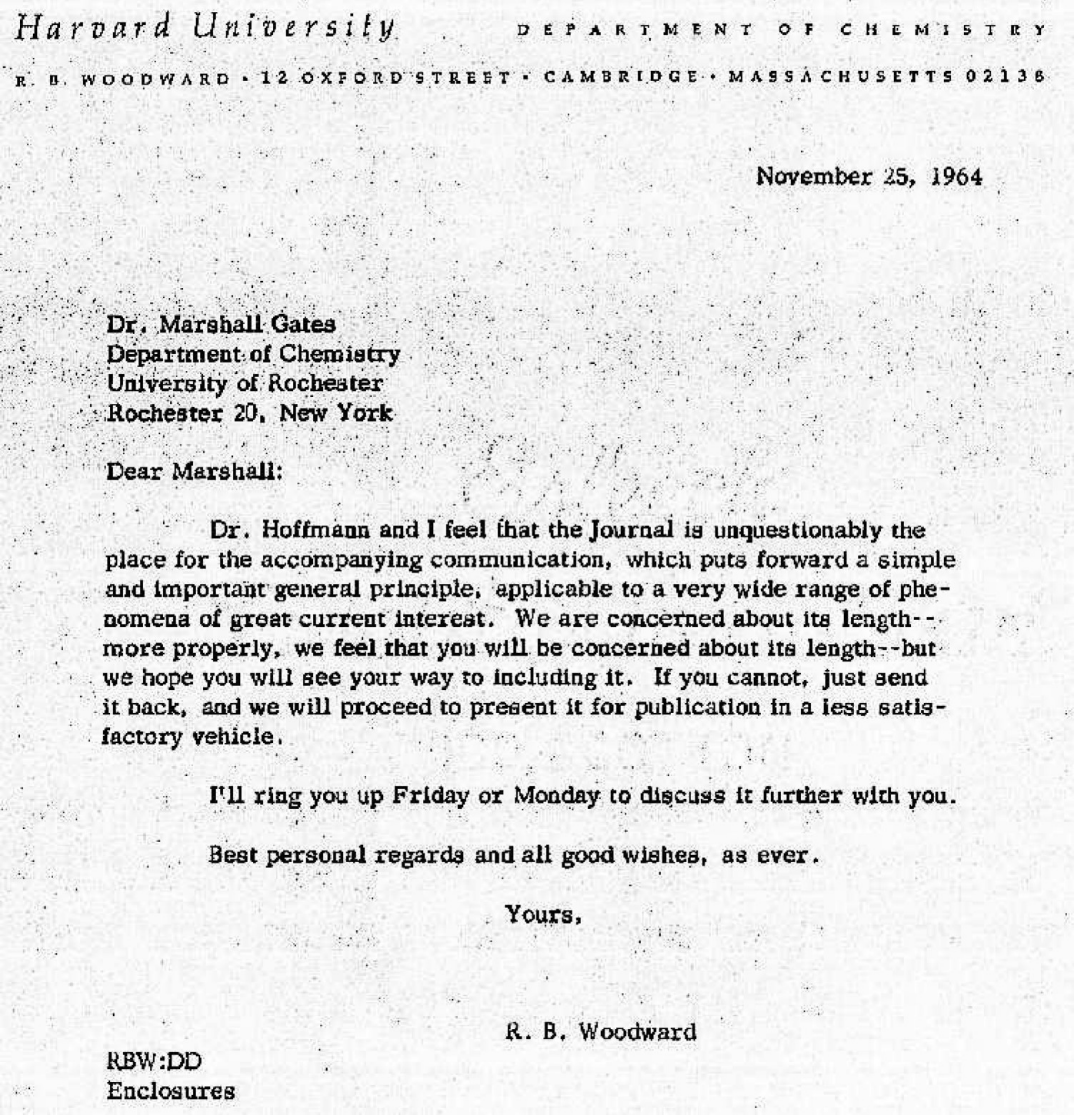
Cover letter^2^ written on November 25, 1964 by R. B. Woodward that accompanied the submission of *Stereochemistry of Electrocyclic Reactions*.^1^

The day before Thanksgiving, 1964. November 25, 1964, to be more precise. On that day, R. B. Woodward submitted the first Woodward–Hoffmann (W–H) paper, *Stereochemistry of Electrocyclic Reactions*,^1^ to the *Journal of the American Chemical Society* (*JACS*) along with the cover letter which said in part (Figure 1),

“We are concerned about its length—more properly, we feel that you will be concerned about its length—but we hope you will see your way to including it. If you cannot, just send it back, and we will proceed to present it for publication in a less satisfactory vehicle.”^2^

**Figure 2:**
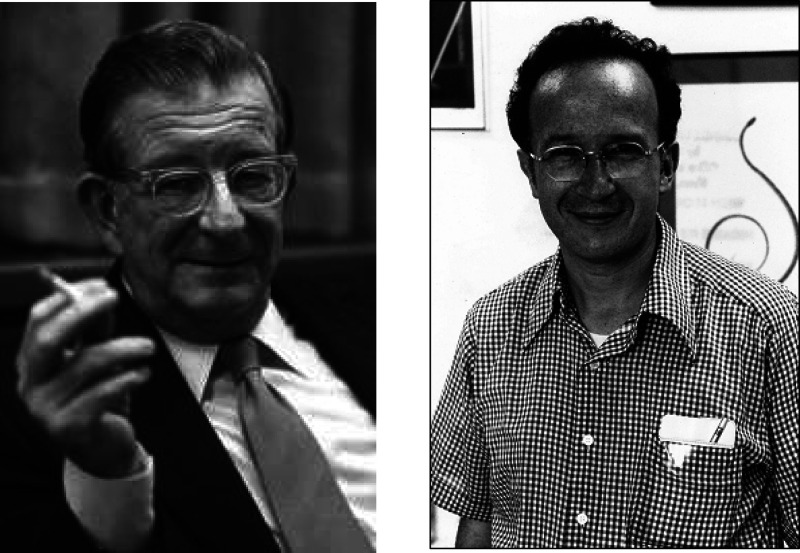
(Left) R. B. Woodward with his ever-present cigarette, mid-1960s. (Right) Roald Hoffmann, mid-1960s. “Note the snake in the background. There’s a story there: the plates are from first real scientific study of venomous snakes, published in 1796, in Patrick Russell’s *An Account of Indian Serpents*.^6^”^7^ “The plastic insert in my shirt is more to the point of the paper than the snake—it is part of the classic nerd appearance of the day. And Woodward would not be caught dead wearing one.”^8^

**Figure 3:**
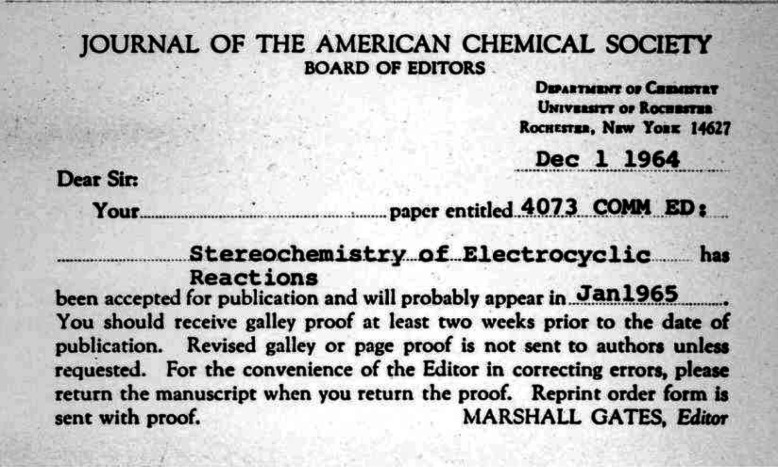
Manuscript acceptance postcard^4^ sent by Marshall Gates to R. B. Woodward on December 1, 1964 for the first paper in the Woodward–Hoffmann series, *Stereochemistry of Electrocyclic Reactions*.^1^

Given that Woodward and his coauthor Roald Hoffmann (Figure 2) had just invented the term “electrocyclic reaction”, the submission’s title hardly garnered the immediate drama or portraiture that many of Woodward’s previous papers had achieved. Consider that Woodward’s *The Total Synthesis of Strychnine* began with the one word sentence “STRYCHNINE!”^3^ However, *Stereochemistry of Electrocyclic Reactions*, received on November 30, 1964 and accepted for publication within 24 h (Figure 3),^4^ would propel even further the legacy of Woodward. For Hoffmann, it would transform his reputation from a budding chemical physicist—he had, in 1962, received several academic job offers for what appeared to be his promising future in that discipline—to, as he described in 1996, “an explainer, the builder of simple molecular orbital models”.^5^

These papers would secure name recognition for both Woodward and Hoffmann by generations of chemists—and students of organic chemistry—for perhaps as long as the field survives. For it was the explanations provided in that paper and subsequent ones by Woodward and Hoffmann that explained the reactivity and stereochemistry of all concerted organic reactions–previously described, somewhat but not entirely in jest, as “no-mechanism reactions”^9,10^ by Woodward’s close friend, former postdoctoral associate,^11−13^ and colleague, William von Eggers Doering. Some have asserted that this was Woodward’s, and thus presumably Hoffmann’s, “most significant addition to chemistry”.^14^ In 1981, Hoffmann received the Nobel Prize in Chemistry for this breakthrough discovery along with Kenichi Fukui. Woodward died in 1979; otherwise, he certainly would have received his second Nobel Prize, the first having been received in 1965 for his total syntheses.

This paper will trace the research underlying Woodward and Hoffmann’s first paper^1^ on the *Conservation of Orbital Symmetry*, more typically referred to as the Woodward–Hoffmann rules (W–H rules). Attention will be placed on extended Hückel (EH) calculations, including those not related to orbital symmetry, during the seven months from May 5, 1964, the date their collaboration began, to November 30, 1964 (referred to herein as “the time period”), the date the electrocyclization paper^1^ was received by *JACS.* Hoffmann and the author have conducted 5 days of interviews, inspecting and discussing every page of his laboratory notebooks from 1964 and 1965 and many pages before and after those dates. It provided a rare but not unique^15^ opportunity to follow essentially the day-by-day research activities that led directly to a Nobel Prize.

The drafts of this first W–H paper will also be analyzed in terms of authorship and timing. The progress of Hoffmann’s extended Hückel calculations relative to Hoffmann’s other research and to the writing of the paper will speak also to Woodward’s and Hoffmann’s perception of the importance of this work. Were Woodward and Hoffmann sensitive to competition and rushing to publish the solution to the well-recognized no-mechanism quandary? In a sense, we shall see first hand “the story behind the story”.

Of particular focus will be the nature of the collaboration between Woodward and Hoffmann. When their collaborations began, Woodward, at 47, was a year from receiving his Nobel Prize and was the world-acclaimed master of natural products structure determination and total synthesis. Hoffmann, at 26, was less than two years beyond the receipt of his Ph.D. and was a chemical physicist who was just learning organic chemistry. This asymmetrical relationship^16,17^—the vaunted position of Woodward and the relatively unknown (within the organic chemistry community) but blossoming Hoffmann—is worthy of note as it relates to their evolving relationship.

Furthermore, the Woodward–Hoffmann collaboration was one of the very first interdisciplinary collaborations between an organic chemist and a theoretical–computational chemist that had lasting consequences on organic chemistry. For an earlier though less consequential example, see the work of Massimo Simonetta^18^ and Saul Winstein^19^ on the use of the LCAO (linear combination of atomic orbitals) semiempirical molecular orbital method on neighboring group participation published in 1954.^20^ The Woodward–Hoffmann collaboration was notable for a number of reasons: First, for its explicative and predictive qualities. Second, because the collaboration evolved and expanded over many years, both for the participants and the breadth of its science. And last, for the unequivocal demonstration that molecular orbital (MO) theory had not just supplanted valence bond theory (VB) in organic research but that MO theory must now be within the vocabulary of practicing organic chemists. (With the recent work of Shaik and Hiberty,^21−23^ VB theory has made a resurgence in state-of-the-art organic chemical research.)

## II Preliminary Thoughts

With this special issue, *The Journal of Organic Chemistry* honors the 50th anniversary of the publication of the Woodward–Hoffmann rules—*The Conservation of Orbital Symmetry*. This is most fitting, given that *JOC* is the most eminent publication devoted exclusively to organic chemistry and that the Woodward–Hoffmann rules are a major scientific accomplishment in the field.

It is also fitting that this paper is a study of the history of the development of the Woodward–Hoffmann rules. And *JOC* does publish history too! The introductions of many *JOC* papers are history oriented as are most Perspectives. But this paper goes beyond the history of the chemistry. It is a story as to how the chemistry was done, by people acting the way people do—with great diversity of behavior and personality.

I ask each reader to think for a few moments before continuing to read this paper. Please ask yourself,

• What do you imagine that Woodward and Hoffmann each experienced during the first phase of their collaboration, from the day they began working together to the day of the acceptance of their first paper?
• What do you imagine was the nature and character of their collaboration?
• What do you imagine was the extent that outside factors, such as the fear that someone else would publish first or Hoffmann’s need to secure an academic position, influenced Woodward and Hoffmann’s behaviors and the timing of the first Woodward–Hoffmann paper?

With your speculations in mind, based surely on your own experiences as research chemists, you are now invited to the story of Woodward and Hoffmann.

## III The Beginning of the Woodward–Hoffmann Collaboration. “*The Woodward Challenge”*

Roald Hoffmann received his Ph.D. in 1962 from Harvard University under the joint supervision of two eminent physical chemists, Martin Gouterman and William Lipscomb, Jr. Hoffmann’s major area of research as a graduate student was the structure and properties of polyhedral molecules, mainly of boron, using theoretical and computational models. In 1962–1963, together with Lipscomb and fellow student Lawrence Lohr, Hoffmann developed what he later termed the extended Hückel theory (EHT or EH).^5,24^ EHT is a semiempirical quantum chemistry method which calculates the total energies, bond orders (more precisely, bond indices of the Mulliken overlap population type^25−27^), and other physical properties of molecules by considering both π orbitals and σ orbitals, thereby extending the Hückel method into three-dimensional systems. With Gouterman and Lipscomb and extended Hückel theory—though it was not termed that yet—Hoffmann published a number of papers on polyhedral compounds of boron in 1962 and 1963.^26−29^ Lipscomb’s role in the development of EHT is clearly defined in the literature.^24^ As described by Hoffmann,

“LCAO is the more general name for wave functions for a molecule approximated by linear combinations of atomic orbitals. The Hückel method/model is an LCAO method. In what we did with boron hydrides, we expanded the set of functions to include 2s and all three 2ps on every boron atom, and 1s on hydrogen. It was an approximate LCAO procedure. We didn’t know what else to call it. As I turned the method to organics, I made some changes in approximation (but changes minor overall relative to what we did for boranes). And I came up with a name for the approach, not too pretentious. I wanted to make a connection to the Hückel model, so it became “extended Hückel”.”^30^

In spite of receiving a number of academic job offers in 1962, Hoffmann accepted a prestigious three-year Junior Fellowship of The Society of Fellows at Harvard with the intent of taking an academic position in 1965. Woodward, himself, had been a Junior Fellow 25 years earlier (1938–1940). According to the Society of Fellows’ website,

“The purpose of the Society is to give men and women at an early stage of their scholarly careers an opportunity to pursue their studies in any department of the University, free from formal requirements. . . Junior Fellows are selected for their resourcefulness, initiative, and intellectual curiosity, and because their work holds exceptional promise. They are free to devote their entire time to productive scholarship. They may undertake sustained projects of research or other original work, or they may devote their time to the acquisition of accessory disciplines, so as to prepare themselves for the investigation of problems lying between conventional fields.”^31^

This was only a few years following the discovery of conformational analysis by Derek H. R. Barton.^32,33^ Hoffmann’s vision was to understand—and calculate—many of the fundamental properties of organic molecules. At that time, EHT was a unique tool of which, for some short time, he was the sole practitioner. Hoffmann was to immediately put it to grand use, calculating the energies and properties of organic compounds as a function of their three-dimensional structures. Today, Hoffmann characterizes EHT as follows:

“Folks would eventually run from the method, but I accepted its deficiencies because EH got the trends right. It quickly proved to be lousy theoretical chemistry but it made and makes connections with reality. And, it is transparent; one can easily see why the numbers come out as they do. I kept on using it for decades, not because I’m stubborn but because I wanted to do something with real molecules. And I had a feeling that EH could do it. It gets the basic electronics right.”^34^

Indeed, in 2008, Ken Houk, a leader in computational chemistry, summarized the value of these early theoretical models as follows:

“Hückel theory and extended Hückel theory are wonderful examples of theories of low accuracy and precision providing excellent explanations and guides to experiment.”^35^

At the start of his Junior Fellowship, Hoffmann changed course, from complex boron molecules to fundamental organic chemistry, applying EHT to almost everything in sight. Almost immediately, he published on simple hydrocarbons and alkanes,^36,37^ then carbonium ions^38,39^ and azines,^40^ in 1963 and 1964, all using EHT. More complex organic chemistry was soon to follow, with a leap from simply going through the dictionary of organic molecules, starting on page 1 to solving a major problem in the field.

From 1962 to July 1965, Hoffmann’s office was three doors down the corridor from E. J. Corey’s office in the basement of Mallinckrodt Hall. Corey, only nine years senior to Hoffmann, had become a full professor at Illinois in 1956 at age 27 and had moved to Harvard in 1959. In fact, Hoffmann had arrived at Harvard *before* Corey though as a graduate student, not a tenured full professor. Hoffmann and Corey both recall the younger chemist’s frequent visits to the elder’s office to discuss and soak in the finer points of organic chemistry. They both have used the word “tutor” to describe the Corey’s relationship to Hoffmann.^41^ Indeed, Hoffmann acknowledged Corey in several of his early publications^37−39^ and in one manuscript submitted in 1964 that was not published.^42^ Corey used some of Hoffmann’s EHT calculations in a 1964 publication acknowledging Hoffmann’s assistance.^43^ Hoffmann had another source of organic chemical education: he was sitting in on a course in small ring chemistry taught by Douglas Applequist, on leave from the University of Illinois in the spring of 1964 and substituting for Corey who was on sabbatical leave.

Hoffmann and EHT were primed, ready to respond to the most serious scientific challenges that chemistry could offer. And who should appear in Hoffmann’s life but R. B. Woodward.

Little need be said in this paper of Robert Burns Woodward. In 1964, he was at the height of his powers. Much has been written about him,^44^ and more will surely follow. His reputation as the greatest synthetic chemist can be matched by claims that he was also the greatest at natural products structure determination of his time, fields that he “dominated so decisively in [his] era”.^45^ Recently, it was claimed that he was one of the greatest physical organic chemists too.^46^ In the 1950s and 1960s and into the 1970s, Woodward was the Pope of Organic Chemistry^47,48^—there was no No. 2. But perhaps in time, there would be a rival:^49^ the man three doors down the corridor from Hoffmann, E. J. Corey.

**Figure 4:**
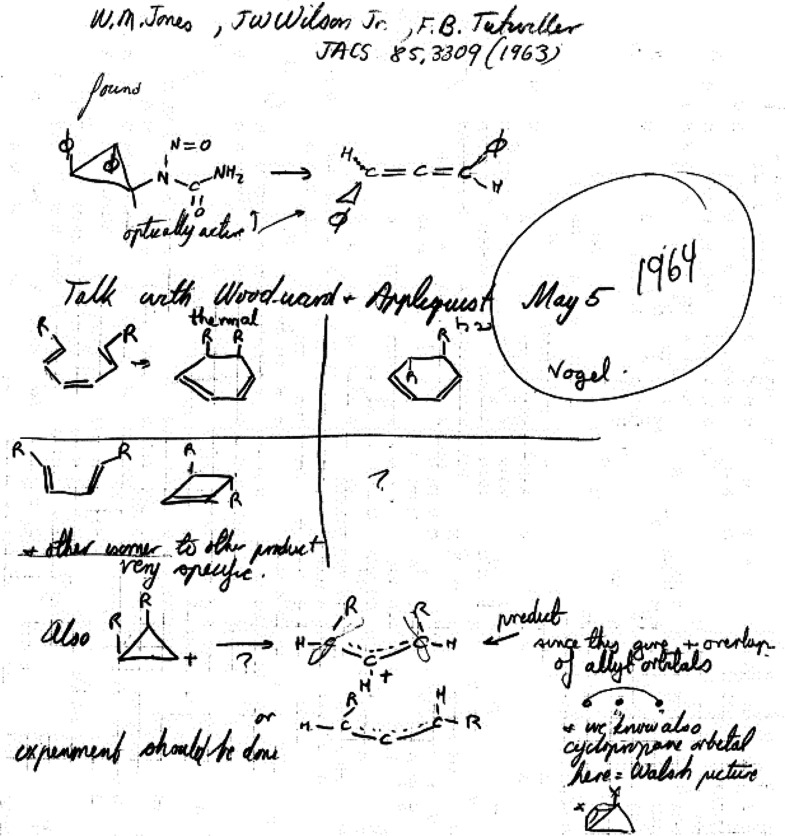
Page 80 of Roald Hoffmann’s 1964 laboratory notebook “Early 1964”^50^ dated “May 5” (the “1964” was added by Hoffmann ca. 2004) and reporting “Talk with Woodward & Applequist.” This page represents *The Woodward Challenge*—a term coined herein—as well as the beginning of the collaboration of Hoffmann with R. B. Woodward which extended far beyond the chemistry depicted in this figure.

The collaboration between Hoffmann and Woodward on what was to become the “Woodward–Hoffmann rules” and *Conservation of Orbital Symmetry* presumably began on May 5, 1964, and is recorded in Hoffmann’s laboratory notebook (Figure 4). Hoffmann kept at the time a record of his work in the classic Boorum & Pease bound notebooks (Standard Figuring Book, No. 1602 1/2, a fact pointed out to this author with emphasis by Hoffmann^7^), a habit he developed in the Lipscomb group. In Hoffmann’s handwriting on page 80 of his *Early 1964* laboratory notebook is “Talk with Woodward & Applequist”^50^ (Figure 4). Page 80 also memorializes what was presumably Woodward’s presentation to Hoffmann of the no-mechanism conundrum, herein referred to as *The Woodward Challenge*.

**Figure 5:**
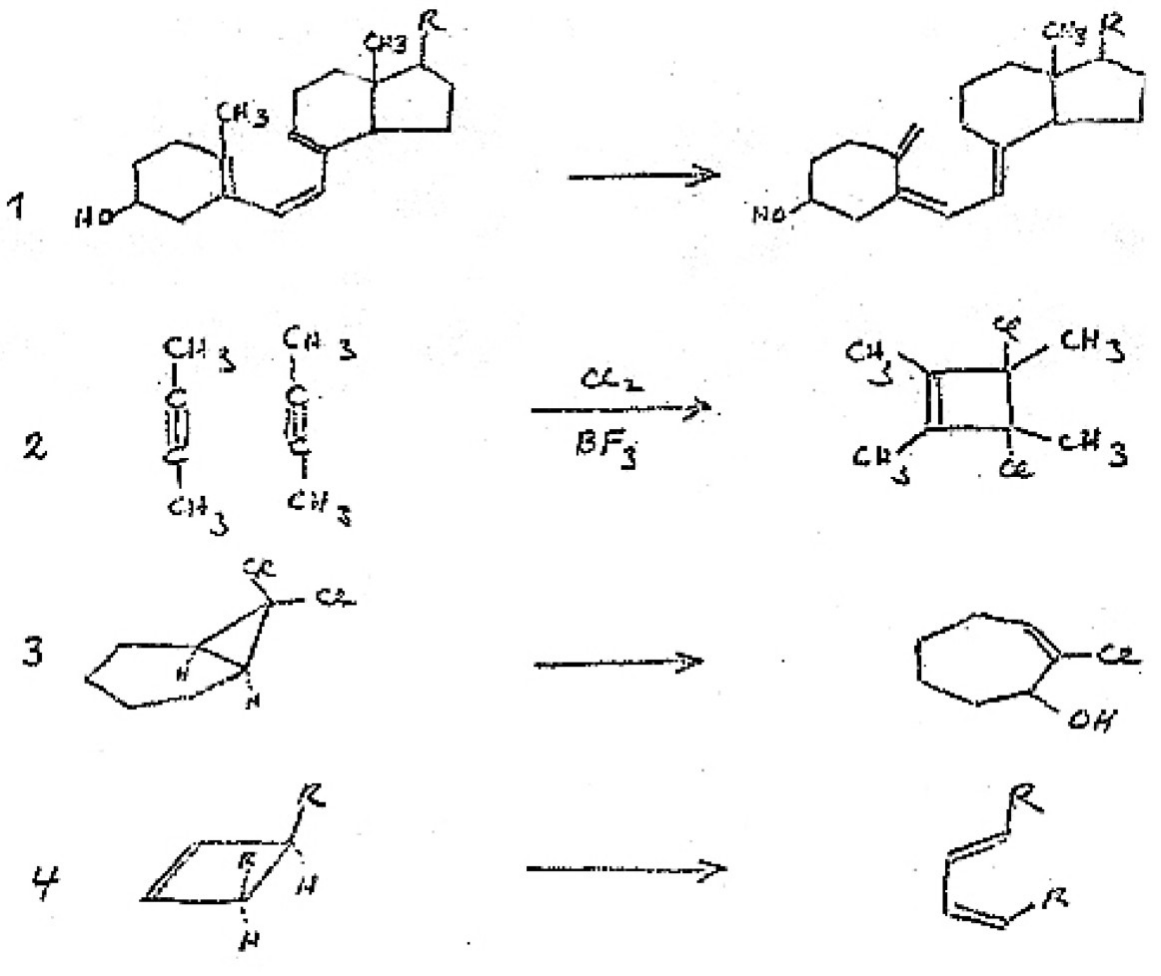
Handwritten slide^52^ by R. B. Woodward from the manuscript of his 1973 Cope Award address^51^ depicting Woodward’s “four mysterious reactions”. Compare reactions 3 and 4 in this figure with the reactions drawn in Figure 4.

**Figure 6:**
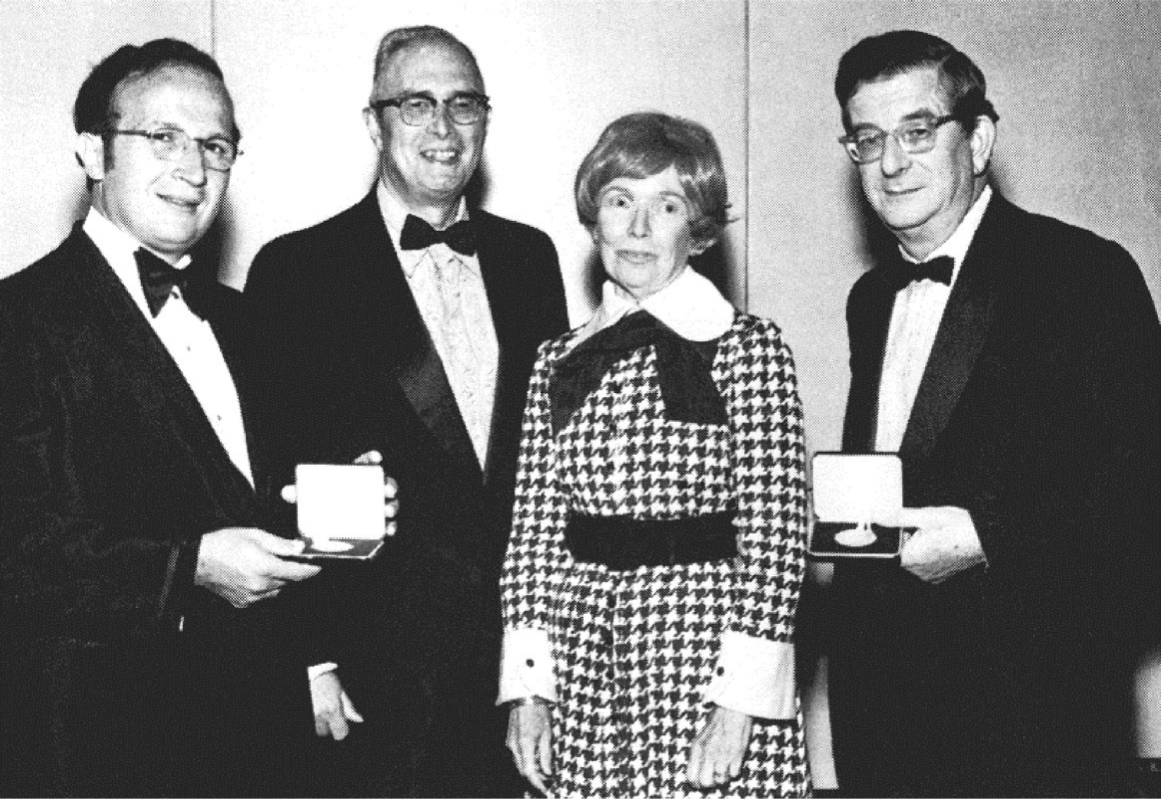
First Cope Awardees, Roald Hoffmann (far left) and R. B. Woodward (far right), with Mrs. Arthur C. (Harriet) Cope and Herman Bloch, then chairman of the board of the American Chemical Society, 1973. Photograph courtesy of Harvard University Archives.

*The Woodward Challenge* began with the mysterious alternating stereochemistry for four-electron and six-electron electrocyclizations *and* for alternating stereochemistry in the thermal and photochemical electrocyclizations of the same number of electron-systems. Also shown on page 80 as part of *The Woodward Challenge* is the two electron electrocyclic ring opening of cyclopropyl-X to an allyl cation. These were two of the community’s—and especially Woodward’s—most glaring examples of the inexplicable no-mechanism problem. This is illustrated by the slide drawn by Woodward for his 1973 Cope Award address (Figure 5), an award he shared jointly with Hoffmann (Figure 6). Figure 5 shows what Woodward characterized in his Cope address as the “four mysterious reactions”^51^ that burdened, even plagued him, and eventually propelled him to his frontier orbital solution and collaboration with Hoffmann.

It is also important to note that the simple orbital explanation (what we would call a Frontier Orbital argument) does not appear as such in the Woodward Challenge for the 1,3-butadiene or 1,3,5-hexatriene reactions. But it is there, in Hoffmann’s handwriting, in the pictography of the allyl cation HOMO for the allyl to cyclopropyl cation reaction. However, as noted below, Hoffmann did not incorporate the Frontier Orbital concept in his thinking until late in his work on the electrocyclic reactions. This was one of *Woodward’s* contributions to the first paper.

As Hoffmann relates

“At that point, no calculations other than extended Hückel could have helped him because both σ and π electrons are involved in all of these reactions. A year or two later, a number of methods such as CNDO and INDO could have done the same thing. *Ab initio* calculations could just barely approach problems of this size at that time.”^53^

The curious among us—and who among us is not curious?—would like to know more of that first meeting between Woodward and Hoffmann. What exactly did Woodward say? Where was the meeting? Was Applequist present and what were his contributions, if any? Was this to be a collaboration or simply an exchange of information? Or all of the above? And if this were to be a collaboration, what were the arrangements, if any? Did Woodward explain how important the project was to him?

Unfortunately, Hoffmann does not remember anything that was discussed at that meeting with Woodward. In fact, Hoffmann does not even remember having such a meeting with Woodward, let alone a meeting with Woodward and Applequist! Nor that such a meeting did *not* take place.^41^ Nor does Applequist remember such a meeting.

To complicate the history of the initiation of the Woodward–Hoffmann collaboration, Hoffmann has informed this author that Corey has challenged May 5th as the date of Hoffmann’s meeting with Woodward, as recorded on page 80 of his (Hoffmann’s) notebook (Figure 4). According to Hoffmann, Corey said that that date is “fictitious” and “a clumsy attempt to mislead”.^41^ Regrettably, Corey has not revealed to Hoffmann nor to this author, after being specifically asked, the basis of his unambiguous, controversial, and if valid, historically important assertion. Nor is Corey willing to discuss this—or anything dealing with the development of the Woodward–Hoffmann rules, including his own claims (Corey’s claims^54,55^ of plagiarism against Woodward and Hoffmann)—with this author. Nor apparently will Corey discuss these matters with anyone else.^56^

To further add to the uncertainty of the initiation of the Woodward and Hoffmann collaboration, in the early 1980s, Hoffmann twice gave a different picture of his first discussions with Woodward. In his lecture at the Woodward Memorial Symposium held August 1981 at the ACS National Meeting^57^ and in his 1983 interview with Andrew Streitwieser, Jr.,^58^ Hoffmann described hearing of the no-mechanism problem in a private conversation with Applequist in April 1964 and meeting with Woodward some time later after he, Hoffmann, had completed several consequential calculations. “I have a vague memory of Applequist on his own telling me of the problem.”^59^

In principle, Hoffmann could have begun to work on the no-mechanism problem as a consequence of Applequist’s course on small ring compounds and their chemistry at Harvard in the spring of 1964. Hoffmann began working on this chemistry only *after* May 5, 1964. As shown in Tables 1 and 2, Hoffmann’s research in this area *all is recorded following* page 80 in his *Early 1964* notebook.

Applequist’s lectures occurred prior to April 27, 1964.^60^ In Applequist’s lectures, Hoffmann was exposed to two of the three reaction types described by Woodward in *The Woodward Challenge* (Figure 4) and two of Woodward’s four mysterious reactions (Figure 5). On page 67 of those lecture notes, in a discussion on solvolyses of cycloalkyl-X and related reactions and “simple cyclic cation products”, Hoffmann records the reaction of cyclopropyl carbocation to allyl carbocation and then, in the presence of HOS (a protic solvent), transformation to “CH_2_=CH–OS”. (Presumably, Hoffmann and not Applequist accidentally omitted a methylene group, i.e., the product should have been CH_2_=CH–CH_2_–OS.) On page 87 of Applequist’s lecture notes, Hoffmann records the thermal ring opening of *cis*-1,2,3,4-tetramethylcyclobutene to (*E*,*Z*)-3,4-dimethyl-2,4-hexadiene and cites the 1959 research of Rudolf Criegee and Klaus Noll.^61^ Also on that page is the analogous reaction of the dimethyl ester of *cis*-3,4-cyclobutene dicarboxylic acid. On page 68, Hoffmann writes “coordinated movement...probably stereochem.” On the other hand, Applequist’s examples were separated not just by 20 pages but also by several days or weeks of lectures. They were mixed within hundreds of other chemical reactions and not presented as a specific mechanistic problem to be solved. Furthermore, Applequist did not tie these two reactions—a 2e^–^ electrocyclization and a 4e^–^ electrocyclization—together as did Woodward. For Hoffmann in April 1964 to connect these reactions together prior to speaking with Woodward would have been masterly. That being said, Hoffmann’s almost 100 pages of notes from Applequist’s lectures^62^ are testimony of Hoffmann’s serious involvement and commitment to organic chemistry at that stage of his career.

A reviewer of this paper asked, “Can the argument that it was the May 5 meeting with Woodward and Applequist that first got Hoffmann thinking about the orbital symmetry and energetics of electrocyclic reactions be strengthened by explicitly stating that examination of his notebook entries prior to the record of that meeting shows nothing that can be reasonably interpreted as being related to electrocyclic or other pericyclic reactions?” The answer to this question is in “yes”: There is nothing *prior* to page 80 of the *Early 1964* notebook (Figure 4) that is suggestive of *The Woodward Challenge*, e.g., computations relating to stereospecificity of valence isomerizations or alternating stereochemistries as a function of electron count or thermal versus photochemical reactions.

That Hoffmann was inspired by *The Woodward Challenge* to begin his calculations or by Applequist’s lectures or by a private conversation with Applequist or by another stimulation is not known for certain and will be discussed in more detail elsewhere. What is important is that a meeting-of-the-minds between Hoffmann and Woodward did take place for their collaboration to begin. Which it did. Regarding the date of May 5, 1964 for that beginning, Hoffmann says, “But the record of it is there, in my notes.”^7^ Whether the Woodward–Hoffmann meeting took place on May 5, 1964 or shortly thereafter, or even shortly before, cannot be determined exactly by comparison with other dates in Hoffmann’s laboratory notebook, since Hoffmann dated very few pages in his laboratory notebook (see Table 1) and often omitted dates in his handwritten letters. However, several conclusions are firm. One, that such a meeting did take place on or around May 5, 1964. Two, that it was that meeting with Woodward that initiated Hoffmann’s calculations on the stereochemistry and energetics of what later became known as electrocyclic reactions.

It is also true that Hoffmann was engaged in applying the extended Hückel method to organic chemical problems and was fascinated by the physical and chemical properties of small rings. Of course, at that time, calculations on large molecules was impractical if not impossible. And it was not necessary to, for example, calculate electrocyclizations of 1,3-cyclohexadienes imbedded in a steroid molecule, e.g., in the vitamin D series as studied by Egbert Havinga, William G. Dauben, and others. One could, and Hoffmann did, perform calculations on 1,3-cyclohexadiene itself.

Further discussions regarding credit, plagiarism, and the Woodward–Hoffmann rules will be discussed in subsequent papers by this author. What is certain—whether beginning on May 5, 1964 or shortly before or shortly thereafter—is that a collaboration between Roald Hoffmann and R. B. Woodward did begin and a breakthrough in organic chemistry was to emerge.

## IV An Overview of Hoffmann’s Research: Day by Day (May 5, 1964 to November 30, 1964)

Tables 1 and 2 summarize the work performed by Roald Hoffmann from May 5, 1964, the apparent initiation date of the Woodward–Hoffmann collaboration, to ca. November 24, 1964, within a day or two of the submission of the first W–H paper, *Stereochemistry of Electrocyclic Reactions*,^1^ referred to herein as “Paper 1” or “W–H Paper 1.” In these two tables, Hoffmann’s calculations, notes, and literature searches are listed by notebook page and topic. Unfortunately, few of these pages are dated. In several instances, dates can be inferred based on chemical meetings attended by Hoffmann, by lectures or seminars that Hoffmann attended, and by his summer 1964 vacation in Sweden with his family.

**Table 1:** Summary of Roald Hoffmann’s Laboratory Notebook Pages from Notebook Titled “Early 1964”^a^ from Page 80 (Dated therein “May 5”) to Mid-July 1964^b^

date and other identifying information (1964)	notebook page numbers	orbital symmetry^b^	topics
May 5, 1964	80	OS	meeting with Woodward & Applequist
	81–82	OS	cyclopropyl cation and radical
	83	OS	1,3-butadiene
	84		enone photochemistry
	85–87	OS	1,3-butadiene
	88–89		bridgehead carbocations
	90–91	OS	1,3-butadiene
	92		spiropentane, piperidinium salts
	93		allyl anion
	94	OS	cyclobutene
	95	OS	cyclohexadiene
	95–97		glyoxal
	97		cyclopropyl carboxaldehyde
	98		*n*-octanal
	99		di-*n*-butyl ketone
	100		acetaldehyde
	101		cyclobutanone, cyclopentanone
	102		cyclohexanone
	103–105		cyclic ketones
	106		pentyl ethyl ketone, hexyl ethyl ketone
	107		acetone, cyclopropanone
	108		ketene, benzophenone
	109		acyclic aldehydes and ketones
	110		cyclobutylcarboxaldehyde
	111		methyl cyclopropyl ketone
	112		cyclopropyl carboxylaldehyde
	113	OS	cyclopropyl cation and cycloalkyl carboxylaldehydes
June 9	114–115		alkyl carbocations
	116		methyl ethyl ketone
	117		methyl propyl ketone
	118		2-pentenone
	119		photochemical H-transfer summary
	120		ammonium salts, 2-pentanone
June 17	121		methyl cyclopropyl ketone
	122		cyclopropyl carboxaldehyde
	123		Diels–Alder graphic
	124		cyclic ketones
	125		acyclic ketones
June 24–27^c^	n/a	meeting	Conference on Reaction Mechanisms, Corvallis, Oregon
	126		cyclohexanone, cyclohexadienone
	127–128		2 + 2 photocycloadditions of enones
	129		*cis*- and *trans*-1,3-butadienes
	130–131		allyl anions
	132		cyclopentyl, cyclopentenyl, bicyclo[3.1.0]hexyl, and hydrindenyl cations
	133–134		C_*n*_ cyclic and linear hydrocarbons
	135		1,3-butadiene singly and doubly positive and negative charged ions
	136		cyclic and linear polyenes
	137–138		cumulenes
	139		propenal, *s*-*trans*-1,3-butadiene
	140–141		ethyl carbocation
	142		aromaticity in conjugated monocyclic rings (C_*n*_ = 4–14)
	143		tetrahedrane
	144		methylene (carbene)
	145		acetylene
	146		methylene and ethyl carbene
	147		diazirine
	148		1,3,5-hexatriene but not related to orbital symmetry, allene
	149–150		allene
	151		hyperconjugation
	152		*n*-orbitals of ketones
Last page in laboratory notebook.			

a From Hoffmann, R. *Laboratory Notebook (“Early 1964″)*, Cambridge, MA, 1964.

b The third column indicates whether the material on the given line was related to the first Woodward–Hoffmann paper on electrocyclizations. OS = related to orbital symmetry but, more specifically, to electrocyclic reactions. In this time period, Hoffmann had interests in what later was termed cycloadditions and sigmatropic rearrangements but only in 1965 did he begin to perform calculations related simultaneously to orbital symmetry and these other reaction types.

c The placement of this row within the table is approximate, i.e., it could be one or several rows higher or lower, as very few pages were dated but the dates of the conference are known exactly.

**Table 2:** Summary of Roald Hoffmann’s Pages from Notebook Titled “Summer → November 1964”^a^ from Approximately Mid-July 1964 through November 1964

date and other identifying information	notebook page numbers	orbital Symmetry^b^	topics
July 20–24, 1964	n/a	meeting	International Symposium on Organic Photochemistry, Strasbourg, France
July or August to late September	1–45		literature searches and journal reading notes, including these topics: acetylenes, aldehydes, benzocycloheptenes, 1,3-butadienes, carbonium ions, cumulenes, cyclobutanedione, cyclopentadiene, cyclopropene, diazirine, enone photochemistry, fluorinated biphenyls, ketene, norbornyl compounds, radicals, and sulfur hexafluoride
September 24–25	36–38		hypothesis of bond cleavage and bond strengths in photochemical reactions. N_2_O^+^, AsO, C_2_, Cl_2_CS, CS_2_^+^, etc.
late September			return to Harvard
	46		cyclopropene
	47		tetrahedrane, diazomethane
	48		cumulenes
	49		literature
	50–51		C_1_H_*n*_ (neutral, cation)
October 6	52–54		Paul Bartlett lecture: cycloadditions of halogenated olefins with 1,3-butadiene
	54–55		2 + 2 versus 4 + 2 reactions and bond angle considerations, neither calculations nor frontier orbital concepts discussed
	56–63		diazirines, diazomethane, cyclopropenes
	64		cyclopropene and cyclobutene
	65		cumulenes
	66–67		hypothetical molecules and reactions, isodiazomethane
	68–69		diazirines, diazomethane
	70		CO_2_ and N_2_O
	71		1,3-butadiene geometrical distortions, diaziridine
	72		difluorodiazirine
October 13–14		meeting	Natick Conference: Lectures by R. Criegee and E. Vogel on valence isomerizations and by C. DePuy on cyclopropyl-X solvolyses
	73		pyrazine, pyridazine, pyrimidine
October 19	74–75	lecture	Rolf Huisgen lecture, on valence isomerizations of cyclooctatetraene, cyclooctatriene and benzocyclobutene
October 22	76		Victor Laurie lecture
	77		cyclopropene
	78		diazarines, pyradazine
	79–81	OS	*cis*-2,3-dimethylcyclopropyl carbocation
	82		diazirine, diazomethane
	83		toluene, carbocations
November 3	84		3-vinylcyclopropene and 3-cyclopropenyl carboxaldehyde
	85–86		deuterium isotope effect in cyclohexane
	86		deuterium isotope effect in ethane
	87		acetylene and pyrylium salts
	88		cyclopropenyl carboxaldehyde
	89–91		bridged annulenes, norcaradines
	92		cyclopropenyl carbinyl carbocation
	93		1,6-Methanocyclodecapentaene
	94–95		Acetylene
After November 6		OS	Letter from Charles DePuy dated Nov 6 on cyclopropyl-X solvolyses
	96		benzyne
	97		acetylene
	98–99		1,6-methanocyclodecapentaene
	100		acetylene, propylene and allyl carbocations
	101		polycyclic polyenes
November 12^c^	102		vinylcyclopropane and distorted diacetylene
	103		diacetylene
	104		benzyne
	105		divinylacetylene, 1,2-dicyclopropylethylene
	106		acetylene, nitrous oxide
	107		hydrazoic acid
	108		Ethane, isotope effects; literature on cyclopropyl-NH_2_ diazotization
	109		ethane
	110		3-vinylcyclopropene
	111		spiropentyl carbinyl carbocation
	112		diatomic carbon
	113		Benzyne and two cycloaddition references^e^
	114	OS	*cis*-2,3-Dimethylcyclopropyl radical and anion
November 20^c^,^f^	115		Literature on preparation and NMR analysis of cyclopropyl-X
	116–117	OS	Chemical pictography on cyclopropyl-X ring opening
	118		spiropentane
	119		diazarine
	120		diaziridine
	121		aromaticity
	122–123		R. C. Fort lecture
November 24	124		1,6-Methanocyclodecapentaene
Paper #1 in the Woodward–Hoffmann series, “Stereochemistry of Electrocyclic Reactions” was received in the editorial offices of the *Journal of the American Chemical Society* in Rochester, NY, on Monday, November 30, 1964.			
This notebook continues to page 154.			

a From Hoffmann, R. *Laboratory Notebook (Summer → November 1964)*, Cambridge, MA, 1964.

b OS = Related to orbital symmetry but more specifically, to electrocyclic reactions. In this time period, Hoffmann had interests in what later was termed cycloadditions and sigmatropic rearrangements but in 1965 did he begin to perform calculations related simultaneously to orbital symmetry and these other reaction types.

c Placement of this date within the notebook is approximate.

d Literature summary of Skell and Sander, stereospecificity in reactivity of cyclopropyl-X derivatives related to two-electron electrocyclizations.

e Cites Vogel (*Angew. Chem.***1963**) and Martin and Hill’s review of the Diels–Alder reaction (*Chem. Rev.***1961**, *61*, 537).

f On this page, Hoffmann “Wrote to Berson Nov. 20. . . ” implying that page 115 was written on or slightly after November 20, 1964.

**Figure 7:**
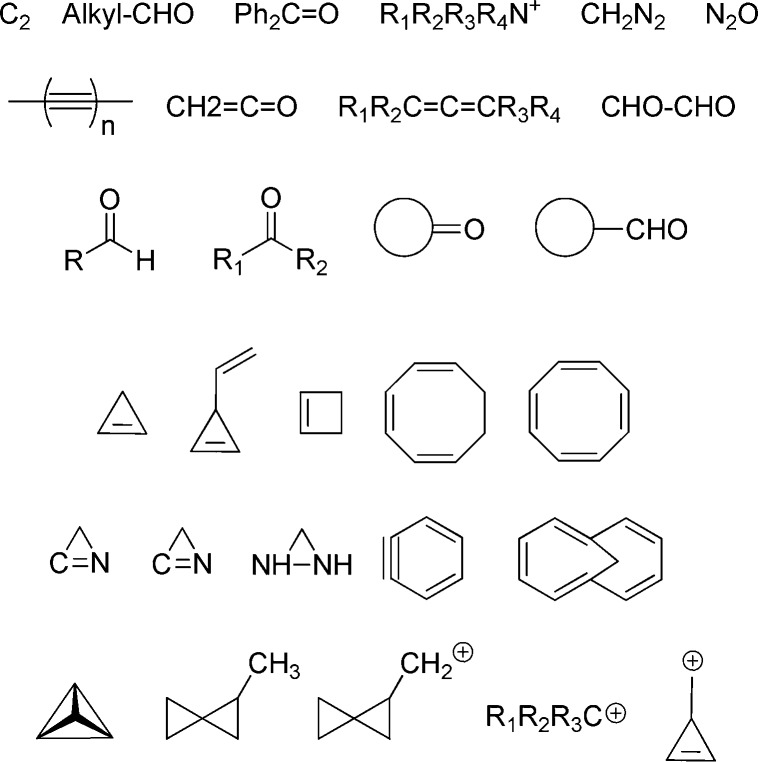
Selection of some of the structural types and molecules examined by Hoffmann during the time he was also studying electrocyclic reactions for the first W–H paper.

In addition to his calculations on systems directly related to *The Woodward Challenge* and the Woodward–Hoffmann rules, Hoffmann performed far more calculations on other organic compounds than for *The Woodward Challenge*. For a representative list of compounds that Hoffmann examined using extended Hückel from May to November 1964, see Figure 7 and Tables 1 and 2. Figure 7 looks like the structural index of the issues of *Tetrahedron* from the mid-1960s.

It is important to place Hoffmann’s extended Hückel calculations within the context of the state-of-the-art of computational chemistry in the early 1960s. From a practical standpoint, at that time the computer resources and capabilities were marginal. For example, only simple calculations of very small molecules could be performed, and these often took many hours to complete. Data were input by punch cards typically submitted at a computer center often distant from the chemistry department. (It was not even until the 1980s that a series of floppy disks was used to load a single application onto a computer—not onto a PC!) To obtain the results of one’s calculations, one would typically return to the computer center the next day and obtain massive stacks of accordion-folded 8 ^1^/_2_ × 14′′ sheets of computer output.

**Figure 8:**
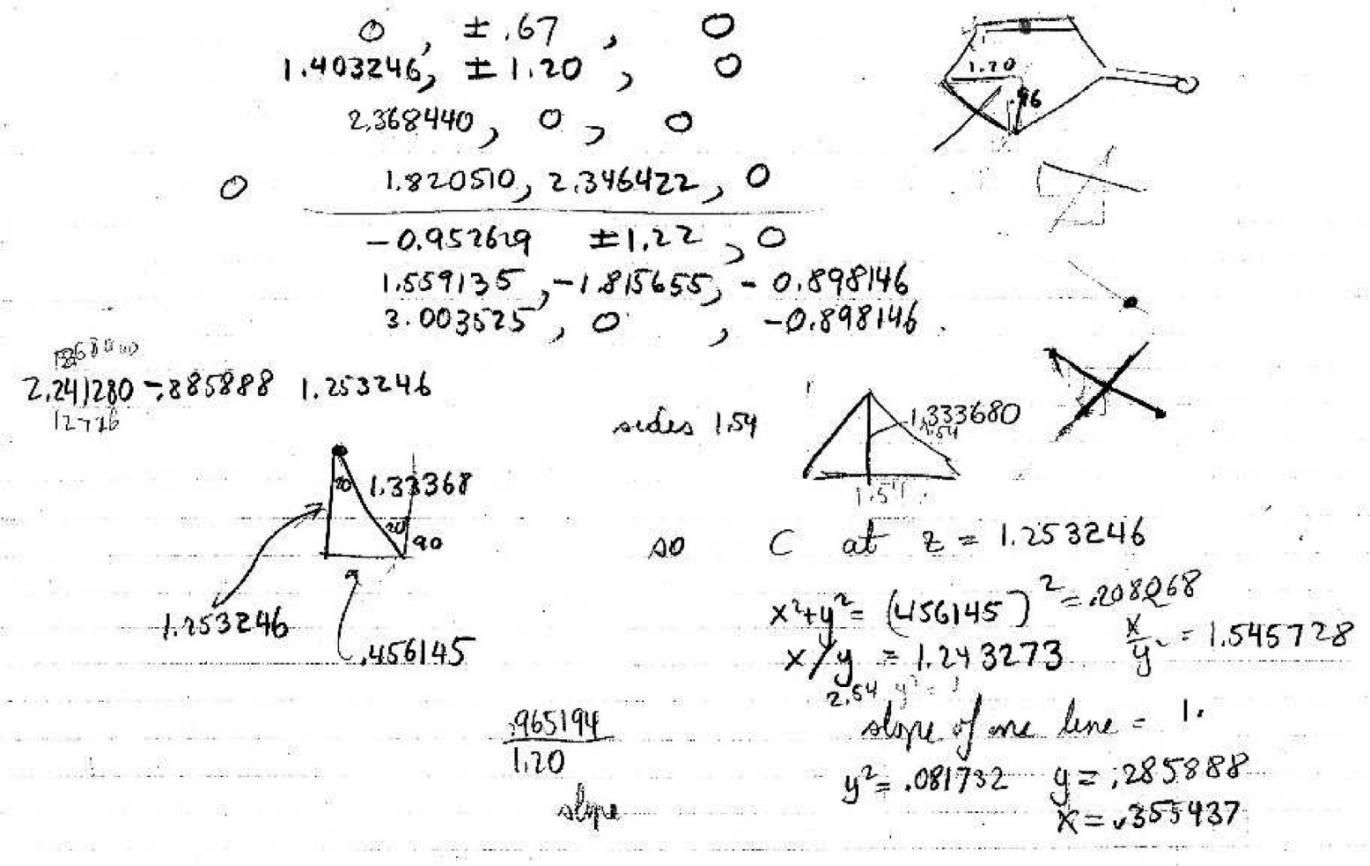
Hoffmann working his way through the input for an extended Hückel calculation on bicyclo[3.1.0]hexen-2-one performed in the spring of 1964 (page 64 from his *Early 1964* notebook).^64^ According to Hoffmann, “The calculation is in the middle of the page. I’m sitting with a slide rule, plotting out the input for cyclopentenone. I’m doing the Pythagorean Theorem. I can do square roots on a fancy mechanical calculator I bought. Today, if I gave these to my students, they would not dream of doing the Pythagorean Theorem, they’d use a coordinate-generating program and supply bond distances and dihedral angles. Or they’d just sketch in a molecule on a computer screen.”^34^

In the 1960s—indeed, even into the early 1980s—there were no graphics terminals to input one’s structures or to examine the structural output. To enter a structure, *x*,*y*,*z*-coordinates for each atom were required and obtained by tedious algebraic calculations (Figure 8) or, as this author did during his sabbatical year at Oxford when he used the extended Hückel program,^63^ structural input was a bond length, a bond angle, and a dihedral angle for each atom of the molecules. These were manually and mentally determined, atom by atom, using slide rules and mechanical calculators. One’s “*x*,*y*,*z*-coordinate structures,” determined by trigonometry and algebra, could not be confirmed by examining a graphic of the structure as is achieved trivially today. As recalled by Hoffmann,

“One could not see if one had made a trigonometry mistake (for example, an error that led to a hydrogen having the wrong coordinates, and so for instance coming out 0.2 Å from another hydrogen) until one got the output. In those days, there were no graphics. The output contained a numerical “distance matrix” that gave all the interatomic distances for all pairs of atoms. Then one could see if all the important neighbor distances were reasonable. That’s why I wrote it into the program!”^64^

Conformational energy optimization was unheard of in the 1960s or for some years later. A very large number of computations were required to derive what was hoped to be the minimum energy structure. Consequently, calculations were generally performed on a fixed structure. All of the changes in a structure in the course of a reaction had to be made by hand—it took a lot of calculations and work to arrive at a final structure. To obtain rudimentary potential energy surfaces, Hoffmann and other computational chemists of the era would vary one or two parameters at a time and manually enter the geometries structure-by-structure and, the next day, read-off the total energy or other calculated parameters from the computer printouts.

Of course, computational chemists had no idea how advances of computer power and memory would revolutionize the science. In the 1960s, there was no lack of frustration and complaints. Consider the exchange between George Whitesides, just two years into his first academic position at MIT, writing to his Ph.D. advisor John D. Roberts, on January 5, 1965, and Roberts’s response 8 days later,

George Whitesides to John D. Roberts at Caltech (1965): “M.I.T., being as it is a world center of computer application, has just decided to close permanently its only working 7090 [computer] . . . This admirable trend will soon have me reduced to abacus or toes. . . I admit that it’s comforting to learn these little skills as insurance against my old age. I wonder if key-punch operator would be a step up or down in the academic world?”^65^

Roberts’s response: “Thank you for your letter informing me of your availability as a key-punch operator—the way we are cranking out calculations, we may need you back in that capability very shortly.”^66^

Apparently, neither MIT nor Harvard of the early 1960s was state-of-the-art in computer technology, whatever that state was. In several of Hoffmann and Lipscomb’s early publications, they acknowledge the MIT Computation Center “for making available computer time” to these Harvard researchers.^25,27^

**Figure 9:**
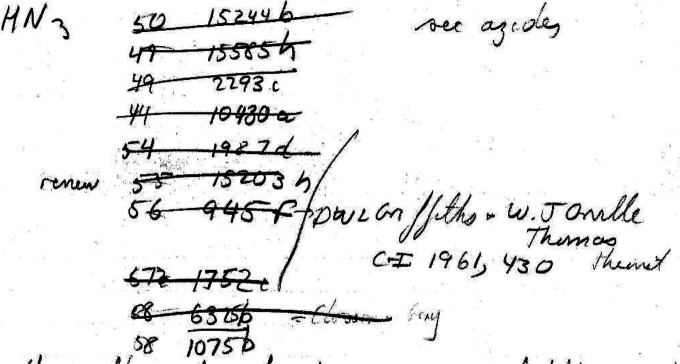
An example of one of Hoffmann’s literature searches from the fall of 1964, in this instance, of hydrazoic acid, from page 107 of the *Summer 1964 → November 1964* notebook.^67^ Chemists of a certain age will immediately recognize what Hoffmann was doing and likely produced nearly identical notes themselves from such literature searches.

Before we leave the topic of the seemingly archaic research practices of the 1960s, it is worthwhile to document the nature of literature searches of the day. SciFinder Scholar was still many decades in the future. Hundreds of volumes of *Chemical Abstracts* were found in secluded places in libraries. One searched for a compound by empirical formula or name (good luck finding the *CA* name!). One could search by subject. And one could search by author. It was not easy or pleasant; books were written instructing chemists how to conduct literature searches. Figure 9 shows one of Hoffmann’s *CA* searches in the fall of 1964 for hydrazoic acid (HN_3_), another of his non-Woodward–Hoffmann interests.

Analysis of Hoffmann’s 1964 laboratory notebooks (summarized very concisely in Tables 1 and 2) reveal a number of observations:

(1) Not all the pages report the results of calculations; many are either literature summaries or thoughts and ideas. Where there are calculations, they were all performed using the extended Hückel theory.
(2) Almost immediately after speaking with Woodward, Hoffmann began calculations on the cyclopropyl-X → allyl-X rearrangement and the 1,3-butadiene ⇌ cyclobutene valence isomerizations, as these reactions were then known.
(3) Only a single set of calculations was performed on the 1,3,5-hexatriene ⇌ 1,3-cyclohexadiene rearrangement.
(4) Of the 124 pages of notebook entries in the notebooks covering this time period, only 18 pages related to the electrocyclization paper. “This is an indication that the project with Woodward was not my top priority. I was doing whatever I liked doing. I’m still doing that 51 years later.”^34^ – Roald Hoffmann
(5) After the first burst of research on the project, ending in mid-May 1964, with the exception of one entry early in June 1964, no further calculations were performed by Hoffmann until November 1964, approximately 5.5 months later.
(6) When additional calculations were performed by Hoffmann at the very end of October or early November and then late in November, they were exclusively on the *cis*-2,3-dimethylcyclopropyl-X ⇌ 3-penten-2-yl (carbocation or anion or radical).
(7) Not once is Woodward’s name mentioned in Hoffmann’s notebooks from May 6 to the end of November 1964 nor is there any indication of any meeting or interaction involving Woodward and Hoffmann until the writing of their first paper begins. “While there is no evidence, I have a vague memory of talking to Woodward several times in this period. How else would I have communicated my results to him? I am sure such talks took place in his hallowed office, or rather its anteroom, with what I recall was a circular table.”^68^ – Roald Hoffmann
(8) Hoffmann was absent from Harvard for much of the summer and autumn of 1964. He attended several conferences during this time: the Conference on Reaction Mechanisms, Corvallis, OR (June 24–27), the International Symposium on Organic Photochemistry, Strasbourg, France (July 20–24), and the Natick Conference at the United States Army Natick Laboratories in Natick, MA (October 13–14). The Natick meeting was in part sponsored by the US National Academy of Sciences – National Research Council. It is also likely that Hoffmann visited a number of American universities during this year, in part for networking and in part to develop academic job leads. For example, there is correspondence with John D. Roberts about a visit to Caltech in late June, just before the Conference on Reaction Mechanisms.^69,70^
(9) During the summer of 1964, Hoffmann read the literature intensely. In 45 pages of a new notebook, Hoffmann records literature searches and notes from his journal readings and chemistry musings. These are likely to have taken place in Sweden. Of these 45 pages, hardly any had to do with orbital symmetry or electrocyclizations.
(10) In fact, even after the summer travel, European holiday, and months away from Harvard, Hoffmann did not return to *The Woodward Challenge* for many weeks.
(11) Hoffmann was interested in and used the EH method to calculate properties of an enormously wide range of compounds and many functional groups. His interests ranged across physical, inorganic, and organic chemistry.
(12) In terms of work effort, Hoffmann was more interested in calculations on aldehydes, ketones, cumulenes, diazirines, diazomethane, and small ring compounds than he was in solving *The Woodward Challenge*. Most of his calculations were performed on stable structures or questions of preferred geometry rather than reactions.
(13) There is no evidence that Woodward urged or pressured Hoffmann, with any degree of intensity, to work on or complete the project nor does Hoffmann remember any such urging.^41^ In other words, the laboratory notebook evidence is clear that there was no motivation—perhaps better described as no urgency—internal or external, to complete *The Woodward Challenge* and publish the results.

What, then, stimulated Hoffmann to return to the Woodward problem in November 1964 after months of inactivity on that matter? He was busy, and so was Woodward, but not with the stereochemistry of concerted reactions. We shall identify that stimulus shortly but first, we shall examine in some detail Hoffmann’s calculations that served as the basis for Hoffmann’s half of the first Woodward–Hoffmann paper.^1^

## V Hoffmann’s Computations for the Stereochemistry of Electrocyclic Reactions: May 5, 1964 to Late Spring

Following his meeting with Woodward on May 5, 1964 (see discussion above), Hoffmann demonstrated an immediate spurt of analyses and calculations regarding electrocyclizations (see Table 3 for a concise discussion of this work by notebook page). Hoffmann’s attention was first placed on the allyl cation ⇌ cyclopropyl cation transformation. That choice is intriguing, for it was not obvious then, and certainly not obvious to the physical and theoretical chemist that Hoffmann was in 1964, that solvolysis reactions could be connected to thermal and photochemical valence isomerizations. That is, a casual inspection of the four transformations in Figure 5 (Woodward’s four mysterious reactions) would suggest that eq 3 is *not* related to the other three reactions. Hoffmann thinks, and the organic chemistry community generally recognizes, that this was the insight of the genius that was Woodward, to connect chemistry from seemingly disparate provinces of organic chemistry.

**Figure 10:**
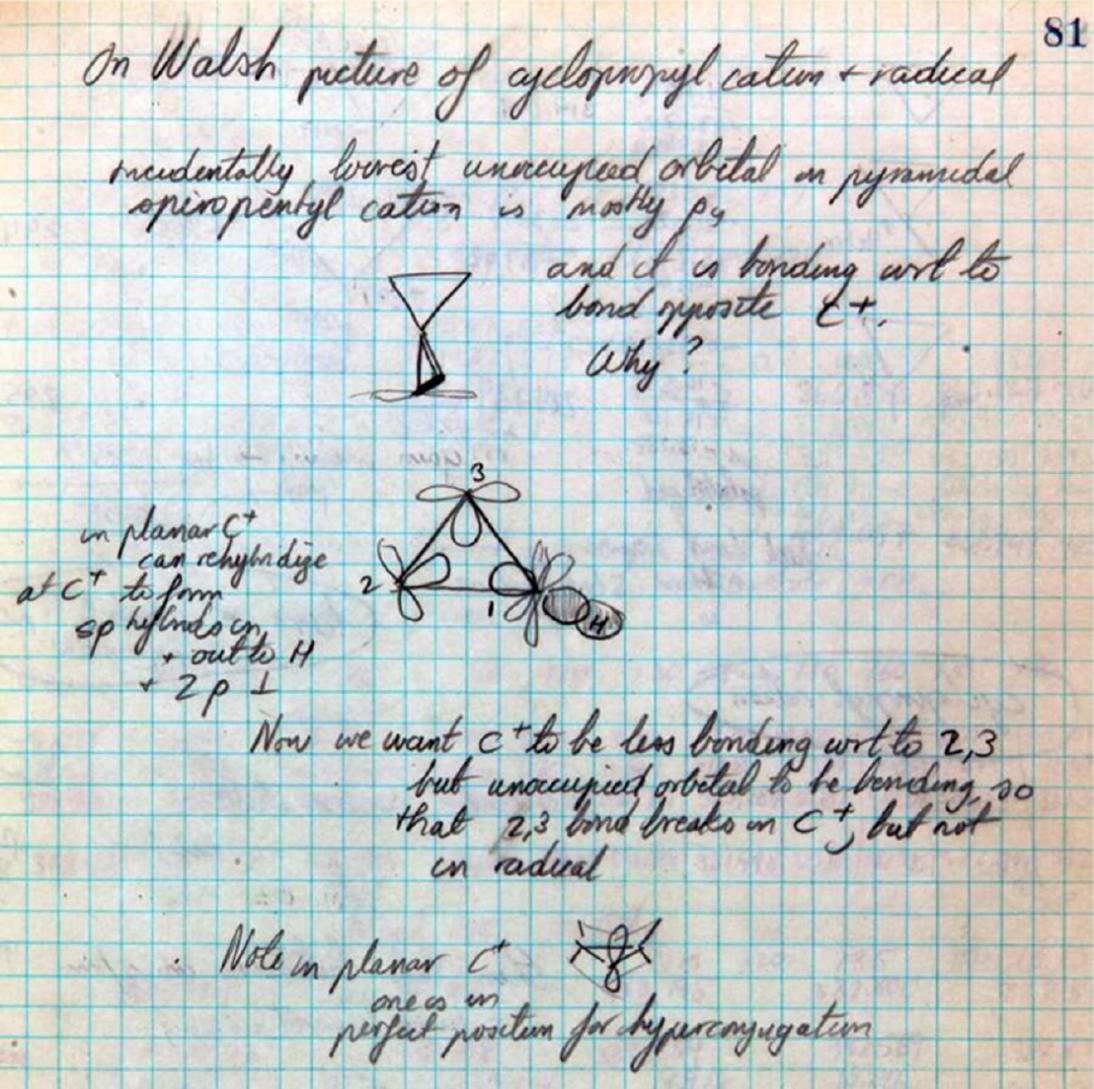
Hoffmann’s first recorded, independent thoughts on the Woodward–Hoffmann rules project, immediately after his meeting on May 5, 1964 with Woodward. These are primarily plans for future calculations on the cyclopropyl-X solvolysis. From page 81 of his notebook *Early 1964*.^77^

While the prototypical and clearest illustration of electrocyclizations is the interconversion of the four-electron system cyclobutene ⇌ 1,3-butadiene (eq 4 in Figure 5),^61,71−73^ Hoffmann’s first work was on the two-electron system, the solvolysis of cyclopropyl-X → allyl-Z.^74−76^ This valence isomerization has the computational advantage of the planarity of the cyclopropyl ring and the smallest number of carbon atoms for electrocyclic reactions. On page 81 (Figure 10), Hoffmann is “trying to get a picture of what [the bond] C_2_–C_3_ looks like as C–X breaks in a solvolysis reaction.... Now we want C^+^ to be less bonding [with respect to] 2,3 but unoccupied orbital to be bonding, so that 2,3 bond breaks....”^34^ Hoffmann also notes that a fully formed carbocation at C_1_ would lead to a planar moiety. Hoffmann’s first calculations appear on page 82, where a trigonal cyclopropyl carbocation is found to be stabilized relative to a tetrahedral carbocation.

**Figure 11:**
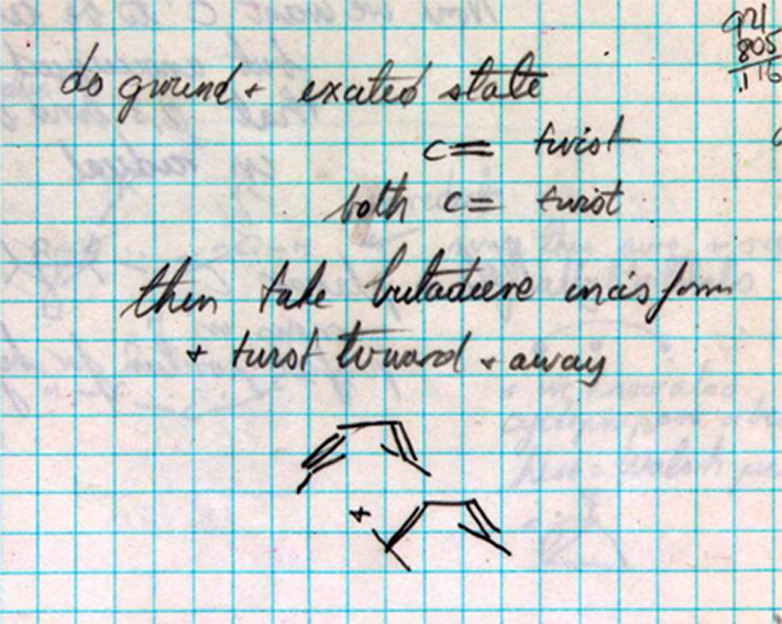
First plans by Hoffmann to perform calculations on 1,3-butadiene, an excerpt from page 82 of his *Early 1964* notebook.^78^

On page 82 (Figure 11), Hoffmann’s plan to examine both the ground state and excited states of 1,3-butadiene, twisting the terminal carbon atoms of one or both of the olefinic bonds, is described. In Hoffmann’s calculations, the excited state (ES) was defined in a simplistic one-electron way, as the energy of the configuration with one electron each in the highest occupied molecular orbital (HOMO) and the lowest unoccupied molecular orbital (LUMO) of the molecule. The terms “conrotatory” and “disrotatory” appear only in the fall of 1964. Prior to the appearance of “con” and “dis,” Hoffmann calls these motions “syn” and “anti,” respectively. And sometimes Hoffmann accompanied “syn” and “anti” by little drawings, aide-mémoires to the actual generation of input coordinates, in which he indicates whether certain hydrogens go above or below the plane of the molecule, as shown by the parallel and antiparallel lines at the bottom of Figure 11. And sometimes Hoffmann has even trouble keeping his own definitions consistent, and makes mistakes (to be uncovered a day or 51 years later!).

On page 83, Hoffmann recorded the results of his first 1,3-butadiene calculations: the energies of the lowest unoccupied molecular orbital (LUMO), the highest occupied molecular orbital (HOMO), and the total energy for the ground and excited states. For these calculations, the ∠CCC bond angles of 1,3-butadiene were kept at 120°. Potential energy curves were obtained by twisting one or both of the two methylene groups, as would happen on the way to ring closure to cyclobutene. Hoffmann was working in the one-electron framework of the extended Hückel theory. So, the “excited state” is modeled by a simple promotion of one electron from the HOMO to the LUMO. Hoffmann writes on that page,

“what we need is a potential curve for twist =CH_2_

twist both =CH_2_...

one group twisted 90° is better

than both twisted 45°”^79^

**Table 3:** Summary of Roald Hoffmann’s entries in two of his laboratory notebooks directly related to electrocyclic reactions and orbital symmetry on or after May 5, 1964. From notebooks entitled “Early 1964”^a^ and “Summer → November 1964”^b^

date	notebook page number	figure	description of analyses and/or calculations performed
“Early 1964” to Summer 1964 Laboratory Notebook			
May 5	80	4	“Talk with RBW and Applequist” and chemical pictography
	81	10	Cyclopropyl cation and radical plans and analysis, no calculations. “Trying to get a picture what C_2_–C_3_ looks like as C–X breaks in a solvolysis reaction.”^c^ Walsh diagram and cyclopropyl cation are drawn. Hoffmann beginning to think about cyclopropyl cation and direction of movement of terminal hydrogens in the opening of the ring.
	82	11	EH calculations on cyclopropyl cation with a tetrahedral and trigonal carbocation carbon with the latter found to be somewhat stabilized. Not examining either con or dis ring opening in cyclopropyl carbocation or lengthening the C_2_–C_3_ bond. Shows plans for this type of rotation in the ring closure of 1,3-butadiene.
	83		First 1,3-butadiene EH calculations. First set of calculations, twisting around the C_2_–C_3_ bond. The second set of calculations provide a limited PE curve for twisting one terminal CH_2_ group, then both CH_2_ groups.
	85	12	EH calculations on 1,3-butadiene. ∠CCC remains 120°. First calculation of con and dis rotation of the terminal CH_2_ of both double bonds. Calculations are not consistent with experimental results. Hoffmann concludes “Must run a distortion toward cyclobutene.”
	86		Graph of 1,3-butadiene reaction profile from EH calculations on page 85. No calculations
	87	13	EH calculations on 1,3-butadiene. ∠CCC is 105°. Rotation about the termini from 10° to 90°. Conrotatory motion preferred at all rotations and C_1_---C_4_ bond order is improved with 105° = ∠CCC is 120°. Plans to examine other bond angles.
	90	14	EH calculations on 1,3-butadiene, varies ∠CCC, twists both ways in GS. Situation reverses in ES. For ∠CC=C 105°, 110° and 115°, calculations of the GS show a con preference but a dis preference at larger ∠CC=C. For ∠CC=C 105°, 110° and 115°, calculations of the ES show a dis preference but a con preference at larger ∠CC=C.
	91		Graphic of EH calculation data for 1,3-butadiene from page 90. No calculations
	94	15	EH calculations on cyclobutene ring opening with ∠CCC = 105° in GS, con at all twist angles; in the ES, dis is preferred at low twist angles, con at high twist angles. With ∠CCC = 93.7° in GS, con is preferred at all twist angles.
	95	16	1,3-Cyclohexadiene ring opening calculations. At distortions that approximate C_5_–C_6_ bond cleavage to 1,3,5-hexatriene (breaking and expanding C_5_–C_6_ distance to 2.42 Å), con rotation is observed for the GS and dis rotation is calculated for the ES in accord with experiment, i.e., not at a normal C_5_–C_6_ distance in 1,3-cyclohexadiene of 1.54 Å.
	113	17	Cyclopropyl cation calculations with lengthening of C_2_–C_3_ and ∠C_2_C_1_C_3_ = 90° to simulate ring cleavage and transformation toward allyl cation, radical and anion. Rotations using syn and anti notations. For both GS and ES, the EH calculations are in accord with experiment
June 9	114		d
	152		Last page in this notebook
			
“Summer → November 1964” Laboratory Notebook			
	17	18	Literature on valence isomerizations of 1,3,5-cyclooctatriene
	34	19	Literature on cyclobutenes ring opening to 1,3-butadienes
Sept. 24	38		d
Oct. 22	76		d
	79–81	25	*cis-2*,3-Dimethylcyclopropyl carbocation (as model for solvolysis of cyclopropyl-X) calculations with some phases. One of the few orbital drawings at this early stage of research.
Nov. 3	84		d
Nov. 12	102		d
	108		Literature on DePuy-type reaction, one reference
	114		*cis-2*,3-Dimethylcyclopropyl radical and anion opening calculations. First use of “con” (and “con” and “dis” together) in these notebooks; see an isolated use on page 81 of this notebook.
Nov. 20	115		d,e
	116–117		Chemical pictography on cyclopropyl-X ring opening (two-electron electrocyclizations). Use of the term electrocyclization and disrotatory. Recognition of the extension to cycloadditions (Cope rearrangements). No calculations

a From ref (51).

b From ref (52).

c Hoffmann, R. Interviews with J. I. Seeman, Ithaca, NY, April 4 and 5, 2012.

d Research unrelated to the Woodward–Hoffmann rules. These entries are made only to indicate the date that appears on this page, as such dates are rare.

e On this page is one cyclopropyl-NH_2_ literature reference.

As would occur throughout this time period, Hoffmann immediately turned his attention to other chemistry. On the next page (page 84), Hoffmann presents a set of “Rules for [the photochemistry of] enones and dienones”.

**Figure 12:**
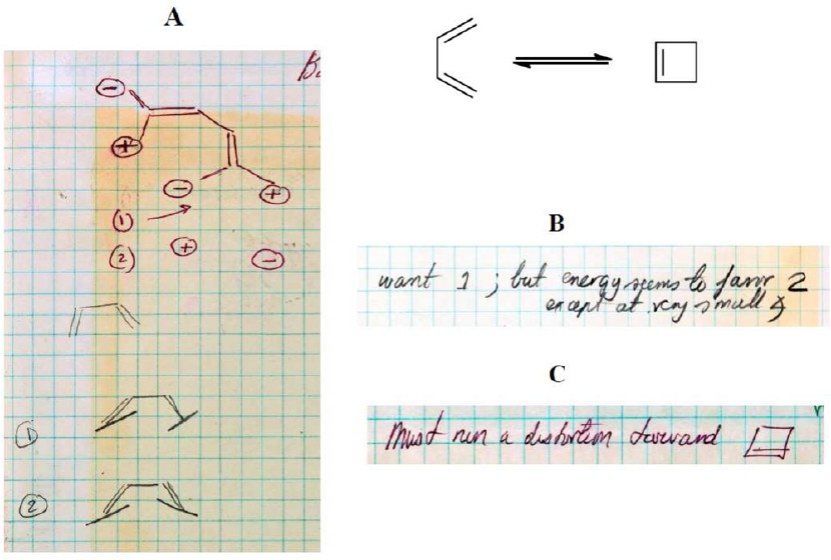
1,3-Butadiene ⇌ cyclobutene electrocyclizations. Excerpts from page 85 of Hoffmann’s *Early 1964* laboratory notebook.^83^ For these EH calculations, Hoffmann used ∠CCC = 120°. EHT total energies and bond orders for the ground and excited states of 1,3-butadiene at various twist angles for both con and dis motion are not reproduced in this figure. (A) Hoffmann’s first graphic of twisting motions about C_1_ and C_4_ as required for the cyclization. The encircled positive and negative signs and the parallel and antiparallel lines at the methylene carbons C_1_ and C_4_ refer to the twisting motions, i.e., the direction of rotation, not orbital phases. Plus refers to “up” motion and minus means “down” motion of terminal hydrogens relative to the diene carbon plane. (B) Hoffmann says “want 1” referring to the inconsistency between experiment and with Woodward’s Frontier Orbital-based argument. (C) Hoffmann concludes that the computational model must be distorted such that the ∠CCC is more like that in cyclobutene, that is, closer to 90° rather than 120°. The ChemDraw graphic at the top is added for the benefit of the readers.

Pages 85–87 contain the results of the first calculations of conrotatory and disrotatory motion for 1,3-butadiene. Figure 12 contains excerpts from page 85 of Hoffmann’s notebook. The ∠CCC bond angle of 1,3-butadiene was still fixed at 120°. In Figure 12, the encircled positive and negative signs reflect, for the calculations, the direction of motion of the hydrogens at the terminal carbons, not molecular orbital information. The parallel and antiparallel lines at the methylene carbons C_1_ and C_4_ refer to conrotatory and disrotatory motions, respectively. The extended Hückel total energies are still *inconsistent* with the experimental results. Hoffmann notebook reports “want 1” referring to a conrotatory motion but the calculations favor “2.”

In searching for an explanation for the inconsistency between experiment (and Woodward’s frontier orbital argument) and extended Hückel theory, Hoffmann began to think about the reaction as a chemist would, not as a mathematician. He began to understand that the twisting rotations of the terminal carbons in the absence of bringing these two carbons closer together fails to represent the transition state for ring closure. C_1_---C_4_ bond formation is not possible when the ∠CCC = 120°. The ∠CC=C in cyclobutene is 94°, far from 120°. Hoffmann recalls

“In my very first EH calculations on the reaction, I try twisting the 1,3-butadiene to cyclobutene and I get the wrong preference (disrotatory favored instead of conrotatory). But I don’t give up. I realize that the reaction coordinate must involve the C–C–C angle variations as well as the twisting of the termini. On page 87, I then proceed to do this. I run the same termini twisting but with a C–C–C bond angle of 105° instead of 120°. I am learning how to construct reaction paths—there was nothing to guide me. Oh, yes, there were calculations on A + B–C going to A–B + C, but it’s a long way from that to electrocyclic reactions.”^81^

and

“The payback for the cost of [the C–C–C] angular distortion is the [C_1_–C_4_] bond formation.”^82^

**Figure 13:**
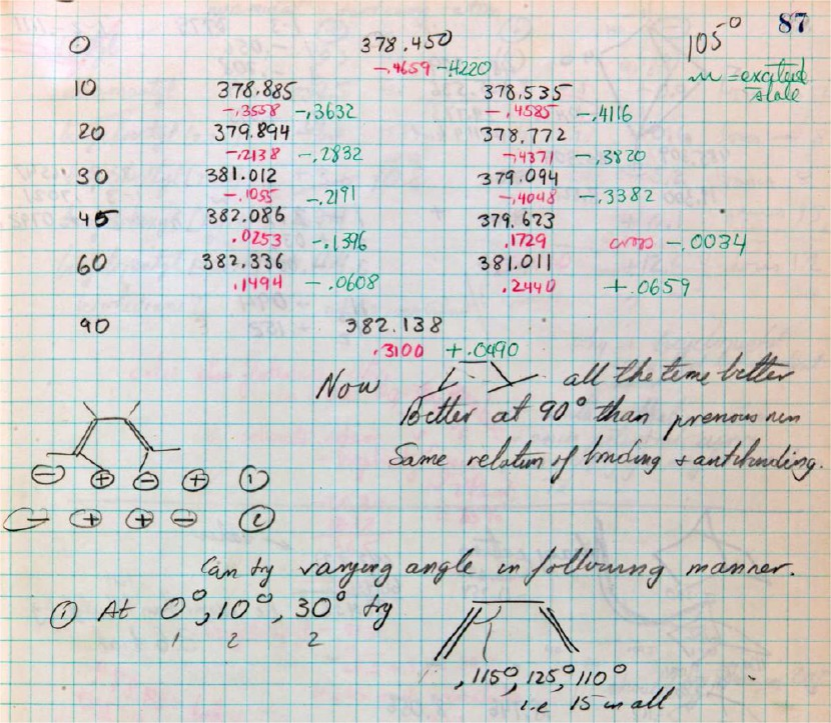
Page 87 from Hoffmann’s *Early 1964* notebook.^84^ EH calculations for 1,3-butadiene in the ground state with a ∠CCC of 105°. The bottom left structure represents the direction of rotation, with positive referring to upward motion of the hydrogen. Motion “1” is thus conrotatory and “2” disrotatory. For the numbers in black, the left column is for the negative of the total energy for conrotatory motion, the right column for disrotatory motion. The numbers directly under the total energies are likely to be C_1_---C_4_ overlap populations (like bond orders), red for the ground state and green for the first excited state. As the reaction proceeds (going from planar from 0° to 90°), the total energy for conrotatory motion is always lower (more stable) for disrotatory motion consistent with the experimental results. Note Hoffmann’s plans at the bottom of this page to vary the ∠CCC.

Page 87 reports the total energy for the EH calculation of 1,3-butadiene in the ground state with a ∠CCC of 105°, closer to the bond angle in cyclobutene. The methylene groups are rotated simultaneously from 0° to 90° (Figure 13). *This is the first EH calculation for the 1,3-butadiene* ⇌ *cyclobutene electrocyclization in which the experimental results were correctly modeled by the theory.* In addition, the C_1_–C_4_ bond order (technically a Mulliken overlap population, a bond index popularized by Lipscomb and Hoffmann^25−27^) of 0.3100 (reported directly under the total energy of −382.138) at 90° is larger than 0.1362 reported on page 85 (i.e., when ∠CCC 105° versus ∠CCC = 120°). Several of Hoffmann’s comments are particularly relevant:

“Now [conrotatory rotation] all the time better. Better at 90° then previous run. Same relation of bonding & antibonding.” (See Figure 13.)^84^

and, later, reflecting on what was done,

“No one had studied complex potential energy surfaces before—I made a start, learned how to construct the approach to the transition state for the reaction.... In thinking about the bonding, I am also developing a primitive frontier orbital theory without making a connection, one I should have or could have made, to [Kenichi] Fukui’s work on frontier orbitals.”^34^

With this first correct theoretical model for an electrocyclic reaction, did Hoffmann proceed with other electrocyclic reactions, e.g., cyclopropyl-X ⇌ allyl carbocation or 1,3-cyclobutadiene ⇌ 1,3,5-hexatriene? No! Hoffmann returned to EH calculations of bridgehead and tertiary carbocations, bicyclo[1.1.1]pentane, and pyramidal tertiary cations (pages 88–89). Recently, Hoffmann reflected on this nonlinear research path:

“I go back to other things. [Notebook pages 88 and 89]This is an indication that the project with Woodward is not the top priority. I am doing whatever I like doing. Before that, my obsessions were ketones and organic photochemistry. Now, carbonium ions.”^34^

**Figure 14:**
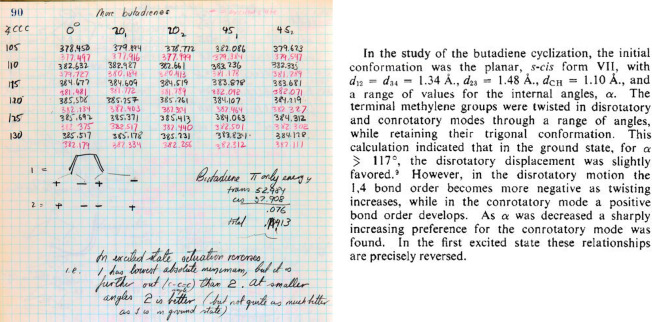
(Left) Page 90 from Hoffmann’s *Early 1964* notebook.^86^ This matrix tabulates EH total energies as a function of ∠CCC (rows) and rotation about the termini of the double bonds (columns). The columns are identified by angle (in degrees) with either a subscript “1” (conrotatory motion) or a subscript “2” (disrotatory motion). In each “cell,” the top energy (in black) is for the ground state; the bottom energy (in red) is for the excited state. (Right) An excerpt of the corresponding section from Woodward and Hoffmann’s *Stereochemistry of Electrocyclic Reactions*.^1^

But Hoffmann did not forget what he planned to do on page 87. Notebook pages 90 and 91 report the data for 1,3-butadiene at ∠CCC from 105° to 130° at 5° intervals, with twisting of the termini of both double bonds at 0°, 20°, and 45° for both con and dis motion, for both the ground states and excited states (Figure 14, left). At ∠CC=C of 105°, 110°, and 115°, calculations of the ground state of the distorted 1,3-butadiene show a con preference but a dis preference at larger ∠CC=C which are less relevant for the electrocyclization reaction toward cyclobutene. At ∠CC=C of 105°, 110°, and 115°, calculations of the ES show a dis preference but, in five of six cases, a con preference at larger ∠CC=C which again are less relevant for the electrocyclization reaction. These calculations appear in the *Stereochemistry of Electrocyclic Reactions* paper^1^ (Figure 14, right).

Reaction surface calculations in 1964 were primitive, at best; the reality of today’s capabilities could not have been imagined at the time. Hoffmann’s output samples very incomplete reaction surfaces: 16 points for four reactions (one point each for the ground state reaction, con and dis motion; and one point each for the excited state, con and dis motion. There is no attempt to locate the relevant transition states nor is there any attempt to find the minimum energy conformation of either 1,3-butadiene or cyclobutene.

What immediately follows are EH calculations on methyl spiropentane, literature data of a piperidine quaternary salt (page 92) and EH calculations on the reaction of an allyl anion with a proton (page 93). As for the latter, Hoffmann notes,

“I’m not thinking of sigmatropic reactions. It’s too soon. I’m taking an allyl anion and approaching a proton, not cyclizing an allyl anion. Why am I doing these calculations? It could be that I was prompted by some chemistry Göran Bergson in Sweden is doing.”^34^

**Figure 15:**
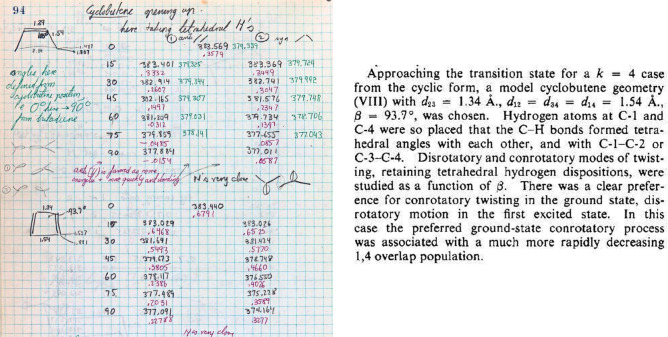
(Left) Page 94 from Hoffmann’s *Early 1964* notebook.^87^ EH calculations for the ring opening of cyclobutene to 1,3-butadiene. The columns are identified by angle (in degrees) with either a subscript “1 anti” (conrotatory motion) or “2 syn” (disrotatory motion). The numbers in black are for the ground state and in green are for the excited state. The numbers in red are the overlap population between C_1_ and C_4_ in the ground state. (Right) An excerpt of the corresponding section from the *Stereochemistry of Electrocyclic Reactions* paper.^1^

“Cyclobutene opening up” is the title of page 94 (Figure 15). The EH calculations for the ground and excited states (∠CCC = 105°) and ground states (∠CCC = 93.7°) are shown in Figure 15, left graphic, top and bottom tabulations, respectively). The EH calculations are *consistent* with the experimental data. Note the multicolor presentation in the laboratory notebook. An excerpt from the Woodward–Hoffmann paper on the ring opening of cyclobutene to 1,3-butadiene is shown in Figure 15, right. Note also the nomenclature used on page 94: “anti //” referring to what was eventually called “con” and “syn/\” referring to what was eventually called “dis” (see third line of text in page 94). The publication indicates that both the ground state (GS) and excited state (ES) were examined at ∠CCC = 93.7°. The data on page 94 only include ES geometry with ∠CCC = 105° (Figure 15, left graphic, top data).

Where are the missing data? Could it be that not all of Hoffmann’s calculations are in these notebooks? He has retained some, but not all, of the original computer output, and there are few scribblings on these pages. Details of construction of the *x*,*y*,*z*-coordinates for the substrates are mostly absent from the notebooks (but see one instance shown in Figure 8). Hoffmann recalls a small program he wrote for generating input geometries. Given the detail in which he recorded the calculations in the notebooks, as illustrated in several of the figures herein, Hoffmann thinks “It is likely that all there was is in the notebooks.”^30^

For the GS calculations, at ∠CCC = 105° (not reported in the *JACS* paper), con rotation is *preferred*, in the pairwise comparison, at every twisting angle (the 382.165 at 45° assumed to be 308.165). For the ES calculations, at ∠CCC = 105° (also not reported in the *JACS* paper), dis rotation is preferred (in the pairwise comparison) at 15°, 30°, and 45° but con is preferred at 60° and 75°.

For the GS calculations, at ∠CCC = 93.7° (reported in the *JACS* paper), con rotation is preferred (in the pairwise comparison) at every twisting angle. As stated above, the ES calculations at ∠CCC = 93.7° are not recorded in the notebook but are reported in the *JACS* paper (see Figure 15).

Important here is that Hoffmann was considering the ring-opening reaction. He could do so because the reaction was thought to be concerted, but it is not certain that at the time his knowledge of organic reaction mechanisms was deep enough for him to understand that consideration. The frontier orbital HOMO/LUMO argument is applicable from the polyene side. There was no literature precedent for examining a reaction from both sides; this may be the first instance of a theoretician doing so. Could it be that Hoffmann was uncertain about his conclusions starting from the polyene, that is, the ring-closing reaction, and looked for support from a calculation that started from the ring, that is, the ring-opening reaction? Hoffmann speculates that that may have been the case.^85^

**Figure 16:**
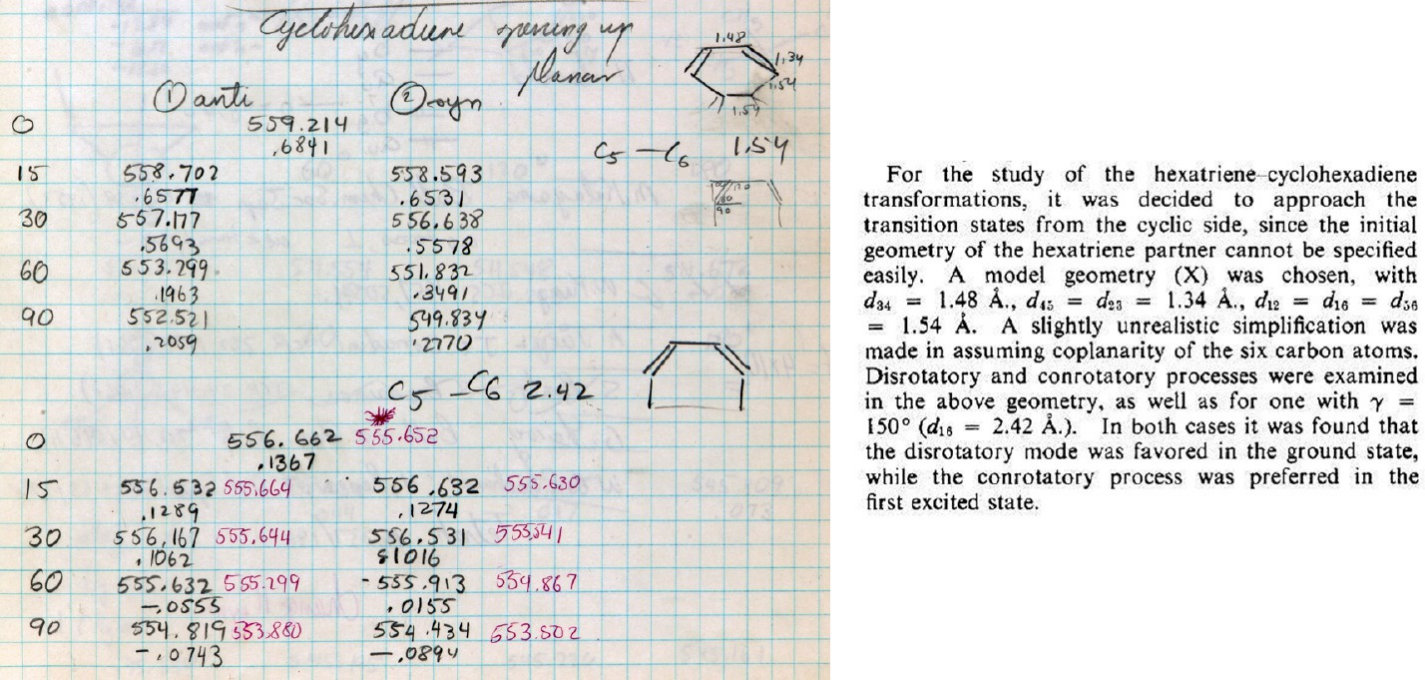
(Left) Bottom section of page 95 of Hoffmann’s *Early 1964* notebook.^88^ Extended Hückel calculations for distorted conformations of the ground state (numbers in black) and excited state of 1,3-cyclohexadiene (numbers in red). The top calculation has a fixed C_5_–C_6_ distance of 1.54 Å, while the bottom has that distance set at 2.42 Å. The top numbers are the negative of total energies, the bottom are C_5_–C_6_ bond orders. (Right) Excerpt from the Woodward–Hoffmann *Electrocyclization* paper. Geometries for the calculations are as described in the *JACS* excerpt. “1 anti” = “//” = con. “2 syn” = “\ /” = dis.

The next calculation (page 95) is on glyoxal (top of page 95) followed by the only calculations on the 1,3-cyclohexadienes ⇌ 1,3,5-hexatriene electrocyclization reported in Hoffmann’s notebooks (Figure 16). Two sets of EH calculations were reported on conformationally distorted 1,3-cyclohexadienes, one with C_5_–C_6_ length 1.54 Å (i.e., the length of a normal C–C single bond) and the second at 2.42 Å (i.e., with the C_5_–C_6_ bond substantially broken as in the ring opening reaction). At 1.54 Å, for the GS (the ES was not reported on this page), con rotation is favored at all twists examined (15°, 30°, 60°, and 90°), contrary to the experimental results. However, at the C_5_–C_6_ length 2.42 Å—more reasonable, in that it better resembles a transition state on the way to 1,3,5-hexatriene—dis was favored at all angles other than 90°.^88^ For the ES at 2.42 Å, con twisting was favored at all angles examined. *These results—both for the ground state and the excited state—show an imperfect consistency between experiment and the W–H explanation.*

With the understanding that the reaction model must incorporate geometries that are somewhat intermediate between the reactant and the product, i.e., must resemble the transition-state geometry, Hoffmann returned to the cyclopropyl-X ⇌ allyl carbocation reaction. Previously (laboratory notebook page 82, Figure 11), Hoffmann performed EH calculations on cyclopropyl cation while retaining the normal bond angles of cyclopropane, i.e., no motion toward allyl cation. In addition, there was no twisting of the terminal methylene groups, i.e., no motion toward allyl cation. In other words, the earliest calculations did not simulate either a con or a syn motion toward a ring-cleaved product.

**Figure 17:**
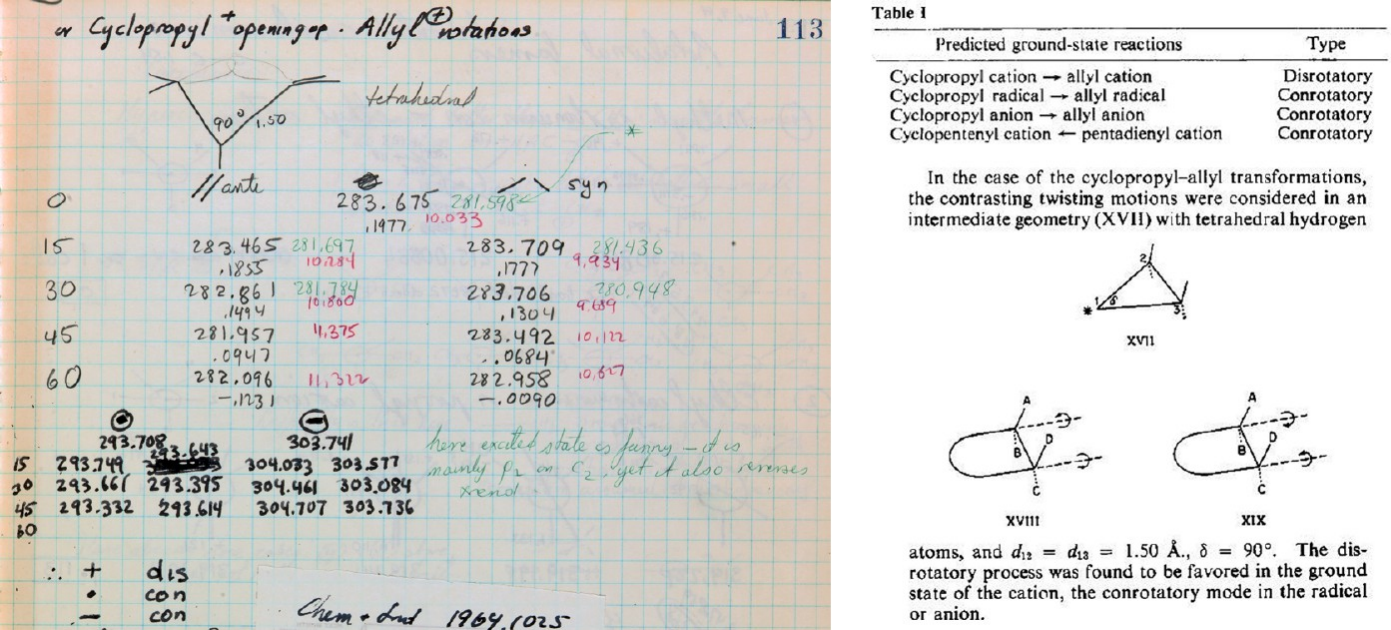
(Left) Page 113 of Hoffmann’s *Early 1964* notebook. Extended Hückel calculations for distorted conformations of the ground state and excited state of cyclopropyl carbocation (at the top) and radical and anion (bolder black numbers, at the bottom) with ∠C_2_–C_1_-C_3_ = 90°, C_2_ geometry fixed tetrahedral, and consequently C_2_–C_3_ bond being elongated. The negative of the total energies are in black and, for the excited state, in green; with bond orders below. At the very bottom right of Hoffmann’s notebook page, he writes “here excited state is funny—it is mainly p_2_ on C_2_. yet it also reverses trend.” (Right) Excerpts from the Woodward–Hoffmann *Electrocyclization* paper. Geometries for the calculations described in the *JACS* excerpt. “anti” = “//” = con. “syn” = “\ /” = dis.

As shown in Figure 17, Hoffmann performed EH on three partially cleaved cyclopropyl models (cation, radical, and anion) in their ground and excited states. In all instances, carbon C_1_ was fixed in a tetrahedral geometry with ∠C_2_C_1_C_3_ = 90°, effectively lengthening the C_2_–C_3_ bond and simulating a structure advanced along the reaction path (see structure at the top left of Figure 17). For the cation, rotations of the terminal methylene groups at 15°, 30°, 45°, and 60° were examined along with the unrotated substrate. See Figure 17: the left column in each comparison is “//” referring to “anti” and con motion; the right column in each comparison is “/ \” referring to “syn” and dis motion. *For every rotational angle*, for all six substrates (cation, radical, and anion; GS and ES for each), the lower of the pairwise-comparison of the EH total energy fit the experimental data or the frontier orbital argument.

Following these extended Hückel calculations performed in the first half of May and one calculation just prior to June 9, and shown in Figures 13–17—the only new *computational data included in the first Woodward–Hoffmann paper except forcis-2,3-dimethylcyclopropyl-X*—Hoffmann moved on to other calculations. At this time, mid-May 1964, there is no evidence of any effort by Woodward or Hoffmann to draft a manuscript or to publish the results. There is no evidence that Hoffmann’s calculations were discussed with Woodward until October or November. The EH calculations reported on page 113 (Figure 17) were performed the first week in June. Nearly 50 more pages of Hoffmann’s *Early 1964* notebook would be filled with extended Hückel calculations performed well into July 1964 on other topics, before Hoffmann left Harvard for Europe (see Table 1), including calculations on:

• acyclic and cyclic ketones and aldehydes
• acetylenes
• allenes
• cumulenes and other hydrocarbons
• heterocycles, e.g., diazirines
• reactive intermediates such as carbenes and carbocations

As recalled by Hoffmann,

“I’m just episodic in my research interests, working on several problems at a time, jumping one to another in what moves me. It’s clear that I am in transition between physical chemistry and organic chemistry. Take cumulenes, part organic, part physical. Ditto for the organic photochemistry I look at. The organic part of my calculations, of my thinking is growing, will grow. I perform calculations on a compound, then I leave it, after one page.”^34^

Hoffmann knew that he was going to travel much of the summer of 1964 (see discussion above). Hoffmann would not be able to perform any EH calculations during that time; they had to be done at Harvard. Nonetheless, the approaching time away from Cambridge did not spur into action. He was not driven to draft what would be his portion of the *Electrocyclization* paper.^1^ Nor is there any evidence that Woodward urged Hoffmann to complete the work; perhaps Woodward may not even have been aware that Hoffmann was to leave the country for two months. As for Hoffmann, other functional groups and molecular frameworks were of more interest to him, and his notebooks (Table 1) record those interests. Hoffmann clearly did not appreciate the breakthrough that was the solution to *The Woodward Challenge*.

**Scheme 1:**
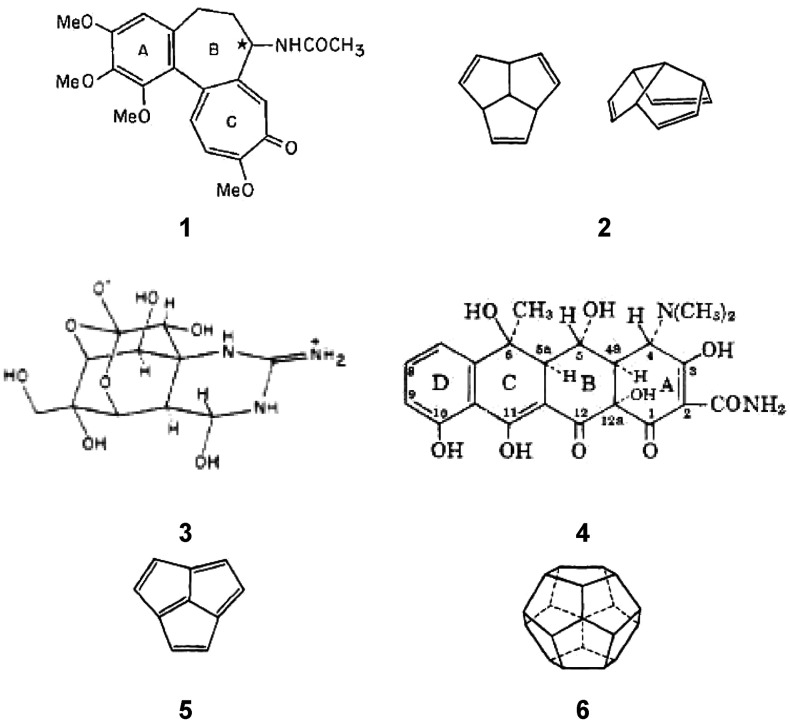
Structures of Four Compounds (**1**–**4**) on Which Woodward Published between May and November, 1964, One of Which (**2**) Was Considered To Be a Possible Precursor of Acepentylene (**5**) and Dodecahedrane (**6**) The pictographs are reproduced from Woodward’s papers (see Table 4).

One might ask: where was Woodward all these months, from May 5 until November, and what was he doing? Did Woodward not care about the project he offered to Hoffmann? Woodward was busy working, too, with his attention focused in many other directions. Table 4 lists Woodward’s papers submitted for publication in that time period. At that time, he was writing and submitting papers on the total synthesis of colchicine (**1**), the synthesis of triquinacene (**2**), and the structures of tetrodotoxin (**3**) and oxytetracycline (**4**) (Scheme 1). Woodward and members of his group were working on the Harvard portion of the Woodward–Eschenmoser collaborative total synthesis of vitamin B_12_ among other projects. The Woodward Research Institute at Ciba AG had just opened in 1963 in Basel, Switzerland.^89^ Woodward was surely spending time in Switzerland along with his normal extensive travels around the world. More on Woodward and Hoffmann’s nonorbital symmetry activities during this time period will be presented in Section X.

**Table 4:** Woodward’s Publications Submitted between May 1964 and November 1964

title	reference	submission date (1964)
1963–1964: First Year of Operation of the Woodward Research Institute (WRI) at Ciba AG in Basel, Switzerland, Founded in 1963^a^		
A Total Synthesis of Colchicine (**1**)	Woodward, R. B. A Total Synthesis of Colchicine, In *Harvey Lectures, Series 59 (1973–1964)*; Academic Press: New York, 1965; p 31–47.	ca. spring^b^
Triquinacene (**2**)	Woodward, R. B.; Fukunaga, T.; Kelly, R. C. *J. Am. Chem. Soc.***1964**, 86, 3162–3164.	June 18
The Structure of Tetrodotoxin (**3**)	Woodward, R. B.; Gougoutas, J. Z. *J. Am. Chem. Soc.***1964**, 86, 5030.	September 17
Digital Computer Program for Calculation of Molecular Formulas	Usher, D. A.; Gougoutas, J. Z.; Woodward, R. B. *Anal. Chem.***1965**, 37, 330–332.	October 20
The Stereochemistry at C–S in Oxytetracycline (**4**)	von Witteneau, M. S.; Blackwood, R. K.; Conover, L. H.; Glauert, R. H.; Woodward, R. B. *J. Am. Chem. Soc.***1965**, 87, 134–135.	November 14

a For some discussion of the Woodward Research Institute and leading references, see: Craig, G. W. *Helv. Chim. Acta***2011**, 94, 923–946.

b The paper was based on the Harvey Lecture which was delivered on October 17, 1963. This book in which the paper appeared was published in 1965. The manuscript likely was written and production completed in the spring of 1964.

As a side note, the synthesis of triquinacene (**2)** had embedded in it another intellectual opportunity, in addition to being a possible precursors of acepentylene (**5**) and dodecahedrane (**6**). And Woodward recognized this, too. In his paper, Woodward stated,

“triquinacene possesses three double bonds so situated in fixed positions as to provide valuable information about the postulated phenomenon of homoaromaticity and about the nature and extent of homoallylic participation in olefinic reactivity. A study of the capacity of triquinacene to form metal complexes would also be of special interest.”^90^

The triquinacene paper was submitted on June 18, 1964, approximately six weeks after Woodward’s meeting with Hoffmann. Clearly, Woodward could have asked Hoffmann to consult on the theoretical aspects of electronic interactions through space or through bonds. However, he did not.^85^

Now we return to the Woodward–Hoffmann collaboration. Is it reasonable that Woodward did not pressure Hoffmann for results, or even request updates on his work? Was Woodward totally in the dark regarding Hoffmann’s work, if indeed—as far as Woodward knew—there was any work at all? In several interviews, Hoffmann said he could not remember any contact with Woodward over these many months. As there is no written evidence of interactions with or urgings by Woodward to work on the project or begin writing the first paper. In his reading a final draft of this manuscript, in large part to confirm the accuracy of the “facts” and of his quotes included herein, Hoffmann wrote,

“I have changed my mind, I have a vague recollection of meeting with him several times, but not often.”^68^

Evidently, whatever meetings there were between Woodward and Hoffmann, they were not memorable.

Furthermore, there is no indication that Woodward felt he was in competition—nor in a rush to publish—with his peer group, including E. J. Corey. On May 4, 1964, the day before Woodward’s meeting with Hoffmann, Corey^54,55,91^ had stated that he divulged to Woodward that he had conceived valid mechanistic explanations for the stereochemistry of concerted reactions. (Others had already come to this same mechanistic proposal, namely Luitzen J. Oosterhoff^92^ and Fukui.^93^) Apparently, Woodward was either unmoved by this competition or did not consider it important enough to publish Hoffmann’s and his results immediately. Or Woodward was simply focused on other chemistry. As will be shown below, no calculations performed by Hoffmann after early June 1964 were *required* for a first Woodward–Hoffmann publication. (It is true that the calculations on 2,3-dimethylcyclopropyl-X solvolyses in late October and November 1964 enhanced *Stereochemistry of Electrocyclic Reactions*, but this author posits that they were not required for its publication and Hoffmann agrees.^7^)

## VI Hoffmann’s Research on the Stereochemistry of Electrocyclic Reactions: Summer to Mid-November 1964

While in Europe during the summer of 1964, Hoffmann did not abandon his science. But he did let this future Nobel Prize research sit unattended, mostly gathering dust at Harvard. As detailed in the second row of Table 2, Hoffmann filled the first 45 pages of a new notebook *Summer* → *November 1964* with notes from numerous literature searches and from his journal readings. While in Sweden with his family, Hoffmann had access to the libraries of the University of Stockholm and the Royal Institute of Technology, and he used both. A wide range of organic chemistry topics were recorded. A fuller discussion of these pages is far outside the scope of this paper, in large part because they have no relationship with the stereochemistry of concerted reactions or ultimately with the Woodward–Hoffmann rules. However, three pages are worthy of note.

**Figure 18:**
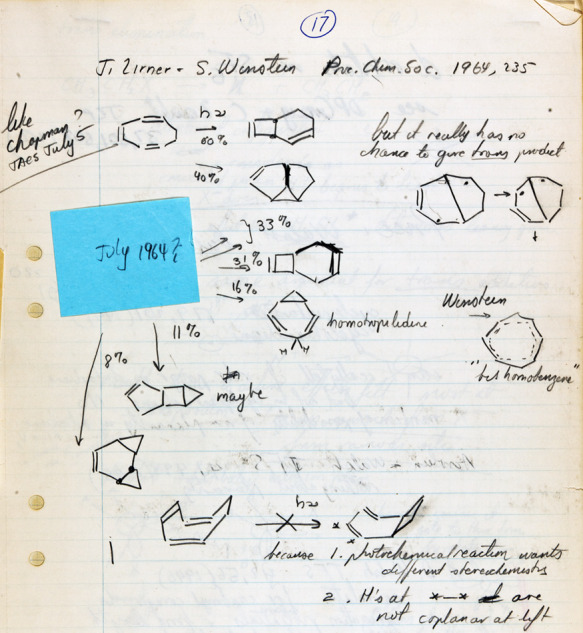
Page 17 of Hoffmann’s *Summer* → *November 1964* notebook.^100^ Of particular importance is Hoffmann’s recognition (not reported by Winstein^94,95^) of a Woodward–Hoffmann “alternation effect” in the chemistry of 1,3,5-cyclohexatriene (**8**) shown more clearly in Scheme 2. See the text for further discussion.

**Scheme 2:**
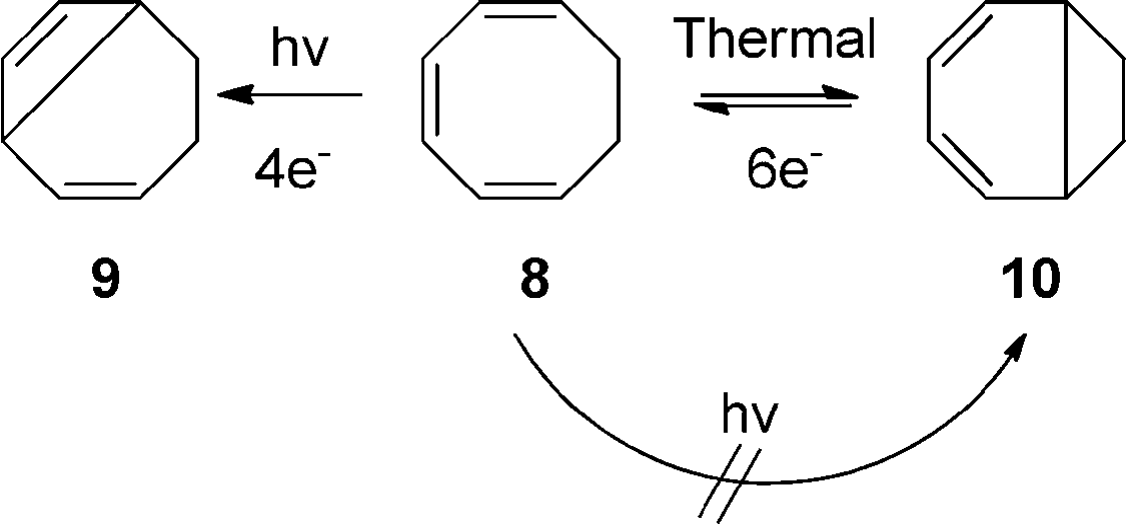
Electrocyclizations in the 1,3,5-Cyclohexatriene Series from the Work of Saul Winstein Note the alternation effect, as the reactions in this scheme are all disrotatory but two are thermal and one is photochemical.

**Figure 19:**
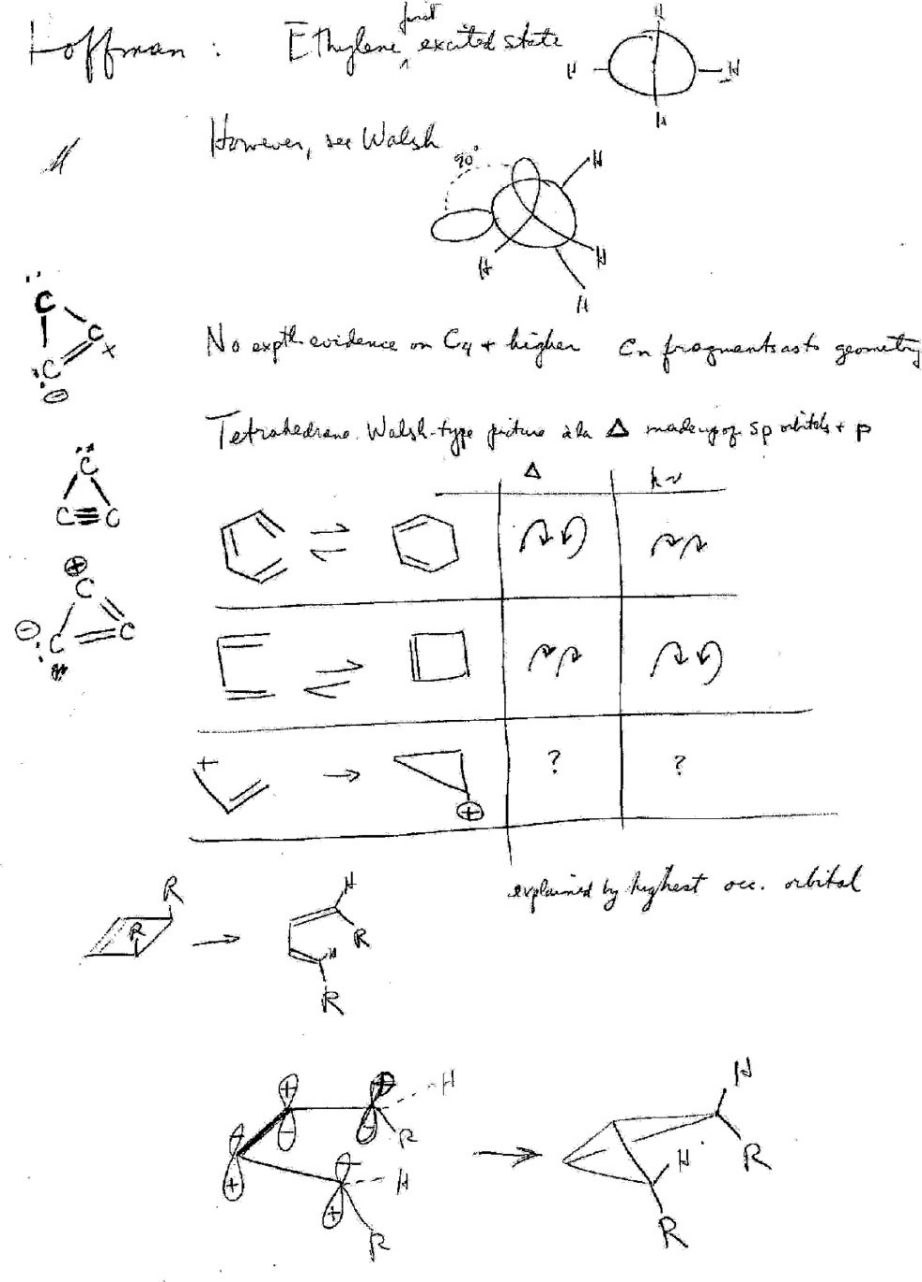
Jerome Berson’s notes of Roald Hoffmann’s impromptu lecture, likely on Tuesday, October 13, 1964, at the Natick Conference. Note the misspelling of Hoffmann’s name at the very top of the page.

On page 17 of his *Summer 1964* laboratory notebook (Figure 18), Hoffmann presents some literature examples from the laboratory of Saul Winstein: a pair of related reactions of 1,3,5-cyclooctatriene (**8**), one being a four-electron valence isomerization to form **9** and the other a six-electron isomerization to form **10** (both disrotatory motions) (Scheme 2).^94,95^ Hoffmann wrote on this page, “photochemical reaction wants different stereochemistry”, indicating he understood the stereochemical requirements of thermally and photochemically allowed 4e^–^ and 6e^–^ electrocyclizations. This alternating feature so characteristic of the orbital symmetry chemistry^96^ apparently was not recognized—at least, not documented in his papers—by the eminent physical organic chemist Winstein^19,97^ until after the revelations of Hoffmann at the Natick meeting (see Figure 19).^1^ Winstein cites Hoffmann’s revelation in a 1965 publication with David Glass that was received by *Tetrahedron Letters* on December 22, 1964, submitted over a month after the Natick meeting.^96^

**Figure 20:**
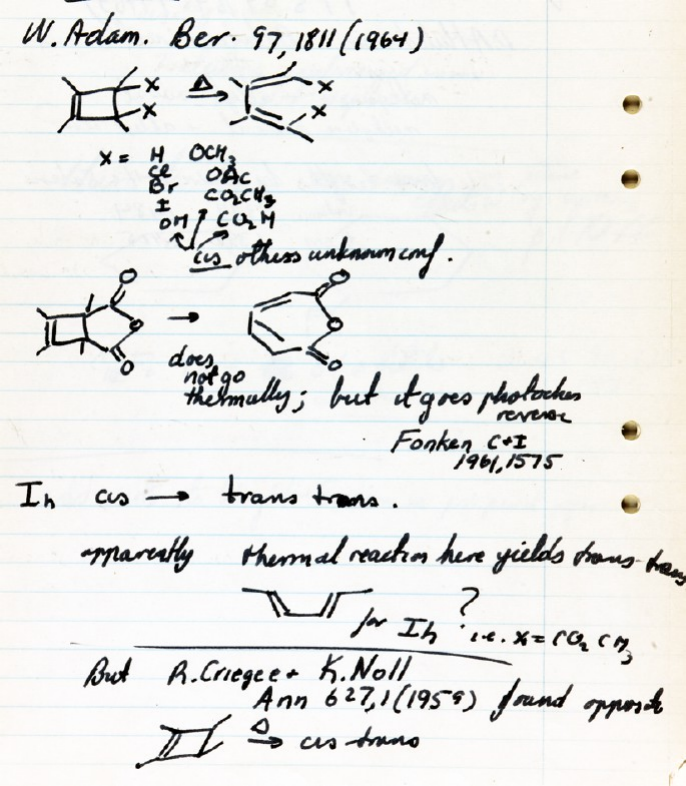
Page 34 of Hoffmann’s *Summer* → *November 1964* notebook.^101^ Note the several examples of 4e^–^ electrocyclizations on this page. See the text for further discussion.

On page 34 (Figure 20), Hoffmann records several four-electron valence isomerizations from the literature. This page embodies the most dramatic, even most informing experimental data that demanded explanation, i.e., it rehearses *The Woodward Challenge* (Figure 4). Waldemar Adam’s pyrolysis of substituted cyclobutenes,^98^ Gerhard Fonken’s photochemical but not thermal 4e^–^ isomerizations (another example of alternation),^99^ and Criegee and Noll’s ring opening to the less stable (*E*,*Z*)-1,3-butadiene^61^ are documented on this page.

Most curiously, why was Hoffmann *just now*—in the summer of 1964, months after documenting his May 5 meeting with Woodward (Figure 4) and performing many calculations on the various valence isomerizations (for example, Figures 10–17)—recording the key literature on the topic? Several hypotheses could explain this observation. One, that Hoffmann was satisfied initially (May 5) with Woodward’s summary (Figure 4) and he, Hoffmann, did not feel the need to go to the original literature to get going on the problem. Or two, Hoffmann just did not know the original work, and here he is finding it, by himself, with his reading in the literature. Neither of these explanations is entirely satisfactory, given that Hoffmann was now two years from a Harvard Ph.D. and thus was somewhat experienced, “even if not completely at home in the lore of organic chemistry”.^85^ And because even as he documents in these notebooks, Hoffmann had much experience conducting literature searches using *Chemical Abstracts* (see Figure 9).

In this and the previous section, Hoffmann’s research on *The Woodward Challenge* has been examined in great detail, with a reasonable emphasis on what Hoffmann did. We now ask, what did Hoffmann *not do*? As Hoffmann relates,

“It’s staggering that in my calculations on electrocyclic reactions, there is no concentration on the HOMO and LUMO and their nature, their nodal properties. It is as if I’m proceeding in a parallel universe with RBW: he using the frontier orbital argument, and I just doing what comes naturally to me, total energy calculations. With this sometimes reliable method. I choose to trust the extended Hückel method. And we paste these universes together in the first paper. Also I’m just not aware of the power of the frontier orbital way of thinking; it is not until the cycloaddition and sigmatropic reactions came into focus that I see this.”^53^

As shown in Table 2, after returning from Europe, Hoffmann performed extended Hückel calculations on many different classes of compounds in September and late October or early November 1964 (pages 46–78) but not a single calculation related to electrocyclic reactions and *The Woodward Challenge* for months. But suddenly, almost out-of-the-blue, on pages 79–81 of the *Summer 1964* laboratory notebook, Hoffmann reported EH calculations on *cis*-2,3-dimethylcyclopropyl carbocation.

What stirred the blood and caused a return to *The Woodward Challenge*, some four months in dormancy for Hoffmann? As discussed in the next section, the record suggests it was Criegee, Rolf Huisgen, Charles DePuy, and Winstein—not Woodward—who provided the provocation and ultimately the stimulation to complete the research and initiate the drafting of the paper. It is hard to imagine that Woodward had forgotten his initiative with Hoffmann. Indeed, Woodward had many reasons to be continuously eager and even impatient about publishing the mechanistic solution to these mysterious reactions. Apparently, however, Woodward was not sufficiently compelled to move the Woodward–Hoffmann work toward publication.

## VII What Stimulated the Writing of the Paper?

Hoffmann publically revealed the explanation to the no-mechanism conundrum—to *The Woodward Challenge*—several, if not many months prior to the publication of the first Woodward–Hoffmann paper. This fact, combined with Hoffmann’s eventual recognition of the worldwide interest in the problem, ultimately catalyzed the writing and publication of *Stereochemistry of Electrocyclic Reactions*.

**Figure 21:**
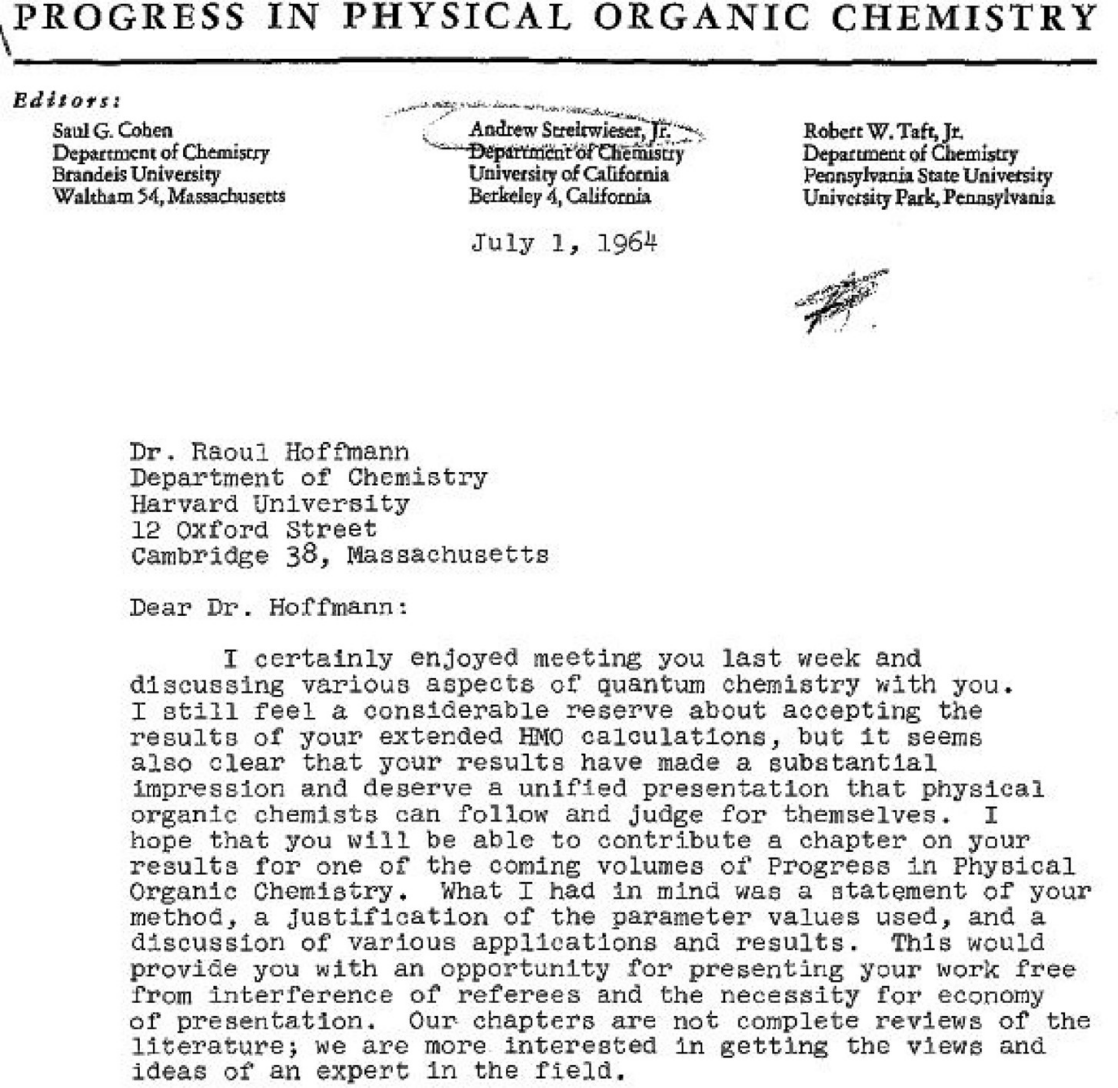
Excerpt from Andrew Streitwieser’s letter to Roald Hoffmann on July 1, 1964. Streitwieser’s attitude toward Hoffmann’s extended Hückel theory is clearly ambivalent. At the time, Streitwieser was one of the editors of the series of books *Progress in Physical Organic Chemistry*. Note the misspelling of “Roald.”

Hoffmann’s presentation of the use and utility of the extended Hückel method—but likely not yet the conservation of orbital symmetry story—at the Conference on Reaction Mechanisms in June 1964 received mixed reviews.^102,103^ Shortly after the conference, Andrew Streitwieser, an eminent experimentalist who turned half of his research into theory and was the author of a prominent textbook on molecular orbital theory,^104^ wrote a somewhat critical letter to Hoffmann (Figure 21). Streitwieser wrote that he has “considerable reserve about accepting the results of your extended HMO calculations...”.^102^ Perhaps it is fortunate that Woodward—and many other organic chemists—were unaware of Streitwieser’s skepticism of the extended Hückel method. That Hoffmann was relatively unknown at the time is again reflected in Streitwieser’s misspelling of Hoffmann’s first name. Streitwieser wrote to “Raoul” instead of “Roald.”

Streitwieser’s negative opinion of extended Hückel theory expressed to Hoffmann in 1964 moderated over time. Streitwieser recently stated,

“I recall talking with Roald around that time about EHT. I expressed my concern that it was a low level theory compared to the *ab initio* programs just becoming available—my group started such computations around that time. But Roald was very clever in his use of what was his personal EHT tool. He made effective use of *relative* energies and the shapes of MOs—the HOMO and LUMO especially are adequately given even by low level EHT. But I didn’t realize any of this at that time.”^105^

Then came the long period of Woodward–Hoffmann dormancy, as discussed above.

Several events occurred in mid-October 1964 that reawakened Hoffmann’s interest and stimulated his return to *The Woodward Challenge.* Indeed, within 6 weeks of this renewed interest, the first Woodward–Hoffmann paper^1^ would be written, submitted, and accepted for publication in the *Journal of the American Chemical Society*.

On October 13–14, Hoffmann attended the Eighth Organic Chemistry Conference held at the United States Army Natick Laboratories.^106^ At the meeting, DePuy spoke on “Intermolecular Cis Eliminations” and discussed the solvolysis of cyclopropyl-X compounds, two-electron valence isomerizations among Woodward’s four mysterious reactions (Figure 5) included in *The Woodward Challenge* (Figure 4). Criegee spoke on “Valence Isomerizations of Cyclobutenes,” the four-electron valence isomerizations and another of Woodward’s four mysterious reactions within *The Woodward Challenge*. Winstein spoke on “Some Studies with Cyclic Trienes and Related Compounds” (see Figure 18) including both two-electron and four-electron valence isomerizations. Two other lectures likely included discussion of concerted reactions and valence isomerizations: Huisgen’s talk on “Cycloadditions of Mesoionic Aromatic Compounds” and Michael P. Cava’s talk on “Recent Developments in the Chemistry of Condensed Cyclobutadienes.”

Hoffmann was surely stimulated by these lectures on valence isomerizations, all fitting into the pattern of *The Woodward Challenge* and all lacking a successful mechanistic explanation. By this time, Hoffmann had a unified theory that would explain all of these reactions. Having previously experienced giving a “talk from the floor” at the Conference on Reaction Mechanisms just several months previously in June in Corvallis, Oregon, on some of his extended Hückel calculation,^103^ Hoffmann was emboldened in front of this eager audience to make another not-on-the-agenda presentation. As Hoffmann recalls,

“I went to the [Natick] meeting because it was nearby, and the speakers were of interest. At this point, I had not been to many meetings, maybe two—a boron–nitrogen meeting, and the Organic Symposium [in Corvallis, OR in June 1964]. RBW did not go to the meeting. I remember vaguely that there was a good audience, over 100 people. My intervention was spontaneous, after Criegee’s talk. He reported his own work on an electrocyclic opening, carefully studied, and expressed puzzlement. I asked a question, or rather made a comment, asking if I could go up to the blackboard or paper board and explain the reaction. I am guessing that I didn’t give *the ganze megillah* [“the whole story”] about the calculations, but used the frontier orbital explanation. I am sure I mentioned it was joint work with Woodward. It was after this comment that Chuck DePuy approached me and asked me what I thought about the two disrotatory modes in cyclopropyl-X solvolysis.”^82^ [See below for Hoffmann’s follow-up to DePuy’s question.]

DePuy remembered that, after Criegee’s talk, Hoffmann presented the concept that was shortly to appear in *Stereochemistry of Electrocyclic Reactions* and showed how those concepts explained Criegee’s results.^107^ Within the Jerome Berson archives is a rather detailed recording of Hoffmann’s presentation—which was given either at the Conference on Reaction Mechanisms or, more likely and now agreed upon by both Berson and Hoffmann, at the Natick meeting (Figure 19) (another example of no dating!).^108^

Hoffmann apparently began his presentation by discussing the geometry of ethylene in its first excited state. Then, according to Berson’s notes, Hoffmann discussed cumulenes and tetrahedrane. Then came electrocyclic reactions with a table showing the alternating stereochemistries (thermal versus photochemical; four-electron versus six-electron systems). Berson writes in his meeting notes, “explained by highest occ[upied] orbital” and draws the HOMO of 1,3-butadiene. Either Hoffmann or Berson then considered the ring closure of 1,3-butadiene to bicyclo[1.1.0]butane via ψ_2_ even though that reaction occurs photochemically where the HOMO would be ψ_3_ (+ – – +).

Berson misspelled Hoffmann’s name in Figure 19, not a rare instance of this spelling error. Of course, Hoffmann was rather unknown to the organic chemical community in 1964 so spelling an unfamiliar name incorrectly—both the “Roald” and the “Hoffmann”—was not unusual. In fact, it was not unusual *even after* he became rather well-known.

There was a hungry community, eager to pounce on the theme. Woodward and Hoffmann’s *Stereochemistry of Electrocyclic Reactions* was to cause a stampede of research aimed at testing its predictions as well as motivation to supply alternative explanations underlying the stereochemistry of concerted reactions. The latter include Zimmerman’s Möbius mechanism^109,110^ and Dewar’s transition-state aromaticity mechanism,^111^ topics that will be covered by this author in a subsequent publication. Surely, a mechanistic revelation at a scientific meeting would also stimulate the competition.

Hoffmann acted apparently with neither concern for proprietorship nor with approval for such a preliminary public announcement from his senior collaborator, R. B. Woodward. All this—Hoffmann’s delays in moving forward on the project, the worldwide interest in and competition regarding this chemistry, Hoffmann’s presentation unsanctioned by Woodward, and Hoffmann’s understanding at some level of the ownership issues raised in May or June 1964 by E. J. Corey^91^—renders Hoffmann’s spontaneous sharing of his unpublished research results rather remarkable.

**Figure 22:**
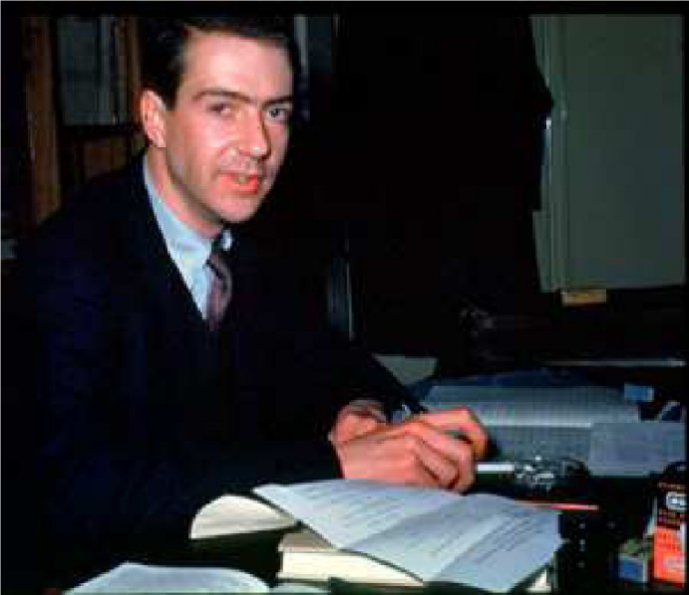
Hugh Christopher Longuet-Higgins in his office, Cambridge, England, ca. 1965. Photograph courtesy John D. Roberts and the Chemical Heritage Foundation.

Remarkable but perhaps not unique. Without Hoffmann’s knowledge, Woodward was sharing his (see Figure 4 and Woodward’s self-reporting)^51^—and by happenstance, Oosterhoff’s^92^ and Corey’s (according to Corey)^54,55^—frontier orbital explanations outside his own research group. One instance is known. Woodward had discussed some portion of these results with the eminent theoretician H. C. (Christopher) Longuet-Higgins (Figure 22) in 1964 in Cambridge, England,^112^ who then began his own theoretical studies on the topic.^113^

Hoffmann has questioned whether “a few months ago” —Longuet-Higgins’s characterization of when Woodward told him of his ideas, in Longuet-Higgins’s letter dated December 28, 1964—might have been before he and Woodward began their collaboration, in May 1964—which was, in fact, eight months prior to Longuet-Higgins’s letter. I challenged Hoffmann’s question, “Might ‘a few months’ be more than eight months?” Hoffmann replied,

“It’s just conversation [between Longuet-Higgins and Woodward], and people’s perceptions of time are flexible. I want you to apply to normal human beings humane and psychologically perceptive criteria of behavior and expression. Which means that they can be imprecise in their expression.”^114^

A full discussion of Longuet-Higgins, his interactions with Woodward, and his contributions to the orbital symmetry story is outside the scope of this paper and will be presented elsewhere.

**Figure 23:**
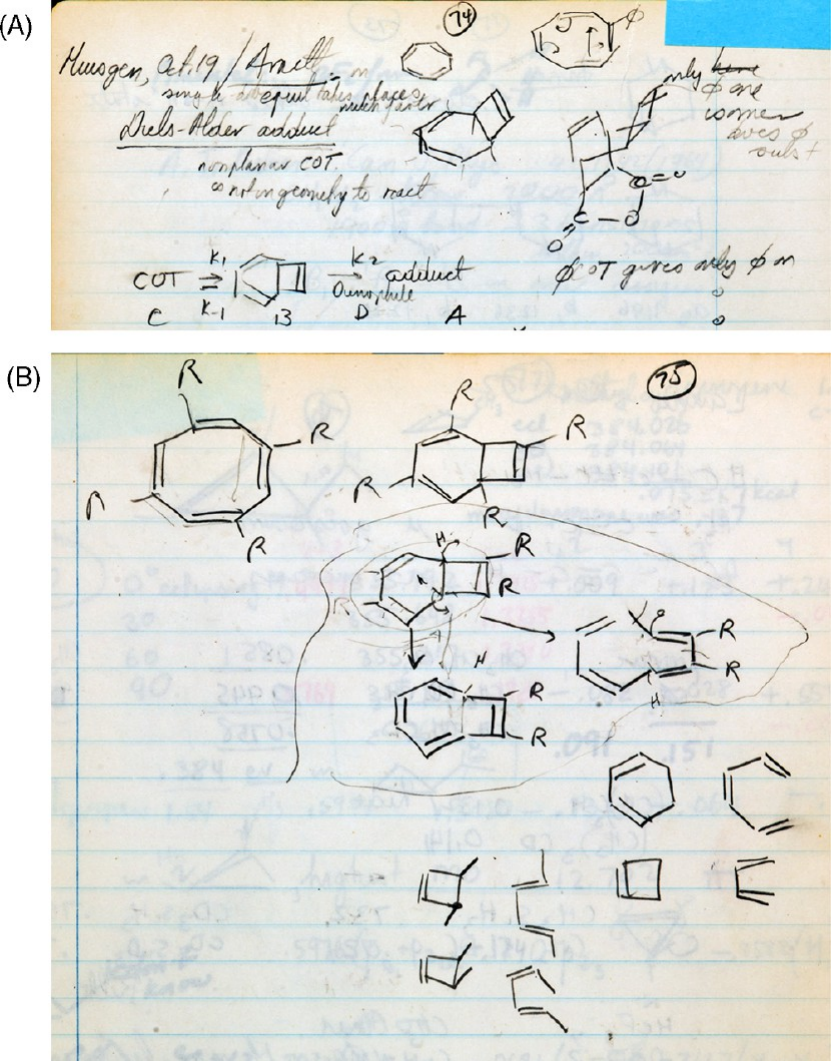
From Hoffmann’s *Summer* → *November 1964* notebook, notes from a lecture given by Rolf Huisgen at Harvard on October 19, 1964. (A) Excerpt from page 74. (B) Page 75. Huisgen is describing his experimental results of various thermal and photochemical electrocyclizations. At the bottom of page 75, Hoffmann draws analogous valence isomerizations from the laboratories of Rudolf Criegee,^115−117^ Vogel,^72,118,119^ Egbert Havinga,^92^ William G. Dauben,^120^ Gerhard Fonken,^99,121^ and others.

There were further stimulations for the writing of the first W–H paper. On October 19th, within a week of the Natick conference, Huisgen presented a lecture at Harvard that included some of the same material he (Huisgen) had also presented at Natick. As shown in Figure 23 (pages 74 and 75 from Hoffmann’s *Summer* → *November 1964* notebook), Hoffmann’s close attention to Huisgen’s lecture is evident. Of particular note, Hoffmann draws the cyclobutene ⇌ 1,3-butadiene and the 1,3-cyclohexadiene ⇌ 1,3,5-hexatriene electrocyclizations as well as the Criegee and Emanuel Vogel experiments on *cis*- and *trans*-3,4-cyclobutene thermal ring openings. These embody one of Woodward’s four mysterious reactions (Figure 5) and one of the reactions in *The Woodward Challenge* (Figure 4).

**Figure 24:**
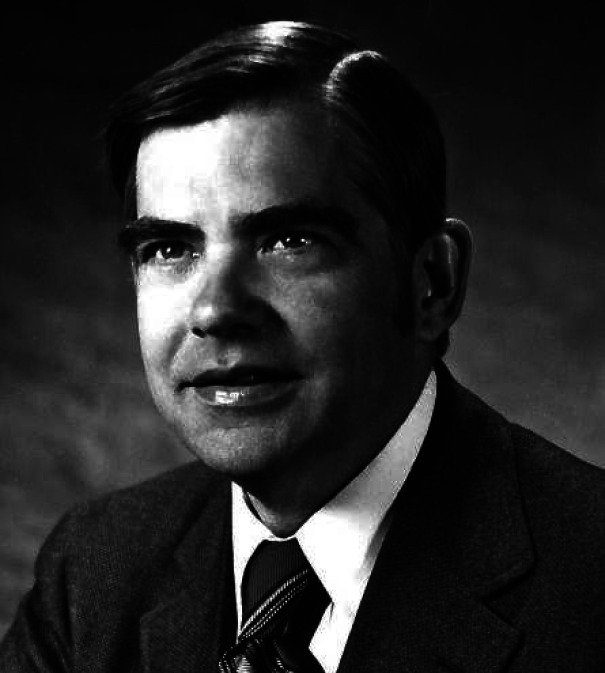
Charles H. DePuy, ca. 1965. Photograph courtesy of the late C. H. DePuy.

Clearly, Hoffmann sees the relationship between the results presented at the Natick conference, Huisgen’s presentation at Harvard, and his (Hoffmann’s) research with Woodward. This author speculates—and Hoffmann agrees—that chemistry discussed at Natick and by Huisgen at Harvard provided the stimulus to write *The Stereochemistry of Electrocyclic Reactions*. Further stimulus was provided by the immediate and enthusiastic responses to Hoffmann’s talk at Natick. Several chemists cited Hoffmann’s Natick disclosures in their early 1965 papers,^73,96,122^ and at least one chemist (Charles DePuy, Figure 24) provided such important feedback that Hoffmann performed additional calculations which were included in the first W–H paper (discussed below).

This author postulates that the various drafts of the first W–H paper, and there were several that are discussed below, were written subsequent to Rolf Huisgen’s October 19th lecture. Unfortunately, it is not possible to provide a perfect chronology of the events of that time. As noted previously, few pages of Hoffmann’s notebooks are dated; neither are there notes nor letters between Woodward and Hoffmann discussing the various drafts of that first Woodward–Hoffmann paper. In contrast, after Hoffmann’s move to Cornell University in July 1965, there are some dated letters between Woodward and Hoffmann regarding subsequent papers but many of Hoffmann’s letters are also undated.

First, after the Natick Conference and before the end of November when the first W–H paper was submitted, Hoffmann performed calculations on numerous types of compounds *unrelated* to the Woodward–Hoffmann rules. During this time period, 80% of his laboratory notebook pages are unrelated to *The Woodward Challenge* (see Table 2). In other words, even during this time period, with indication of worldwide competition and interest and his own public announcement of his and Woodward’s thinking and results, Hoffmann was not focused entirely on *The Woodward Challenge*.

**Figure 25:**
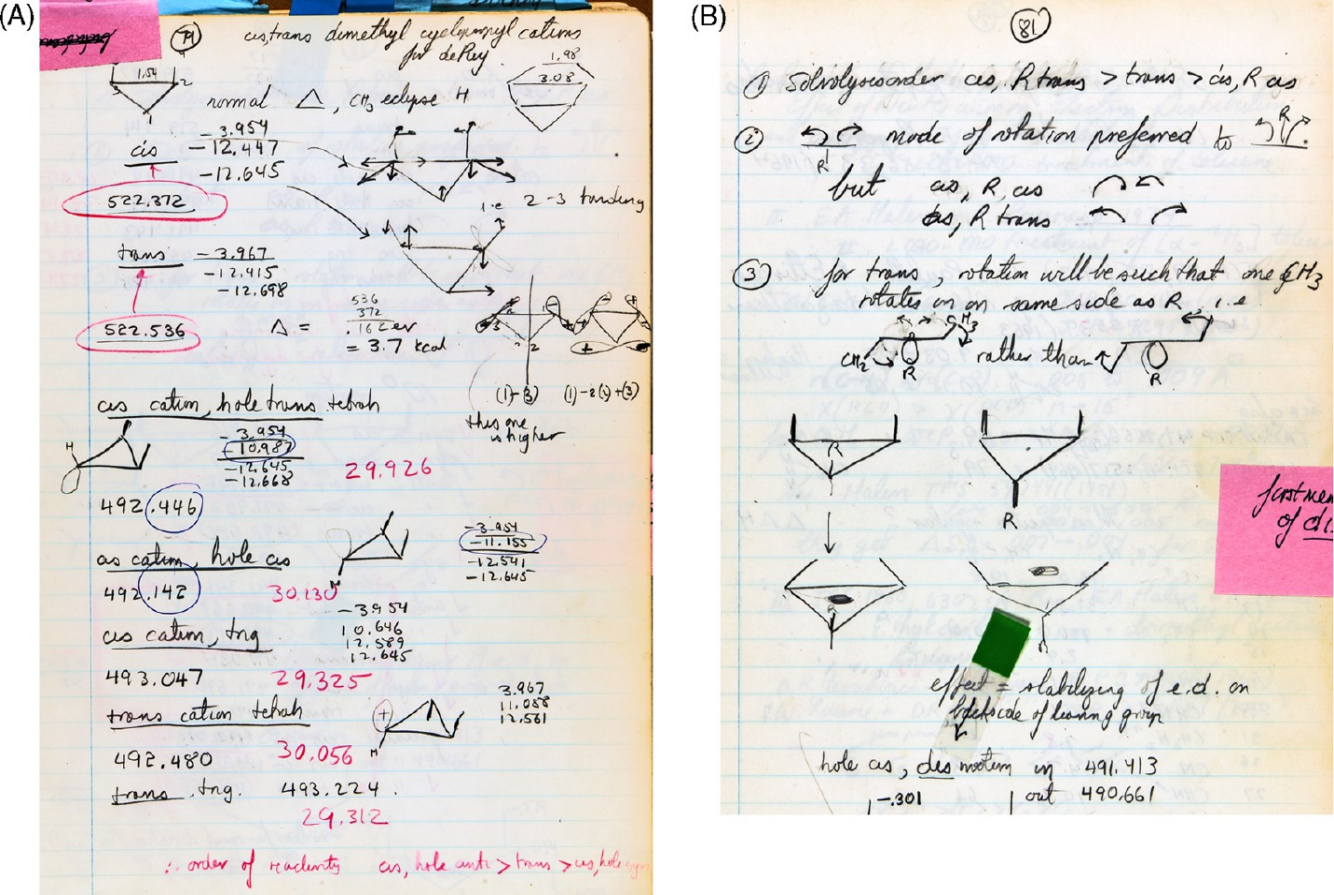
Excerpts from Roald Hoffmann’s *Summer* → *November 1964* laboratory notebook. (A) From page 79, “cis, trans dimethyl cyclopropyl cations for dePuy [sic]” In these extended Hückel calculations, the modified geometry of the “ring” is shown at the upper right corner, modeling the solvolysis reaction and concurrent (concerted) ring opening by the lengthening of the C_2_–C_3_ bond. The terminology “cis cation, hole trans, tetrah” refers to the *cis* orientation of the methyl groups, the *trans* orientation of the carbocation empty orbital relative to the methyl groups, and a tetrahedral configuration at C_1_. (B) At the top of page 81, Hoffmann records his three major conclusions or predictions that will appear in the first W–H paper. A pink note, added recently to this page, reads “first mention of dis,” referring to Hoffmann’s statement on this page, “dis motion,” the preferred mode of rotation of the C_2_ and C_3_ atoms and the substituents on those atoms.

**Scheme 3:**
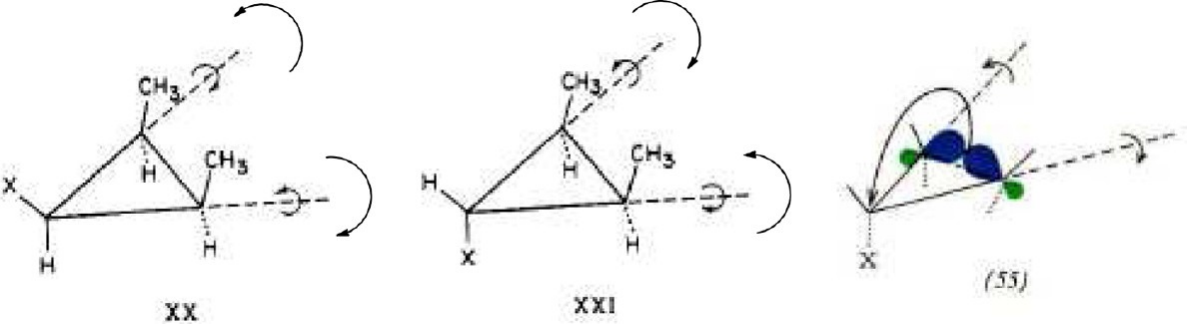
Structures XX and XXI from the First W–H Paper, Illustrating the Two Disrotatory Processes for Each Isomer In structures **XX** and **XXI**, the larger arrows (which were not drawn in the original paper) represent the second (alternative) allowed disrotatory motions. However, these alternative motions lack a “special stereoelectronic factor” which is illustrated in **55** (to the right in this scheme). Structure **55** is taken from the “Long Paper” and illustrates assisted “backside displacement of the leaving group.”^124^

Second, in mid-to-late November 1964, Hoffmann performed only one more set of calculations related to electrocyclizations prior to the submission of *The Stereochemistry of Electrocyclic Reactions* paper. At Natick, DePuy approached Hoffmann and inquired if the solvolyses follow the W–H rules and lead to disrotatory rotation, in appropriately substituted cyclopropyl-X compounds, which of the two disrotatory rotations would be observed? The issue is the tension between three not necessarily reinforcing energetic influences, the conservation of orbital symmetry (a disrotatory ring opening), steric effects if the two methyls rotate toward each other, and anchimeric assistance with “backside displacement of the leaving group [assisted] by the electrons of the backbone σ bond of the cyclopropane ring”.^124^ Hoffmann calculated the ring opening of 2,3-*cis*-dimethylcyclopropyl carbocation with a pyramidal carbon at the place where the X^–^ had left to simulate an early stage of the reaction (see Figure 25A and Scheme 3 for other geometric details). Hoffmann’s conclusions based on the extended Hückel calculations are shown in Figure 25B and are included in the very last paragraph of the first W–H paper.^1^

Without doubt, the chemistry shown in Scheme 3 and Figure 25 was motivated by the input of DePuy.^124−127^ Woodward and Hoffmann stated so in their 1968 *Accounts of Chemical Research* paper^125^ and in their 1969 “Long Paper”.^124^ Hoffmann’s EH calculations and analysis of the ionization of *cis*-2,3-dimethylcyclopropyl-X appears in the first W–H paper.^1^ Nonetheless, DePuy was *not* given any credit in *Stereochemistry of Electrocyclic Reactions* even though this intellectual contribution was significant and was appropriately credited in subsequent papers.^124,125^ Woodward and Hoffmann have been criticized for not providing appropriate credit for contributions to the orbital symmetry research to Corey, as mentioned above, or to others. These serious allegations will be discussed in detail in a subsequent paper.

It is of note that in Hoffmann’s laboratory notebook (see Figure 25A, page 79 of the *Summer* → *November 1964* laboratory notebook), he was drawing the cyclopropyl rings in a professional organic chemist’s fashion, a quasi-realistic sideways perspective rather similar to the way they appear in the final paper (see Scheme 3). This is a subtle indication of Hoffmann’s rapid advance in his knowledge and comfort in organic chemistry. On this same page, Hoffmann drew the Walsh orbitals of cyclopropane. He was very familiar with them, and there are scattered orbital drawings in his notebooks. But this is the first explicit drawing of a molecular orbital, with phases, in the context of electrocyclic reactions since page 80 of the *Early 1964* laboratory notebook and *The Woodward Challenge* (Figure 4).

## VIII Writing the Paper

**Figure 26:**
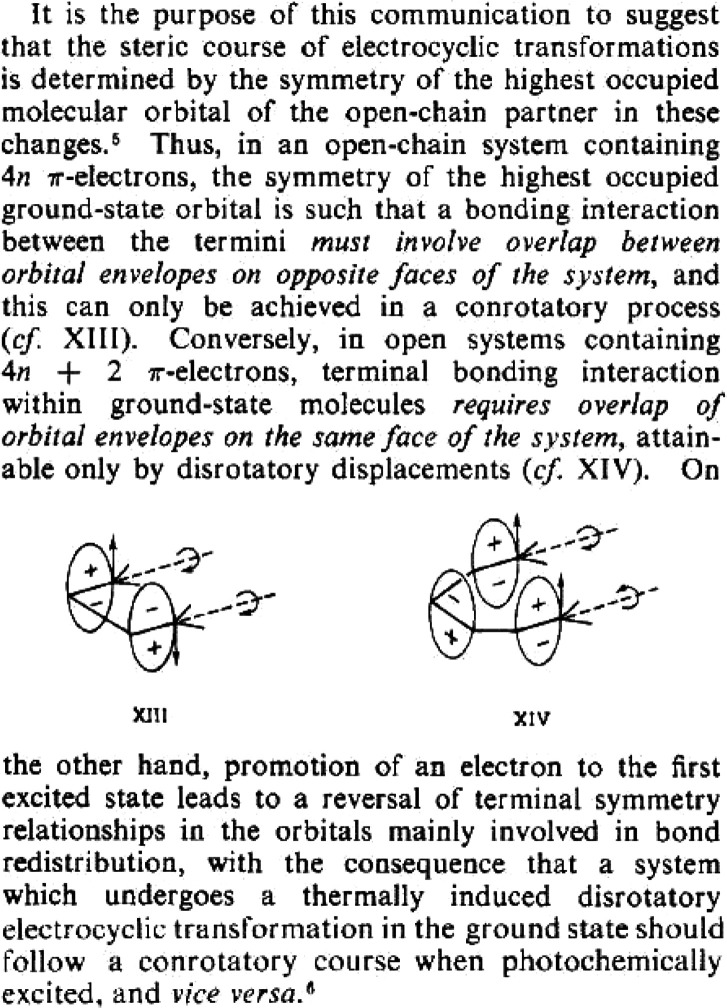
Second paragraph from Woodward and Hoffmann’s *Stereochemistry of Electrocyclic Reactions*.^1^

The word count of *Stereochemistry of Electrocyclic Reactions* exceeded the Journal’s standards for a communication (see Figure 1 which, in Woodward’s own words, acknowledges this fact). Nonetheless, the paper is concise and exquisitely written, as would be expected from a Woodward manuscript. An outline of the paper’s content is as follows:

Topic 1: Several definitions of novel terms (“electrocyclic,” “conrotatory” and “disrotatory”) and a simultaneous description of the reaction types under investigation.
Topic 2: A short but visually and verbally powerful statement of the mechanistic problem including the relevant literature.
Topic 3: A simple yet definitive frontier orbital (almost graphic) explanation and set of predictions for a set of well-known but previously inexplicable, “no-mechanism” reactions (Figure 26). These form the first statement of the Woodward–Hoffmann rules.
Topic 4: A set of boundary conditions outside of which the rules are *not* applicable.
Topic 5: A summary of several extended Hückel calculations that supports the qualitative frontier orbital argument presented in an earlier section.

No new experimental results are included in this paper.

*Stereochemistry of Electrocyclic Reactions* is historically and scientifically important—and led to a Nobel Prize. Its writing style is illustrated by the several excerpts in the figures herein.

**Figure 27:**
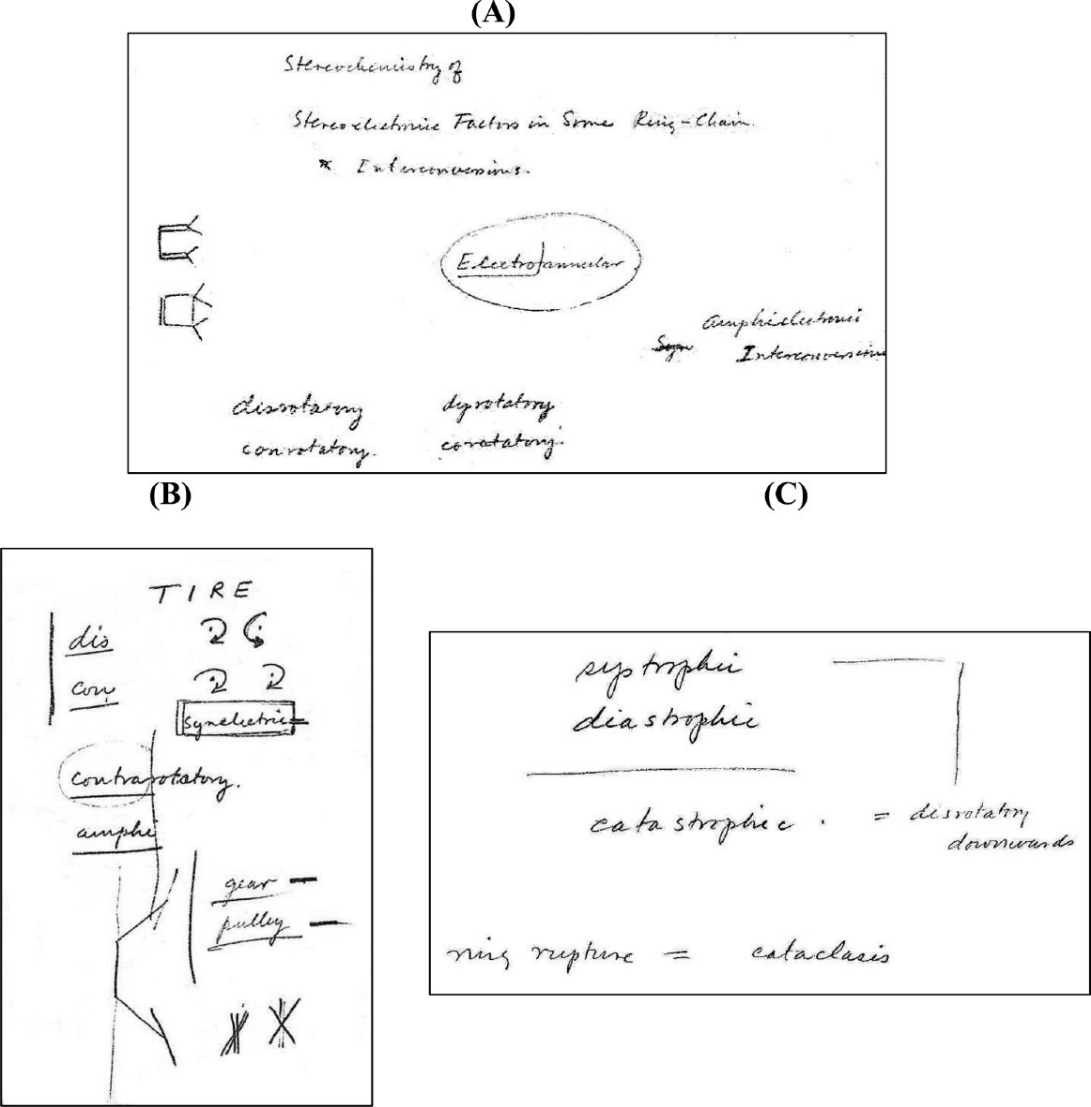
Notes in Woodward’s handwriting from the Woodward archives.^129^ (A) At the top, Woodward proposes a title “Stereoelectronic Factors in Some Ring–Chain Interconversions” as the title for what became *Stereochemistry of Electrocyclic Reactions*. This page may also be the first appearance of “disrotatory” and “conrotatory” along with eventual discards “dyrotatory” and “coratatory” or possibly “corotatory”. Note also “Electro-annular” instead of “electrocyclic” and “amphielectronic interconversions.” (B) “TIRE” may well refer to the four drawings immediately below the four graphics that look like rotating tires. Directly below the rotating tires, in the box, is “synelectric”. To the left are “dis,” “con”, and “conrotatory” followed by “amphi” and “gear” and “pulley”. At the very bottom are graphics that represent the simultaneous rotation about the “termini of a linear system containing *k* π-electrons.”^1^ These graphics likely reflect conversations between Woodward and Hoffmann, as the graphics are identical to those found often in Hoffmann’s notebook (see Figures 11–13 and 17). (C) Woodward appears to have moved past “systrophic” and “diastrophic” because of their similarity to “catastrophic,” as he searches for a “downwards” motion. He then proposes “disrotatory.” At the bottom, he equates “ring rupture” with “cataclasis.” Regarding “catastrophic,” see the text and Figure 28.

Figure 27 contains three notes found in the Woodward archives dealing with his quest for a title and the terms he and Hoffmann would designate for the paired rotations in electrocyclizations. Woodward’s imagination coupled with his bent toward drama, ancient history, and the classic languages led him to invent numerous words, some of which are shown in Figure 27. The derivation of the term “electrocyclization” is straightforward, though it is important to note that concertedness was not stated in W–H Paper 1 as a criterion for electrocyclizations or for the application of the W–H rules to explain the stereospecificities observed. Woodward’s discards–invented words that he rejected–are also of interest.

Note also that the title *Stereoelectronic Factors in Some Ring–Chain Interconversions* was rejected in favor of *Stereochemistry of Electrocyclic Reactions*.

**Figure 28:**
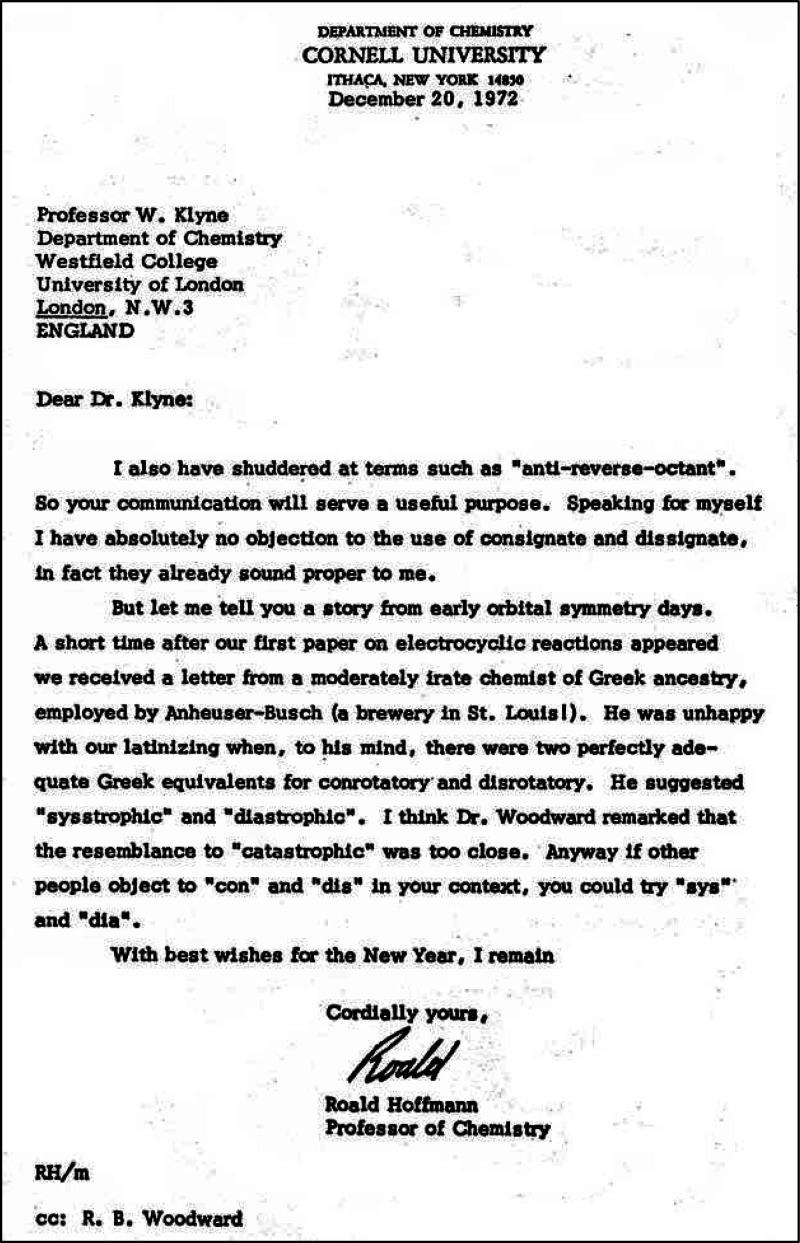
Roald Hoffmann’s response to William Klyne on December 20, 1972, discussing Woodward’s and his choice of the Latin-derived conrotatory and disrotatory rather than the Greek-derived sysstrophic and diastrophic. See also Figure 27C which indicates that Woodward considered these words prior to the drafting of the *Stereochemistry of Electrocyclic Reactions* paper.

There is a light side to the hunt for appropriate nomenclature. In the 1970s, William Klyne of the University of London was on various IUPAC committees on nomenclature. In 1972, Klyne wrote to Woodward and Hoffmann, inquiring about “the [potential] use of consignate and dissignate” in organic chemistry, presumably because these terms were similar to conrotatory and disrotatory. Hoffmann’s response is found in Figure 28 in which he relates a story about a chemist of Greek ancestry who was not satisfied with Woodward and Hoffmann’s choice of the Latin-derived “conrotatory” and “disrotatory” nomenclature. This individual proposed “sysstrophic” and “diastrophic” and Hoffmann tells Klyne,

“I think Dr. Woodward remarked that the resemblance to “catastrophic” was too close.”^128^

Indeed, Figure 27C reveals this very same sentiment, “catastrophic”, in Woodward’s own handwriting! In Woodward’s papers, there is no mention of the “chemist of Greek ancestry”. The evidence shown in Figure 27 suggests that Woodward came up with sys- and diastrophic *prior* to the drafting of the *Stereochemistry of Electrocyclic Reactions* though it is possible that he is recording, in his own notes, a suggestion that was made to him.

Within the Woodward papers at the Harvard University Archives and in Roald Hoffmann’s professional files at Cornell, a number of drafts of their *Stereochemistry of Electrocyclic Reactions* paper are available. Two of these are handwritten drafts by Woodward. One is a typewritten draft by Hoffmann, though there are several versions of this draft with handwritten modifications and cross outs, in both Woodward’s and Hoffmann’s hands.

There are also a number of unattached pages composed of handwritten text by Hoffmann that appear to be notes or possibly, though unlabeled, inserts for one of these drafts.

Taken all together, there is a remarkable feature of the published paper *Stereochemistry of Electrocyclic Reactions* that cannot be recognized unless and until one examines the initial drafts of the paper (or reads this paper). *Stereochemistry of Electrocyclic Reactions* is actually two nearly independent mini-papers pasted together: Woodward’s frontier orbital explanation, with phases and nodes explaining bonding or antibonding overlaps (topics 1–4 in the list above), and Hoffmann’s extended Hückel calculations that confirm the qualitative frontier orbital explanations (topic 5). Woodward’s two existent drafts begin almost identically:

“Sir: We define as electrocyclic transformations the conversion of an open-chain substance...”

and chronologically, the next,

“Sir: We define as electrocyclic transformations the formation of a single bond between...”

Woodward’s final draft forms essentially the first half of *Stereochemistry of Electrocyclic Reactions*. Hoffmann’s draft begins,

The stereochemistry of the ring-opening or cyclization reaction was studied theoretically by means of the extended Hückel theory...

and is essentially the second half of Stereochemistry of Electrocyclic Reactions.

*Stereochemistry of Electrocyclic Reactions* was written as if the two coauthors were collaborators in name only rather than two scientists who have worked closely together, with frequent interactions. That is, in fact, how the research for the first paper was conducted and the how the first paper was written. Hoffmann does not remember but thinks there must have been meetings from May through November 1964 with Woodward to discuss his (Hoffmann’s) extended Hückel calculations. In addition, there is no evidence of memos written by one to the other during this time within either the Woodward or Hoffmann papers, nor are there any notes within Hoffmann’s notebooks^130,131^ other than on page 80 of the *Early 1964* laboratory notebook dated May 5, 1964 (Figure 4), that includes Woodward’s name. Nor does Hoffmann remember any details of meetings to discuss the drafts of their first paper.

Hoffmann muses about the nature of his collaboration and interactions with Woodward:

“Let us assume that Woodward was unsure of himself, of the strength of his frontier orbital explanation. He wanted computational support, for which he turns to me. I am a junior collaborator. He asks me to provide a draft of the computational results. I do just that. I am not strong enough yet, my knowledge, in appreciation of the power of the frontier orbital argument, in my position at 27 years old relative to RBW, to draft the whole paper. By the time of the second paper, just four-five months later, I am strong enough.”^80^

Hoffmann’s assessment of Woodward’s motivations for eliciting his participation was confirmed by Woodward, himself, in his 1973 Cope Award address.^51^ Woodward said,

“After a brief false start in extending these ideas, attributable clearly to my gaucherie in the details of quantum chemistry, I very soon realized that I needed more help than was available in my immediate circle, and I sought out Roald Hoffmann, who was well-known to me, though by reputation only, as a brilliant young theoretician. . . I told him my story, and then, essentially, put to him the question, ‘Can you make this respectable in more sophisticated theoretical terms?’ He could, and did...”^51^

It is not unusual practice in science—it may even be the standard experience—that in collaborations among scientists of distinctly different disciplines, papers are prepared by pasting together various sections written by the individual collaborators with minimal interaction among them. This appears to have been the process for the first Woodward–Hoffmann paper, *Stereochemistry of Electrocyclic Reactions*. But over the next nine years, until their final paper on the Rules of *Conservation of Orbital Symmetry*,^132^ the mode of interaction and collaboration between Woodward and Hoffmann evolved significantly. This evolution will be discussed in a future paper.

**Figure 29:**
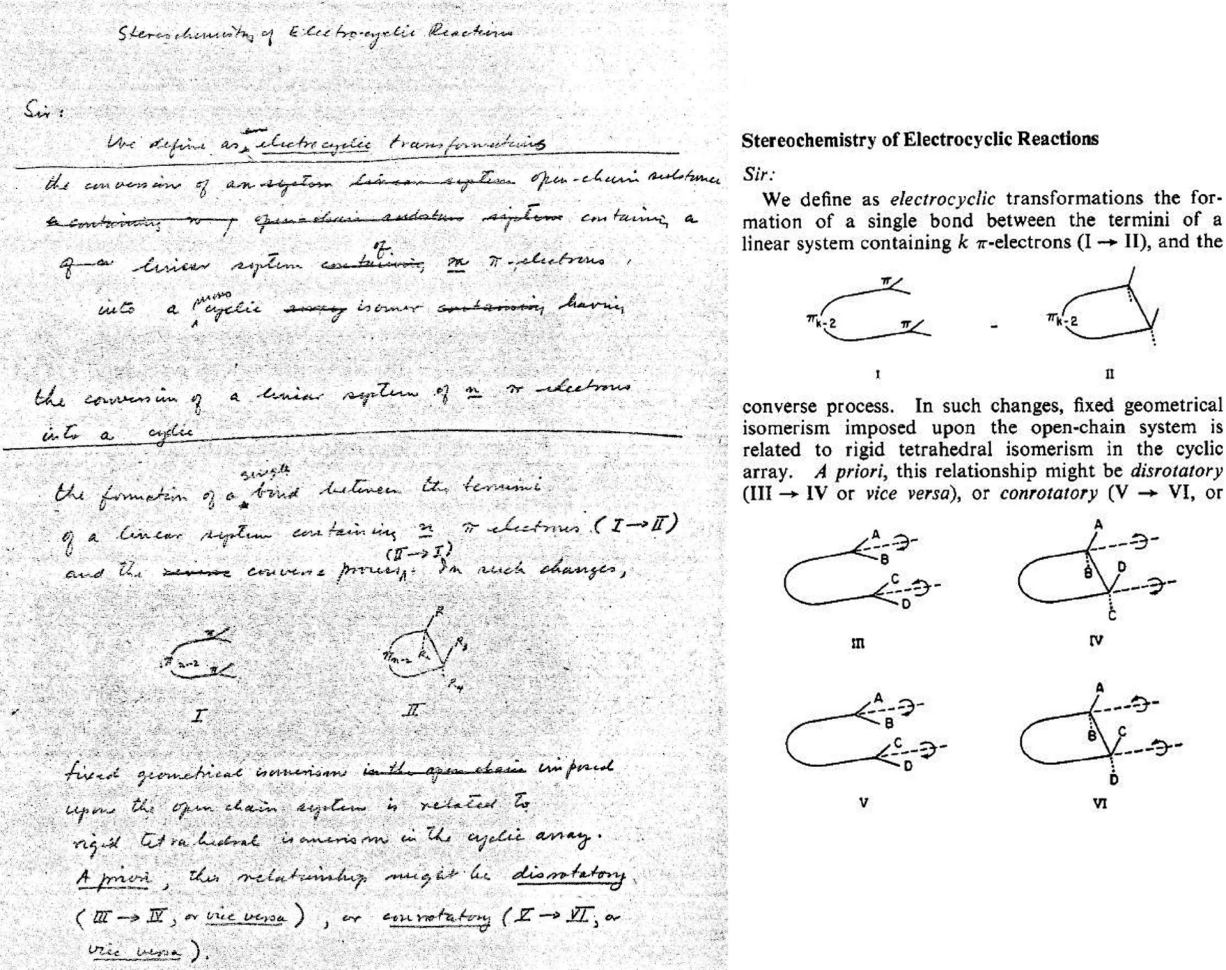
(Left) First page of first handwritten draft by R. B. Woodward of the *Stereochemistry of Electrocyclic Reactions* paper.^139^ (Right) First paragraph of *Stereochemistry of Electrocyclic Reactions*.

**Figure 30:**
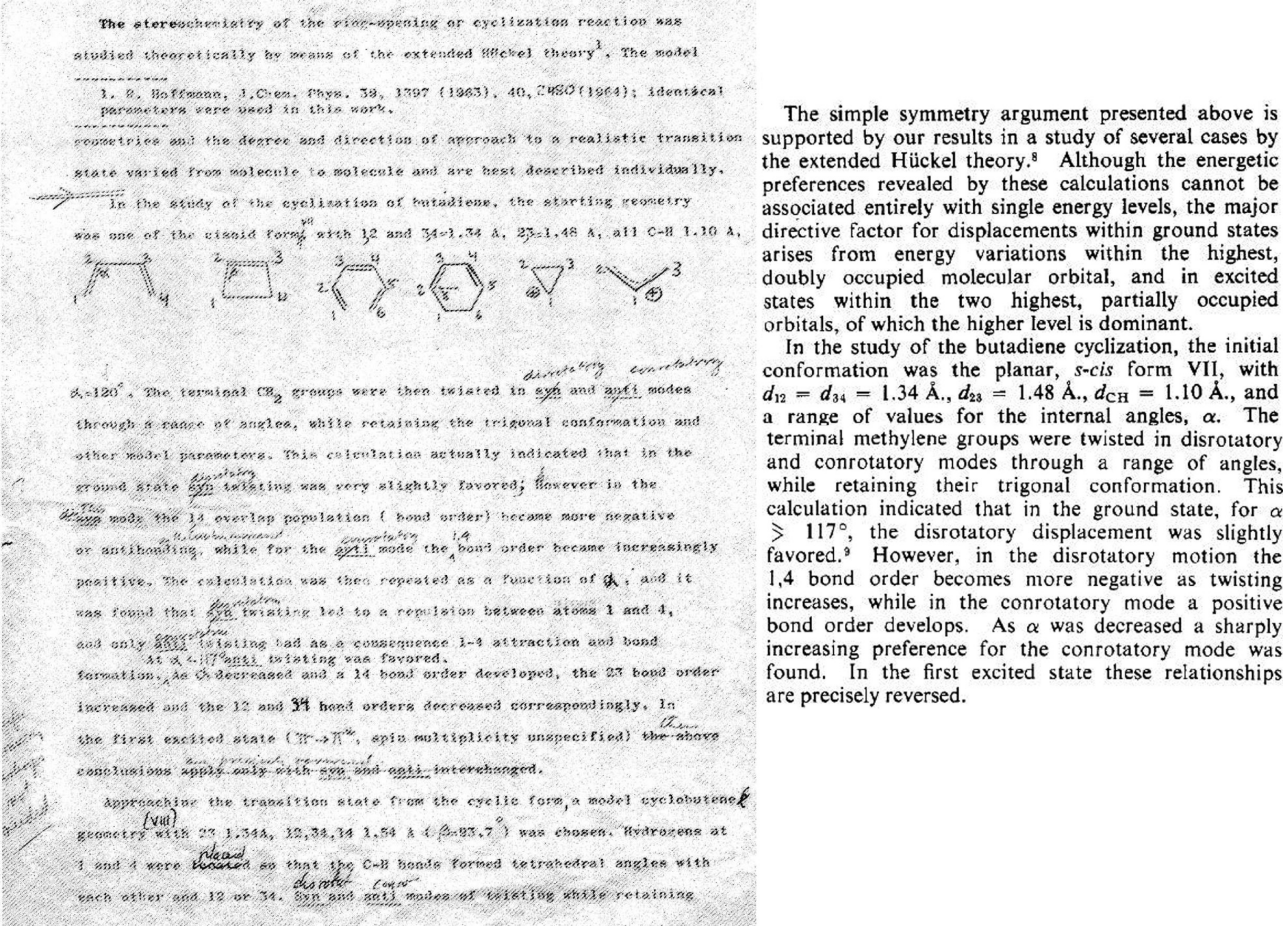
(Left) First typewritten draft by R. Hoffmann of his portion of the *Stereochemistry of Electrocyclic Reactions* paper.^140^ Handwritten modifications by both Woodward (lighter) and Hoffmann (darker) appear. Note both Woodward’s and Hoffmann’s crossing out of “anti” and “syn” and replacement of those terms with “controtatory” and “disrotatory,” respectively. (Right) Corresponding text from *Stereochemistry of Electrocyclic Reactions*.

It is also beyond the scope of this paper to examine in detail the slight corrections and modifications made by Woodward and Hoffmann, from draft to draft. But it is pedagogical and even amusing to see the first page of both Woodward’s and Hoffmann’s first drafts (Figure 29 and Figure 30, respectively). From this author’s many weeks of time within the Woodward papers at Harvard, it is evident that Woodward prepared many of his first drafts in pencil, going back to his formal total synthesis of quinine^11,12^ and his reports to the federal government on the structure of penicillin during World War II. Generally, those first drafts are almost identical to the published paper, and indeed this is the case in this instance. Woodward’s hand drawn chemical structures and graphics were always carefully drawn, often using straight edges. His handwriting was small but nearly always readable. There is a simple beauty in his hand. Cross-outs and revisions are frequent but the meanings are unambiguous.

Hoffmann’s typewritten first draft is shown in Figure 30 absent some redundancies and computational and molecular orbital energy details. Hoffmann used the terms “syn” and “anti” in his draft. These words are crossed out and replaced, in both Woodward’s and Hoffmann’s handwriting, with “disrotatory” and “conrotatory”, respectively. This is a rather remarkable set of modifications, the implications easily overlooked. Consider that “syn” typically refers to the same side or direction and “anti”, the reverse. Hence, “syn” ought to have been replaced by “con” but it was replaced by “dis”. Hoffmann recently explained this as follows:

“When I assigned the names syn and anti to the motions [in my notebook], I didn’t think of looking at whether the directions of the rotational arrows were in the same sense (say clockwise or counterclockwise) or opposite sense. I was just thinking of the substituents on the inside (or the heads of the rotational arrows) going in the same direction (both up, both down) or opposite (one up, one down; that's how I put in the coordinates in the EH computations). On the same side I called syn = disrotatory then. Weird choice, I admit.”^133^

The timing of Woodward’s and Hoffmann’s individual first drafts is unknown. It is not until a later draft that these two mini-drafts were pasted together into a single manuscript. This process of preparing *Stereochemistry of Electrocyclic Reactions* is further evidence of an initially rather isolated collaboration between Woodward and Hoffmann. As discussed briefly above and will be discussed in more detail in a subsequent publication, the Woodward–Hoffmann collaboration evolved with time as the two scientists developed expertise in the other’s discipline and as Hoffmann transformed from a calculator to a full-fledged collaborator.

**Figure 31:**
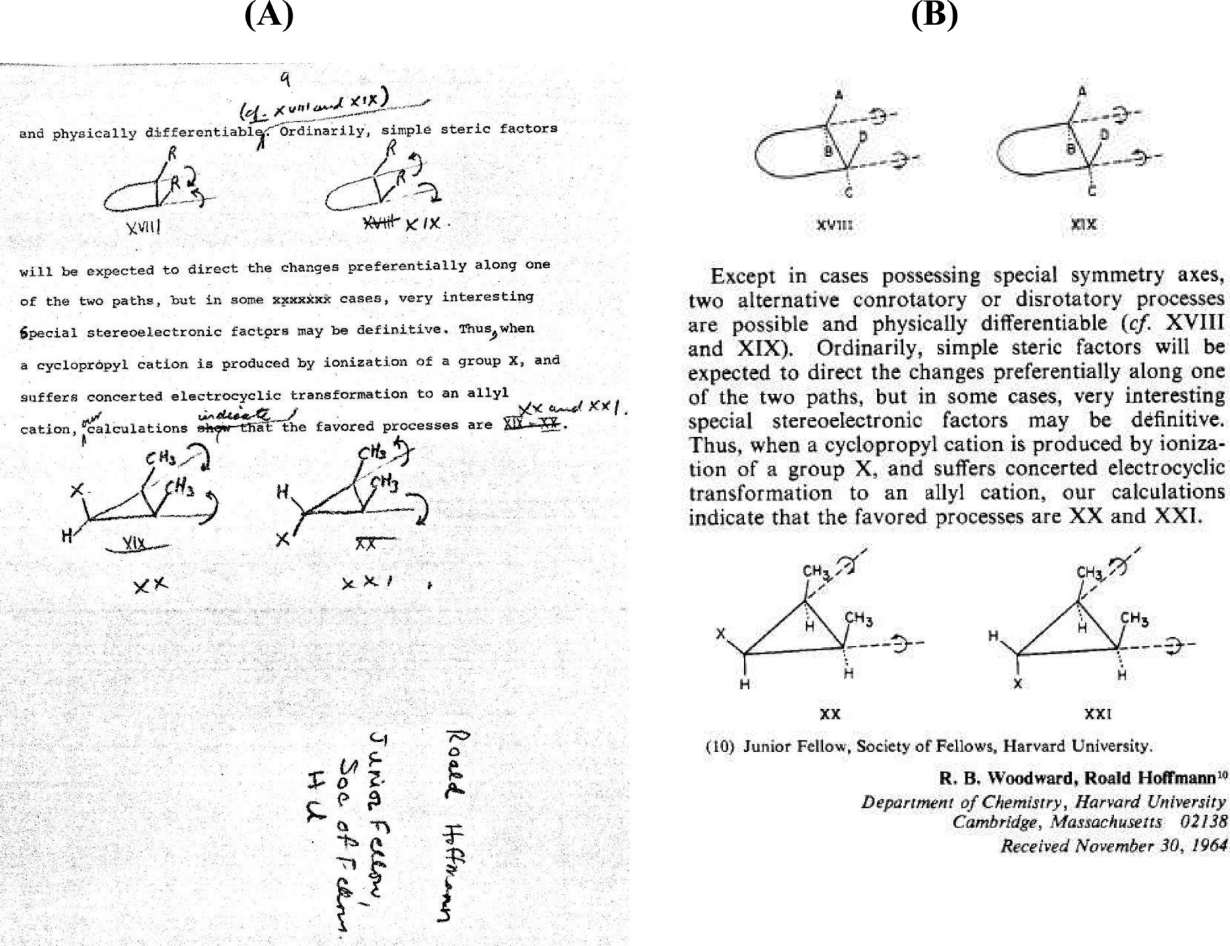
(A) One page from a near final draft of *Stereochemistry of Electrocyclic Reactions*. The structures were written by Hoffmann, the changes in the text by Woodward, and the name “Roald Hoffman” [sic] and the address, where HU refers to Harvard University, is presumably by Dolores Dyer, Woodward’s assistant.^141^ Note the initial misspelling of Hoffmann’s name; careful inspection of the figure shows that the second “n” was added subsequent to the initial notation. (B) The last paragraph from the first W–H paper.^1^

In the examples and discussions above, focus was on the direction of rotation of atoms during an electrocyclic reaction, conrotatory versus disrotatory, depending on the number of electrons and whether the reaction occurs in the ground or first excited state. But there is yet another stereochemical feature: depending on the compounds involved, there can be two conrotatory (or two disrotatory) motions. As discussed above, the idea that under some circumstances there could be an electronic distinction between the two allowed disrotatory motions was first pointed out to Hoffmann by DePuy at the Natick Conference^126^ and subsequently credited to DePuy.^124,125^ Hoffmann’s recordings of his calculations and analyses are shown in Figure 25, and an early draft of these results is in Figure 31. It is amusing to note that Roald Hoffmann was still largely an unknown in Woodward’s ensemble at the time of the drafting of *Stereochemistry of Electrocyclic Reactions*, evidenced by the misspelling of Hoffmann’s name in what were to be the authors’ names and addresses of the publication (i.e., the second “n” in Hoffmann in Figure 31A was added subsequently, likely by Woodward’s assistant, Dolores Dyer).

There is a special flair to Woodward’s prose, a factor immediately evident to any chemist. Hoffmann was eventually to develop a distinctive style of writing as well, and indeed he has devoted much energy to poetry, playwriting, and other prose. (For a listing of Hoffmann’s literary works, see his personal webite, www.roaldhoffmann.com.) But there is no hint of that in Hoffmann’s workmanlike computational summary that formed the second half of *Stereochemistry of Electrocyclic Reactions*. Hoffmann recently reflected,

“To me, the interesting question is why Woodward, very much the senior author, conscious and aware of the importance of style in writing, the master of flowing chemical prose, does not take my “workmanlike” calculator’s prose and rewrite it? I would not have objected.”^134^

Woodward, his writing style, and his acceptance of his colleagues’ writing styles will be the subject of a future publication in this author’s series on the “Words” of eminent chemists (see Stork’s words,^135^ Djerassi’s words,^136^ and Roberts’s words.^137^).

**Figure 32:**
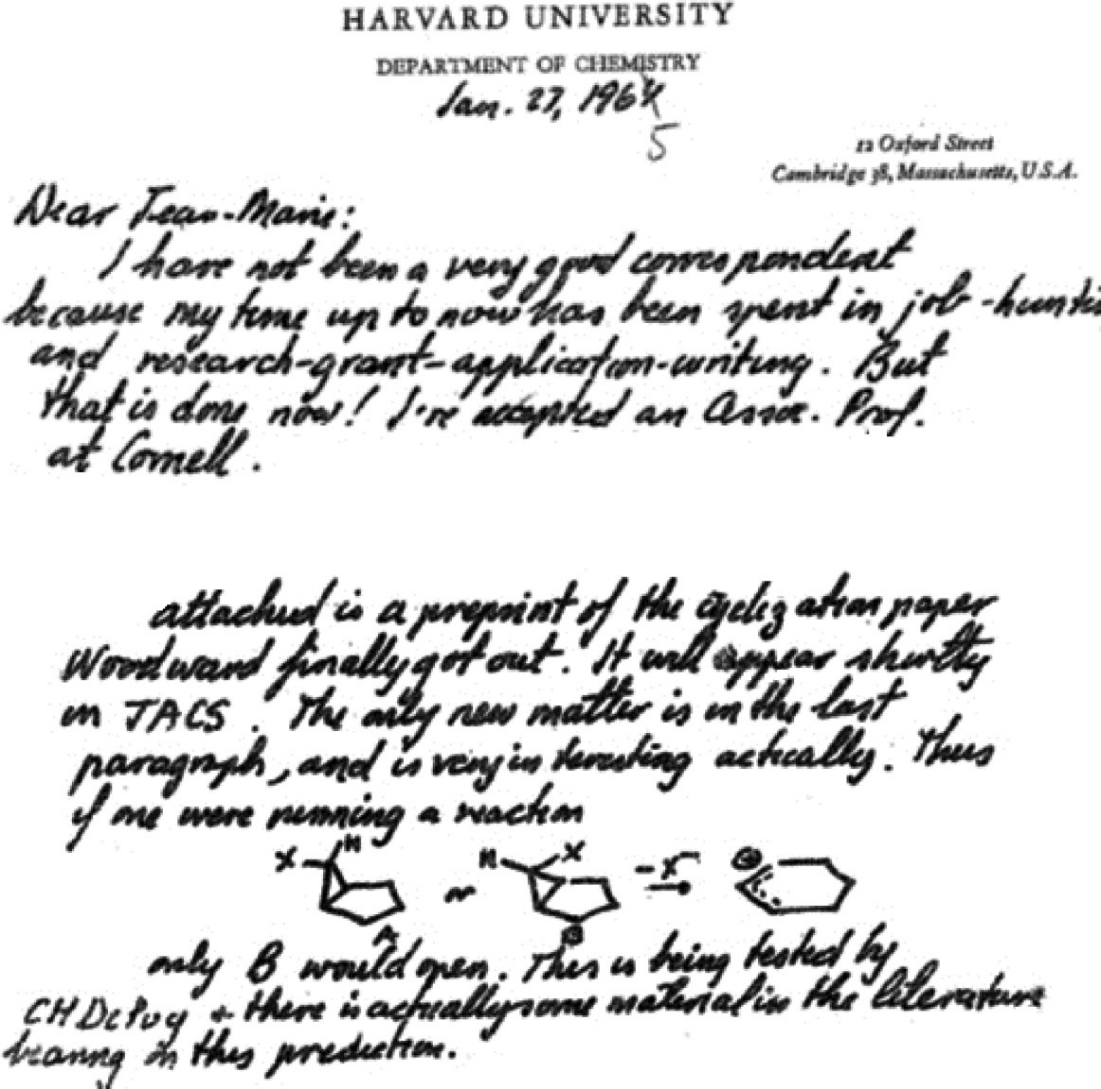
Two excerpts from a letter from Hoffmann to Jean-Marie Lehn, a former postdoctoral student of Woodward’s when Hoffmann was a Junior Fellow at Harvard.^138^ In this letter written January 27, 1965, Hoffmann informs Lehn that he accepted a position at Cornell, that the first Woodward–Hoffmann paper was submitted (“Woodward finally got it out”), and that some new and exciting chemistry was discovered.

On January 27, 1965, two months after the submission of the manuscript and several weeks following its publication, Hoffmann wrote to his friend and subsequent Nobelist Jean-Marie Lehn (Figure 32). In this handwritten letter dated “1964” but clearly written in 1965, Hoffmann apologized for his tardiness in writing and reported that he had accepted a position as an Associate Professor at Cornell. Hoffmann attached a preprint of *Stereochemistry of Electrocyclic Reactions* and stated,

“attached is a preprint of the cyclization paper Woodward finally got out. It will appear shortly in JACS. The only new matter is in the last paragraph, and is very interesting actually. . . ”^138^

Hoffmann is referring to the stereochemical ring opening dis-opening of the cyclopropyl-X chemistry and cites the involvement of DePuy. But it is the “Woodward finally got it out” that suggests that there was a tardiness in publication due to Woodward. Indeed, Woodward was well-known to be tardy in his publication record.^33^

Hoffmann proposed an alternative explanation for his words to Lehn:

“Again, you are assuming that human beings, I here, are talking like legal documents. I may have just been speaking colloquially. It could have meant ‘we finally got it sent out,’ and the Woodward reference may be just acknowledgment of who was in charge.”^80^

How very interesting it is, for those interested in the history of a subject, not to be completely free to read the documents of the past literally and to interpret these documents exactly as they are written. But then, is that asking more of the archives than we expect in our daily intercourse with our fellow chemists, with our fellow human beings?

## IX Next Steps: Hints at Further Advances in the Conservation of Orbital Symmetry

*The Woodward Challenge* (Figure 4) included several examples of what came to be termed “electrocyclic reactions” and would serve as the focus for Hoffmann’s initial research in the field. Indeed, the May 5 meeting may have been the sole interaction between Woodward and Hoffmann from until mid- or even late-October 1964. There is no written evidence nor “precise memory”^85^ on Hoffmann’s part that Woodward discussed “cycloadditions” (or “cycloreversions”) or “sigmatropic rearrangements” with Hoffmann on May 5, 1964. Nor is there any evidence within Hoffmann’s laboratory notebooks between May and November 1964 of a discussion between these two collaborators on the possible extension of their research into these other reactions.^142^ However, within the Hoffmann laboratory notebooks of 1964, there are several forerunners to what would appear in the second and future papers by Woodward and Hoffmann.

First, some background. Hoffmann was surely *not* living in a state of isolation from the literature of organic chemistry from May through November 1964. As stated above, he was taking a course on small ring organic compounds taught by Applequist. He was reading the organic chemical literature (see Table 2 for an indication of his specific interests). He attended three international organic chemical meetings, in Oregon, Strasbourg, and Massachusetts, during that time (Tables 1 and 2). He was having extensive discussions with Corey, Jean-Marie Lehn, Subramania Ranganathan, and other colleagues and fellow students at Harvard. He was networking in many other ways, for example, exchanging letters with academic chemists and participating in the process of his academic job search.

**Figure 33:**
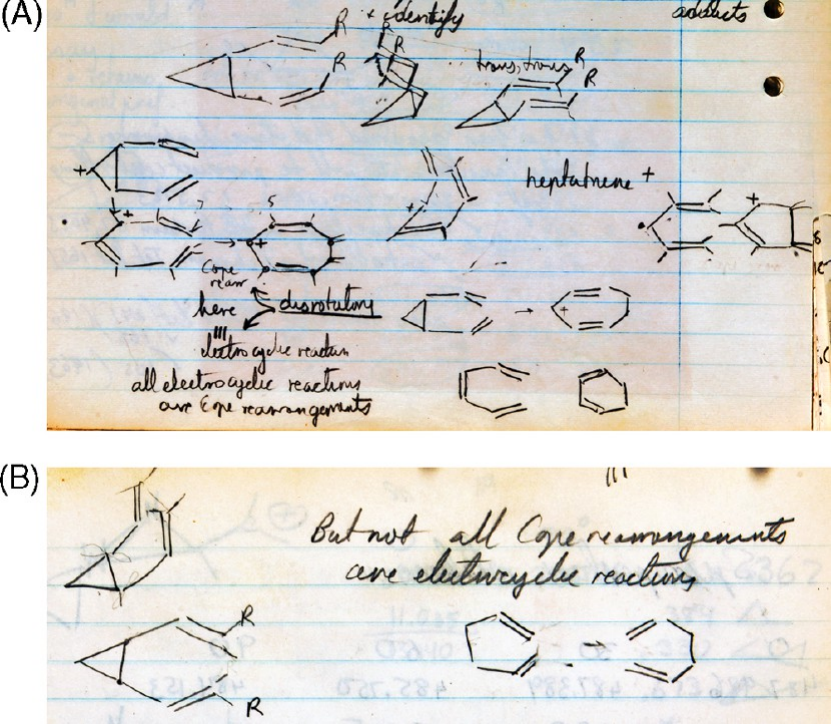
Excerpts from Hoffmann’s laboratory notebook *Summer → Nov 64,* written approximately November 20, 1964. (A) From the bottom of page 116. (B) From the top of page 117. Note the use of the terms “electrocyclization” and “disrotatory,” placing this page chronologically after the first combined draft of the *Stereochemistry of Electrocyclic Reactions*.” There is also recognition of the extension to cycloadditions (Cope rearrangements).

It is not surprising, therefore, that there are a number of notes and literature examples within Hoffmann’s papers and laboratory notebooks of his connecting his research on electrocyclizations to what would later be called cycloadditions and sigmatropic rearrangements. One example of this is shown in Figure 33, dealing with with sigmatropic rearrangements, an example of which is the Cope rearrangement which is a symmetry-allowed [3,3] sigmatropic rearrangement.^142^ In Figure 33, Hoffmann refers to a Cope rearrangement as “disrotatory...Cope rear[angement] here ≡ [““is something like” or “could be viewed like””^80^ an] electrocyclic reaction”. Here, Hoffmann is observing that the Cope rearrangement could be viewed like an electrocyclic reaction, thereby conceptually tying these seemingly different reaction types together.

Of note is that in 1973, less than ten years later, Woodward and Hoffmann would be the first recipients of the Arthur C. Cope Award (Figure 6), one of organic chemistry’s most prestigious awards. Given that the Cope rearrangement was an important example in Hoffmann’s early work and in Woodward and Hoffmann’s sigmatropic rearrangements, their receipt of the Cope Award was most fitting. (In contrast, the Cope rearrangement was *not* one of Woodward’s mysterious reactions though it was a favorite research topic^9,143^ of Doering, the inventor of the term “no-mechanism reactions.”^9,10^)

On page 26 of the *Summer* → *Nov 1964* notebook, Hoffmann discusses the stereochemistry of the Diels–Alder reaction, a 4 + 2 cycloaddition. No connection is made between the Diels–Alder reaction and either electrocyclizations or orbital symmetry considerations. However, some months earlier, on page 123 of the *Early 1964* notebook, Hoffmann draws the transition state of the Diels–Alder reaction of 1,3-butadiene and ethylene. Hoffmann writes that the ∠C=CC in 1,3-butadiene “doesn’t have to contract to form an intermolecular bond. It can stay large.” Hoffmann reconstructs his thoughts,

“I worry that the CCC angle is important, just as for electrocyclic reactions. . . a connection [with electrocyclic reactions] was made, I think, but not in a very productive way.”^144^

## X What Else Were Woodward and Hoffmann Doing in 1964?

It is easy and quite reasonable to imagine that Woodward and Hoffmann were focused, if not entirely, at least substantially—on what was to become a major chemical breakthrough, a substantial topic in every organic chemistry undergraduate textbook, and research recognized by numerous awards including the 1981 Nobel Prize. But as the above discussion has revealed, both Woodward and Hoffmann were really doing many other things.

The year 1964 was busy for Woodward. He was at the height of his powers, just a year from receipt of his Nobel Prize. He wrote and submitted a number of substantial papers in the time period under consideration herein (Table 4). In addition to leading a very large research group at Harvard, the Woodward Research Institute had just opened in Basel, Switzerland.^89^ Thus, in addition to Woodward’s massive travel schedule that often included Switzerland, he now had scientific leadership responsibilities in Basel which he took quite seriously. Of the many research programs he supervised, perhaps the most challenging—scientifically and emotionally—was the total synthesis of vitamin B_12_.^145−148^ While his research on vitamin B_12_ began ca. 1960–1961, the famous Woodward–Eschenmoser Harvard–Eidgenössische Technische Hochschule collaboration was being initiated in the 1964–1965 time period.^44^ Indeed, it was Ranganathan’s unanticipated experimental results dealing with a 1,3,5-hexatriene ⇌ 1,3-cyclohexadiene electrocyclization as a key step in Woodward’s vitamin B_12_ synthesis that added to his four mysterious reactions as a source of major puzzlement and anxiety (Figure 5).^51,149^

The year 1964 was busy for Hoffmann, too. This was the middle year of his Harvard Junior Fellowship, and he was making full use of his newly developed extended Hückel theory as a computational tool to predict and explain much of organic chemistry (Tables 1 and 2). Hoffmann was also learning a lot of chemistry, attending international meetings and networking, and applying for an academic position. He was also publishing nearly all his calculations though with some delay.

A noteworthy, almost contradictory, phenomenon occurred in Hoffmann’s scientific professional trajectory. As illustrated in Tables 1 and 2, he was performing extended Hückel calculations on a wide assortment of structures and functional groups. As noted above, Hoffmann’s lifetime record from the early 1960s was to publish many papers in a continuous stream. However, the year 1964 was different. In the months prior to his May 5, 1964, meeting with Woodward (Figure 4) and continuing well into 1966, Hoffmann was seemingly calculating everything under the sun. But excluding his five W–H rules papers, i.e., the conservation of orbital symmetry papers with Woodward, Hoffmann did not submit a paper from January 30, 1964 until July 6, 1965—over 17 months (Table 5). That would imply that most of his research was on the conservation of orbital symmetry project. But, as discussed in detail above and illustrated in Tables 1 and 2, during this time, Hoffmann hardly worked on *The Woodward Challenge.*

But during that time, he was not *publishing* much of his other work! In fact, from January 30, 1964, until July 6, 1965, the only papers submitted were the five Woodward–Hoffmann communications to the *Journal of the American Chemical Society*.^1,150−153^ Hoffmann was playing intellectually, enjoying himself, performing extended Hückel calculations on hundreds of compounds in their ground and various excited states, unaware that *The Woodward Challenge* was going to be a major breakthrough in organic chemistry and a personal life-changing experience. He also needed to find a job (his interviews for academic positions took place in October through December 1964). He and his wife were beginning a family—their first child was born in February 1963, their second in May 1965. As Hoffmann summarizes,

“The pages [of my laboratory notebooks] demonstrate that, at the time, I did not think the [Woodward] project was all that important. I was off to other parts of organic chemistry: cyclopropanes, carbonium ions, aldehydes and ketones.”^81^

But what Hoffmann did calculate, he eventually published; indeed, he published nearly every calculation he performed in this time period.

Just as the extended Hückel calculations that modeled electrocyclic reactions were typically comparisons of single-point (MO) energies for different reaction trajectories, the EH calculations on various organic substances (Tables 1 and 2) were not extensive computations, e.g., complete energy surfaces, as would be performed several decades later. But these calculations did serve as the basis for many Hoffmann publications in the mid-to-late 1960s and well into the 1970s (see Table 5).

**Table 5:** Partial Publication Record of Roald Hoffmann with Emphasis on Orbital Symmetry Publications (OS) and the Time-Frame during Their Publication

paper number^s^	year published	topic	journal	submission date
11^s^	1963	EHT-I – hydrocarbons	*JCP*	Apr 10, 1963
12^s^	1964	boron–nitrogen molecules	ACS Books	Jan 1, 1963
13^s^	1964	electronic levels in alkanes	*JCP*	Nov 29, 1963
14^s^	1964	nonclassical carbonium ions	*JACS*	Dec 28, 1963
15^s^	1964	EHT-II – azines	*JCP*	Jan 30, 1964
16^s^	1964	EHT-III – boron–nitrogen molecules	*JCP*	Sep 27, 1963
17^s^	1964	EHT-IV carbonium ions	*JCP*	Sep 27, 1963
18	1965	OS – electrocyclic reactions	*JACS*	Nov 30, 1964
19	1965	OS – cycloadditions	*JACS*	Mar 27, 1965
20	1965	OS – sigmatropic reactions	*JACS*	Apr 30, 1965
21	1965	OS – exo and Endo effects	*JACS*	Aug 16, 1965
22	1965	OS – orientational effects	*JACS*	Aug 16, 1965
23^s^	1965	cyclopropane	*TL*	Aug 21, 1965
24^s^	1966	EHT-V cumulenes, polyenes, polyacetylenes and Cn	*Tetrahedron*	Aug 4, 1965
25^s^	1966	EHT-VI diazirines and diazomethanes	*Tetrahedron*	Jul 6, 1965
26	1966	conformational and ssomer stability on the number of electrons in extended p-systems	*JACS*	Sep 21, 1965
27^s^	1966	Electronic structure in TS and reactions	*Trans NY Acad Sci*	Jan 1, 1966
28	1967	spirarenes	*JACS*	May 23, 1967
29	1968	OS – conservation of orbital symmetry	*Acc. Chem. Res.*	Sep 3, 1967
30^s^	1968	trimethylene and CH_2_: + ethylene	*JACS*	May 31, 1967
31	1968	methylenes	*JACS*	May 31, 1967
32	1968	benzynes	*JACS*	May 31, 1967
33	1968	pyridine and H-bonds	*JACS*	Jun 9, 1967
34	1968	isocyanide–cyanide	*JACS*	Jan 17, 1968
35	1968	6 valence electron, 3-centered bonding	*Tetrahedron*	Apr 16, 1968
36	1968	biphenyl, fulvalene	*JACS*	Apr 3, 1968
37	1968	singlet methylene (CH_2_)	*JACS*	Jan 17, 1968
38^s^	1968	H_2_ + I_2_ exchange	*JCP*	Mar 18, 1968
39^s^	1969	symmetry/diradicals	*CC*	Dec 18, 1968
40	1969	formal diradicals/single GS	*AC*	Jan 27, 1969
41	1969	conformational analysis macro-molecules	*Biopolymers*	Aug 15, 1968
42	1969	hetarynes	*JACS*	Oct 22, 1968
43	1969	cyclobutadienes	*Roum. de Chim*	Jan 13, 1969
44	1969	*o*-, *m*-, and *p*-benzynes (CNDO)	*Roum. de Chim*	Feb 2, 1969
45	1969	phenyl-substituted cations, radicals, anions	*JPC*	Dec 9, 1968
46	1969	OS – conservation of orbital symmetry	*AC* and book	Jan 1, 1969

s Single author.

To get a sense as to Hoffmann’s other computational chemistry of the period, it is interesting to compare several pages from his laboratory notebooks with the results reported in his papers. The examples shown here involve calculations performed *after* the meeting with Woodward on May 5, 1964, and *before* the submission of *Stereochemistry of Electrocyclic Reactions* on November 30, 1964.

**Figure 34:**
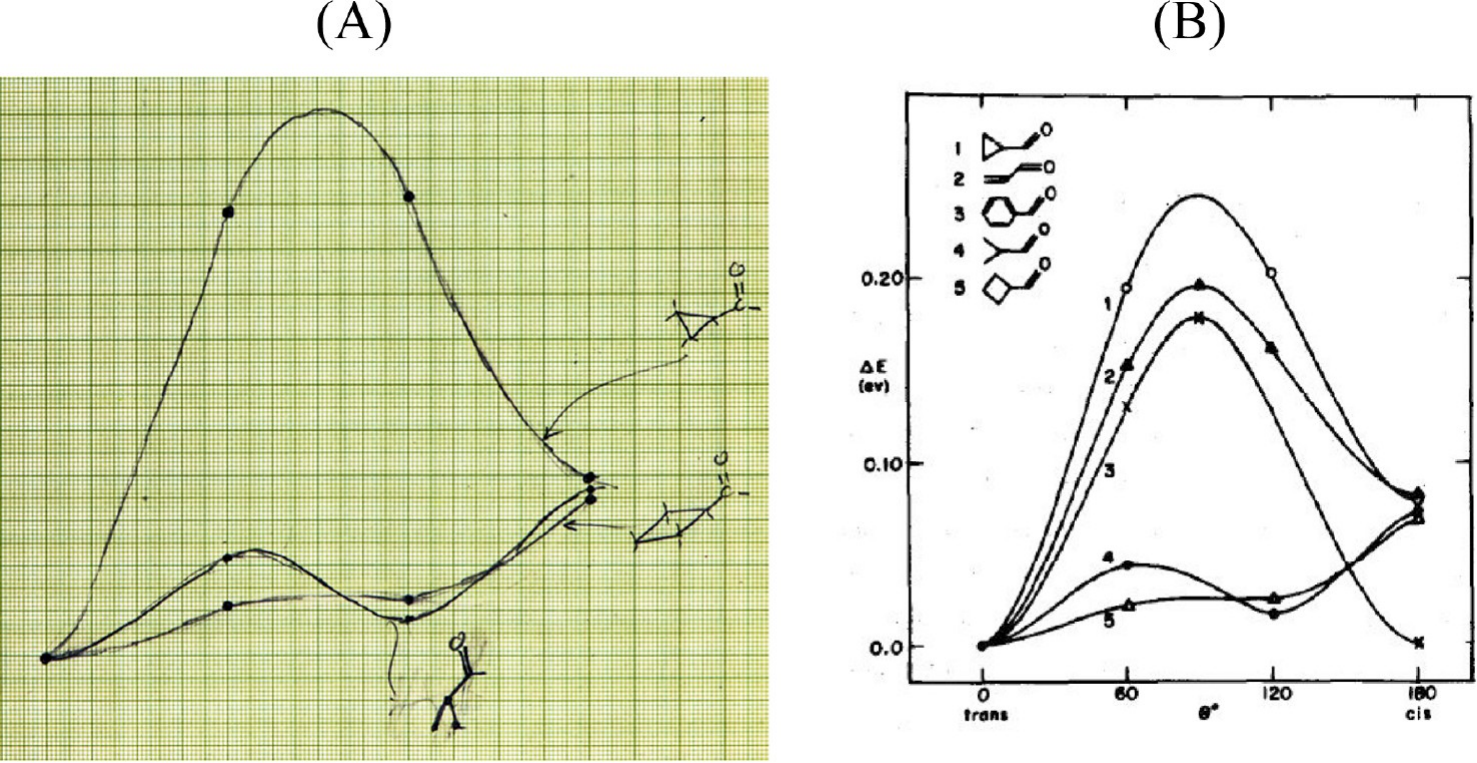
Comparison of (A) page 112 from Roald Hoffmann’s *Early 1964* laboratory notebook with (B) his subsequent publication in *Tetrahedron Letters*(154) in which he reported the potential energy curves for rotation in several aldehydes about the torsion angle O–C–C–C. Only PE curves for compounds **1**, **4** and **5** shown in (B) are found in (A).

On August 21, 1965, Hoffmann submitted a paper to *Tetrahedron Letters* entitled *Some Theoretical Observations on Cyclopropane*. Figure 34 shows Hoffmann’s graph from page 112 of his *Early 1964* laboratory notebook, 32 pages after his record of his meeting with Woodward (page 80, Figure 4), next to the representative graph in Hoffmann’s published paper. In this study, the “relative conjugating ability of cyclopropane [was examined by comparing] the potential energy for twisting around the single bond in R–CHO...for cyclopropyl, vinyl, phenyl, isopropyl, and cyclobutyl. The relative ability of cyclopropane to interact with cation centers...and the excited states of cyclopropanes and spiropentane” were also computationally examined.

In 2015, when reviewing his research performed in 1964 with the author, Hoffmann mused about his focus on MO energies rather than orbitals and the frontier orbital approach in both his early electrocyclization research as well as his other research during this time period. As discussed above, it was Woodward who focused on frontier orbitals in the electrocyclization research.

“In this Tetrahedron Letters paper [Figure 34], I calculate energies but there are no orbitals. I could have been talking about orbitals. There are no orbitals in any of the earlier papers.”^34^

**Figure 35:**
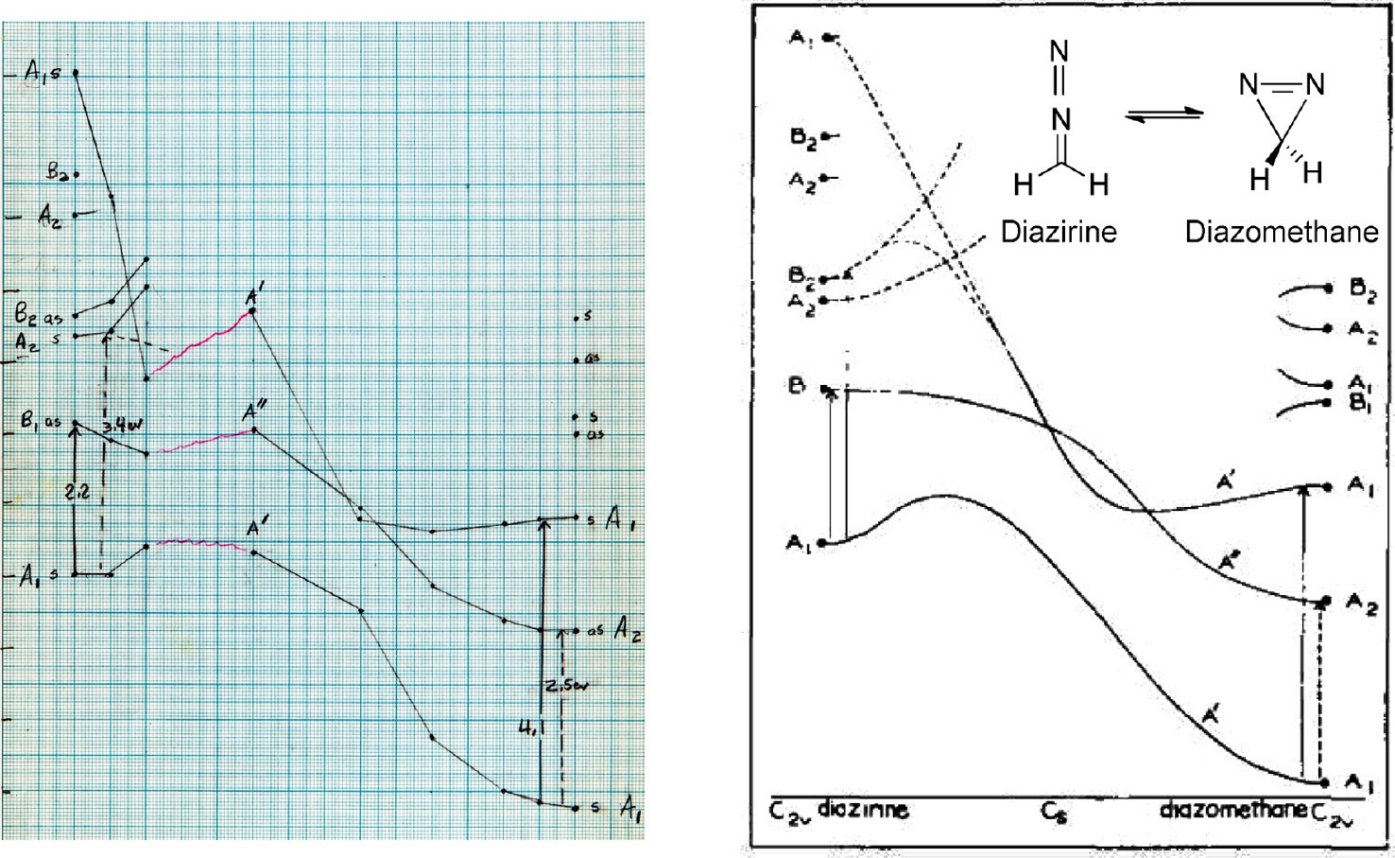
Comparison of page 61 from Roald Hoffmann’s *Summer 1964→ Nov 1964* laboratory notebook with his subsequent publication^155^ in which he reported the “approximate behavior of ground and excited states in the diazirine–diazomethane isomerization”.

On July 6, 1965, Hoffmann submitted a paper to *Tetrahedron* entitled *Extended Hückel theory – VI. Excited States and Photochemistry of Diazirines and Diazomethanes.*Figure 35 compares a set of EH calculations with the published results. In this work, the electronic transitions are assigned (in terms of both symmetry and allowedness, now in a spectroscopic sense) and a discussion is presented regarding “the change in molecular bonding upon excitation is consistent with the observed primary photochemical processes: dissociation to carbenes and N_2_ in diazoalkanes, similar dissociation or rearrangement to diazomethanes in the diazirines”.^155^

**Figure 36:**
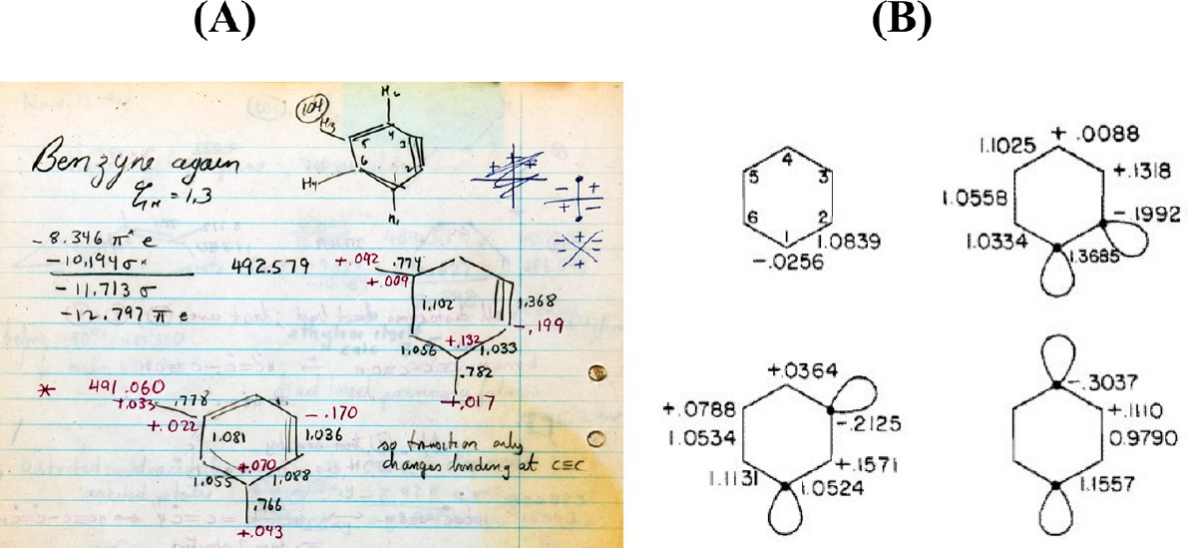
(A) Page 104 from Roald Hoffmann’s *Summer 1964 → Nov 1964* laboratory notebook^157^ with (B) his subsequent publication^40^ in which he reported the “Mulliken overlap populations (unsigned numbers) and charges for the benzynes.” Compare (A) with the *o*-benzyne in the upper right-hand corner in (B).

As a last computational example, Hoffmann and his postdoctoral student Akira Imamura and Cornell undergraduate Warren J. Hehre submitted their paper^156^ on benzynes on May 31, 1967, two years after Hoffmann had joined Cornell University. This paper is based in part on extended Hückel calculations peformed by Hoffmann approximately November 12, 1964—in the midst of his writing *Stereochemistry of Electrocyclic Reactions* and approximately 2 weeks from its submission date. Yet it was published in 1968. Figure 36 illustrates Hoffmann’s use of many of his calculational results, even several years after their generation. Next to an excerpt from his 1964 notebook page is an excerpt from the 1968 paper. In this study, Hoffmann et al. examine through through-bond and through-space “specific interactions among radical lobes in the same molecule separated by a number of intervening σ bonds.”^156^ “The interaction is shown to depend only on the orientation of the σ bonds between the radical lobes and the orientation of the lobes themselves, not on the specific molecule.”

During this time period, Hoffmann was both learning organic chemistry^158^ and deeply thinking about organic chemistry—even if he was not deeply thinking about *The Woodward Challenge*, at least until the fall of 1964. Within the notebook are his thoughts and impressions of various aspects of chemistry. One example is provided herein.

During the summer of 1964, while in Sweden, Hoffmann was learning a lot of organic photochemistry and applying extended Hückel theory to the excited states of many molecules, including ketones. As shown in the graphics and logic outlined in two pages of his notebooks (SI-1 and SI-2 where SI refers to the Supporting Information), Hoffmann was studying the relative rates of bond cleavage in photochemical reactions. He drew several energy diagrams and concluded,

“But my argument is not that the weakest bond breaks first in the excited state. But the bond which is weakened most.”^159^

This section about the other research interests of Woodward and Hoffmann during those seven months of 1964 closes with Roald Hoffmann’s insight about the holistic nature of scientific research and the Woodward–Hoffmann rules.

“Everything is connected. Ketones make me focus on photochemistry. I wouldn’t have known as much photochemistry if I had not worked on the ketones. The classical and nonclassical carbonium ions prepared me for the cyclopropyl solvolysis. I was lucky, to have interacted with Corey before this, and later, Applequist’s course came at the right time. I go to lectures, I write to [Paul] Schleyer and [Jerry] Berson and others.”^160^

## XI Observations and Conclusions

Beyond the observations and conclusions discussed throughout this paper, there are a number that deserve attention. These fit into ten categories.

### A Nature of Scientific Discovery

“*What does this story tell us about the nature of discovery? Is it a simple eureka moment, or slow, scrabbling for understanding? What does the story tells us about the importance of formulating the problem to getting an answer to it?”* In this paper, the timing begins at the May 5, 1964, meeting between Woodward and Hoffmann. Woodward comes prepared with an answer, perhaps only a hypothesis, perhaps even only a hint of an idea. Much comes before May 5, 1964, only a portion of which has been discussed to date,^51,54,91,149^ and more will be said regarding events prior to May 5, 1964, by this author in a future publication and perhaps by others in the future as well.

That being said, what was discovered between May 5 and November 30, 1964?

Chemists learned that the stereochemistry of electrocyclic reactions was governed by electrons in specific orbitals and that molecular orbital theory could account for that stereochemistry and predict new chemistry. Chemists, especially experimentalists, perhaps most especially organic chemists, learned of the great synergy that is possible in the melding of theory and experiment.

Woodward, the greatest experimentalist of the era, learned that MO theory could be of direct use in organic chemistry. In a way, Woodward knew that from his earlier encounters with MO theory in the electronic structure of ferrocene^162,163^ and the MO explanation of the octant rule.^164^ But now MO theory was closer to home, so to speak, and able to explain stereospecificity in organic reactions, a Woodward hallmark and one slice of Woodward’s mysterious reactions. Woodward also learned that Hoffmann was not just another calculator, but that he was seriously interested in organic chemistry and capable of making important, independent contributions.

Hoffmann learned that there was much more to the application of extended Hückel calculations than he had imagined—even though it was he who primarily invented the method. He learned that there was much more than total energies, energies of specific MOs, and bond orders; for example, the importance of potential energy surfaces and the need to model the transition state, not just the starting materials and products in a reaction. He learned the power and simplicity of the frontier orbital method. His calculations supported Woodward’s qualitative HOMO Frontier Orbital explanations. But by the end of November 1964, Hoffmann had not yet seen the power of correlation diagrams for organic reactions and had not yet seen the relationship of cycloadditions and sigmatropic reactions with electrocyclizations. Perhaps Hoffmann was too focused on using extended Hückel for a vast variety of organic compounds—on grabbing all those sea shells that lay visible on the beach rather than seeing the diamonds hidden below the surface. Hoffmann was not yet ready to expand his vision into explaining the reactivity of other concerted reactions by the orbital symmetry concepts proposed in *Stereochemistry of Electrocyclic Reactions*.

### B Role of the Scientific Community

“*What was the importance to this story of interactions in the community, literature, seminars, courses, and international meetings?*”

As he was pondering in 1963 whether he would accept an academic position or the Junior Fellowship, Hoffmann was beginning to understand that he uniquely had a tool, the extended Hückel method, which could be applied to all of organic chemistry. He would begin with alkanes and then move deeper and deeper into this science. Whatever interesting chemistry Hoffmann would hear from the community—in his discussions with fellow students and with Harvard’s faculty, especially with E. J. Corey; with the literature which he was absorbing, and not just the literature of physical chemistry and chemical physics; in courses that he would sit in, as an observer and learner, not as an educational requirement; and at scientific meetings—all those ultimately did more than teach. Those meetings introduced Hoffmann to his future colleagues in the (mostly) academic community; the meetings also exposed him to what was leading edge research and leading edge problems and techniques. And at least one meeting introduced him to an extension of the two-electron electrocyclization and the distinction between which of two allowed reactions would predominate (thanks to DePuy). As discussed above, the meetings propelled him to finally document his many months’ old research results into his portion of *Stereochemistry of Electrocyclic Reactions*.

Douglas Applequist’s course came at just the right time for Hoffmann. It was specific, it was physical in its orientation, and it was interesting. And it was small ring organic chemistry, perfect for calculations (a relatively low number of atoms) and germane to *The Woodward Challenge*.

The literature is another facet of the role of the scientific community. Hoffmann laboratory notebooks are witness to how quickly Hoffmann took to the chemical literature—through pages and pages of readings—and how devoted he was to the literature. Would he have done better in the age of SciFinder? Hoffmann doubts it.^165^ And we have seen how Woodward’s command of the literature, his singling out of crucial problems, played an important role in the orbital symmetry story.

### C Role of Institutions

*“What is the importance of institutions (to this story, the Society of Fellows)?*”

Certainly, Hoffmann’s being a Junior Fellow of the Harvard Society of Fellows provided both the those resources and the encouragement—indeed, the direction and opportunity—to carry out independent research without either financial imperatives or teaching obligations. But that freedom, resources, and encouragement are the essence of an academic career, especially at a major research university. As Arnold Beckman said, “Limited only by one’s own imagination.”^166^ Perhaps one should add motivation to imagination.

What distinguished a Junior Fellowship from other postdoctoral positions is that Hoffmann could choose his collaborators. Hoffmann was “lucky”^85^ in his choice. And so was Woodward.

Harvard was the institution that brought Woodward and Hoffmann together and held them in a position such that they would eventually interact.

The institutions of science, from the Junior Fellowship at Harvard to the seminars and conferences he attended (though not as an invited speaker!), served him well. Indeed, as proposed above, these public modes of communication within the scientific community likely propelled the writing of *Stereochemistry of Electrocyclic Reactions* in November 1964.

### D Contributions to Chemistry

The appearance of *Stereochemistry of Electrocyclic Reactions* and the subsequent Woodward–Hoffmann papers explained experimental results that had been literature mysteries for years. That fact notwithstanding, the possible explanation had been suggested by Oosterhoff (Leiden) and published in a paper by Havinga and Jos Schlatmann in *Tetrahedron* in January 1961.^92,167^ We will learn more of Oosterhoff—and of Fukui, who also came very close to proposing what was later termed “Conservation of Orbital Symmetry”—in a subsequent paper.

Of particular note is that not a single new experimental result is included in *Stereochemistry of Electrocyclic Reactions*. Explanatory papers, that is, those which interpret or reinterpret the results of others, were becoming rarer and rarer among the pages of leading chemistry journals of the time. From a historical perspective, Alan Rocke pointed out some years ago that August Kekule’s theory of aromatic compounds (1866) was also devoid of new experimental results.^168^ But Kekulé exercised caution, according to Rocke, seeking “safety in obscure and hesitant language”. In contrast, Woodward and Hoffmann were unambiguously clear and direct though concise, even if their word count was more than standard for a *JACS* communication (see Figure 1).

The conservation of orbital symmetry concepts also provided the impetus for many research projects performed around the world and served as the basis for numerous natural products syntheses, as well. Even today, research continues on the finer points of the stereochemistry and reactivity of concerted reactions using the most advanced theoretical and experimental methods.^169^

Woodward and Hoffmann created a new mechanistic language with their several original words that immediately joined the lexicon of organic chemists.^170^ More new terms were to follow in subsequent W–H papers.

Several of Hoffmann’s calculations were among the earliest examples of reaction potential energy surfaces for organic reactions, although even today, they are not always recognized as such. For example, in his recent autobiographical perspective, Charles Perrin wrote, “Hoffmann developed his theory initially by calculating the molecular orbitals of the reactant, rather than the energetics of the transition state.”^171^ As discussed herein, this is a mischaracterization of Hoffmann’s extended Hückel calculations performed on *The Woodward Challenge*. Hoffmann modeled motion on an extended Hückel energy surface of several electrocyclic reactions, e.g., the ring opening of cyclopropyl-X to allyl carbocation, ring closure of 1,3-butadiene to cyclobutene and the reverse, and ring opening of 1,3-cyclohexadiene to 1,3,5-hexatriene (but not its reverse). Given the then computational complexity of these molecules (!) and the limited power of computers in 1964, only several points on the reactions surface were examined. These can be seen in Figures 12–17.

One essential factor in the acceptance of theories, as discussed by Hoffmann (in *Why Buy that Theory* published in 2003 in *American Scientist*(172)) is that theories not only rationalize, but make predictions, preferably risky ones. And that “the predictions can be tested in a graduate student’s lifetime, the unit of investable labor in the American system”.^165^ More predictions were to come from Woodward and Hoffmann for cycloadditions^152,153^ and sigmatropic reactions,^150,151^ but even in the constrained subject matter of *Stereochemistry of Electrocyclic Reactions*, the theory was making ample predictions easily verifiable. For instance, predictions were made for charged species with five and seven carbons and neutral ones for eight.^1^ One can see some of these tested in the interval between 1965 (this paper) and the full *Angewandte Chemie* paper of 1969.^124^

### E The Role of Ambition

*Were Woodward and Hoffmann driven by the lure of fame?* Certainly not for their first publication. Woodward clearly recognized the importance of the problem; he had been struggling to solve the four mysterious reactions for at least four years. He surely was aware that others knew of one or more of these mysterious no-mechanism reactions and were likely thinking deeply about the problem. But the evidence discussed above—that Woodward did not pressure Hoffmann to complete his work—strongly suggests that Woodward’s eye was not on the competition nor was he gunning for more “notches in his pistol”.

As for Hoffmann, he openly acknowledges that in the first months of their collaboration he failed to estimate correctly the importance of the problem. But Hoffmann also points out that he has never been motivated by ambition, only by “the joy of understanding, the joy of making sense of chemical complexity”.^173^ Shall we believe him? There is little evidence to the contrary in the story of the first orbital symmetry paper. This is a theme that will be examined further in a subsequent publication.

### F Effect on Woodward and Hoffmann as Individual Scientists

While this and the subsequent W–H papers marked a conceptual breakthrough in organic chemistry, the impact on both Woodward’s and Hoffmann’s lives was also particularly noteworthy. Woodward would receive his Nobel Prize in the very year that the five Woodward–Hoffmann communications appeared—for his research in natural products synthesis. There is no doubt that the orbital symmetry work—the five *JACS* communications, all published in 1965—played no role in the decision by the Swedish Royal Academy of Sciences to award the 1965 Nobel Prize in chemistry to Woodward. But that such a breakthrough contribution requiring the application of orthogonal disciplines would occur in the same year as his Nobel Prize would cement Woodward’s celestial reputation forever. Perhaps there was never such a spectacular banner year for any other scientist as was 1965 for R. B. Woodward.

For Hoffmann, he was at the right place at the right time with the right tool, with the right ambition and energy, and with the right set of interests. In 1996, Hoffmann summarized the first 40 years of his career,

“In existing as a scientist, meaning that my work was of continuing interest to other chemists, I was helped in that I moved into whatever part of chemistry I did, just a little ahead of the heavy guns of computational chemistry.”^5^

In June of 1965, Hoffmann left Harvard for Cornell—where he would stay for his entire professional career—just after the third of the five 1965 Woodward–Hoffmann *JACS* communications appeared. Hoffmann’s publication record, including but not overshadowed by the Woodward–Hoffmann publications, presented, as we now see it in retrospect, a marvelous package to bring to one’s first academic position. Of course, credit goes to Cornell, as their job offer was presented to Hoffmann—and accepted by him—prior to January 27, 1965 (see Figure 32), the approximate date *Stereochemistry of Electrocyclic Reactions* appeared in print, not after all five of the Woodward–Hoffmann 1965 communications had appeared. Certainly, the W–H publications solidified Hoffmann’s reputation in the multidisciplinary endeavor of the determination and explanation of structure–property—reactivity relationships, the field in which he continues to this day. But they did not get him his first academic position!

### G Nature of Collaborations

“*Regarding collaboration, between young and old researchers, among scientists from different disciplines; how does a helping hand turn into a real collaborator?*” It is not clear that Woodward provided a “helping hand” to Hoffmann in the period May 5 and November 30, 1964. He did offer Hoffmann the opportunity to solve a massively important and, to Hoffmann, a previously unknown scientific problem. By contrast, Woodward was actually asking for a helping hand from the much younger Hoffmann. Woodward was fortunate to have Hoffmann nearby so as to conveniently and easily invite a collaboration with Hoffmann. Furthermore, Woodward was wise to seek a collaboration with Hoffmann—only Hoffmann had the tools to study the problem computationally at the time. It was that mutuality between the young and the senior, the unknown and the massively worshipped (the David and the Goliath), the need of one for the complement of the other—not that there is any evidence Hoffmann was aware of that need—that formed a true collaboration.

In this evolution, eventual equality (if not a role reversal) would result. The fertilizer was a blending of interests, where the organic chemist became a theoretician and the theoretician became an organic chemist. This is an excellent example of where one field influences another, primarily as one collaborator influences the other (indeed both influenced each other). We will see this phenomenon continue, as the study of the Woodward–Hoffmann collaboration continues.

The Woodward–Hoffmann collaboration was one of the first examples of synergy between an experimental—in this instance, synthetic—chemist and a computational chemist and theorist. Indeed, the inclusion of computational results may have been Woodward’s strategy to overcome any criticism of a lack of new experimental data in this publication. It was not easy then, nor is now, to publish ideas, even good ones, in the absence of new experimental or computational data. The extended Hückel results provided by Hoffmann in the second half of the paper were complementary to the frontier orbital explanation, provided by Woodward in the first half of the paper.

### H On the Nature of the Woodward–Hoffmann Collaboration

The evidence does not bear out a *close* collaboration between R. B. Woodward and Roald Hoffmann on their first joint paper. Interactions between the organic chemist and the computational—theoretical—chemical physicist was limited to their brief meeting, recorded in Hoffmann’s laboratory notebook to have taken place on May 5, 1964, and their brief interactions during the cut-and-paste preparation of *Stereochemistry of Electrocyclic Reactions*. Had Woodward and Hoffmann met more frequently, and they may well have, those meetings are not remembered by Hoffmann and as such, were not particularly memorable. Given that in the 1960s and 1970s Woodward’s time with his students was very limited, likely each meeting with the Pope of Organic Chemistry^47,48^ would have made an impact on Hoffmann.

Woodward’s contribution was recognizing the chemical problem; abstracting it from a complex literature, new and old; bringing this to the attention of Hoffmann and inviting his participation; more than that, proposing a frontier orbital explanation based on phases and nodes of the relevant orbitals; writing the first half of the manuscript; assembling and melding the two halves together; and writing an especially self-assured cover letter to *JACS* (Figure 1).

Woodward showed enormous patience. (Surely Woodward was expecting *something* from Hoffmann after their meeting on May 5, 1964.) There is no evidence of his urging Hoffmann to complete his extended Hückel calculation. This suggests that Woodward was not concerned about competition. A letter (Figure 32) from Hoffmann to Jean-Marie Lehn in early 1965 hints—or “states,” if we take Hoffmann’s words literally—that the delays in publication were due to Woodward.^138^

Could Woodward have been unaware of the magnitude of the importance of the project? Unlikely. First, we have in Woodward’s own words his four mysterious reactions. Second, we have Ranganathan’s stereochemical results in the vitamin B_12_ synthesis, opposite to that predicted by Woodward. These unanticipated results, in his own laboratory, must have pained Woodward enormously.^51^ Certainly Woodward had a clear understanding about the worldwide interest in the problem, an understanding shared at that time by Doering,^9,10^ Corey,^54,55^ among others.

Thus, Woodward’s patience remains a mystery. It certainly suggests no concern about Corey’s claim, assuming that Corey’s perception^54,55^ of the events of May 4, 1964, was similarly perceived by Woodward.

Hoffmann’s contributions were to perform calculations on ring opening of cyclopropyl-X, 2,3-dimethylcyclopropyl-X, cyclobutene, and 1,3-cyclohexadiene and ring closure of 1,3-butadiene. The results were recorded in 11 pages of Hoffmann’s laboratory notebook. Approximately 180 pages of his laboratory notebooks in the time period in question deal with other chemistry. Hoffmann brought the rigor of theoretical fundamentalism to Woodward’s qualitative reasoning. That being said, the extended Hückel theory was not widely accepted by theoreticians as being robust and authoritative.

### I Role of Writing a Manuscript

*“Much thinking and discovery often takes place in the writing of papers, where words have to be put down, words that will convince a remote reader. Does that happen in this story?”* In the writing of *Stereochemistry of Electrocyclic Reactions*, the record suggests little substantive evolution from draft to draft and little cross fertilization between Woodward’s qualitative approach and Hoffmann’s quantitative results. They are cobbled together.

In contrast, Woodward and Hoffmann’s publication entitled *Conservation of Orbital Symmetry*, first published in *Angewandte Chemie*(124) in 1969 and then in book form^174^ in 1971, is much more than a review of their eight previously published papers. Furthermore, that publication goes well beyond discussing the experimental verifications published around the world following their five 1965 communications which contained stereochemical predictions for concerted reactions. Writing that “long paper”, as Hoffmann refers to *Conservation of Orbital Symmetry*, provided both the incentive and the opportunity to expand and document the broad reach of their concepts.

The author adds another comment regarding writing a manuscript, namely writing this manuscript. Hoffmann reflects,

“The notebooks enabled it; not only did I not realize I was doing good science [in 1964], I also did not realize that recording it would be pretty unusual.”^134^

And historically important.

### J For the History of Science

The availability of Hoffmann’s laboratory notebooks, and the fact that all the new research data in *Stereochemistry of Electrocyclic Reactions* were Hoffmann’s extended Hückel calculations, provide a nearly unique opportunity to follow the day-by-day research progress that led to a Nobel Prize in chemistry. It is remarkable that with the treasure trove of material in the Woodward archives at Harvard, there is so little datable material on the process leading up to the discovery.

And it is sad that Corey has chosen to not make available the material he has relevant to this discovery. Having primary source “data” is critical for historians to focus their ideas on facts rather than intuition and thus avoid fallacies of factual verification and significance in their studies.^175^ Of course, this requires that the papers of scientists be retained and then be archived and be accessible for scholarship.^176,177^

## XII Coda

An insightful and knowledgeable reviewer of this paper asked,

“One of the stories being told here is how researchers’ habits in creating and dating records (and their own and their intellectual executors’ care in preserving them) influence what gets written as history. The present Perspective serves as a textbook example of this by contrasting all that is known (not without gaps) about the evolution of Hoffmann’s thinking with the relative paucity of information about the evolution of Woodward’s. Is there anything about the historiographic problem that’s worth commenting on in this summary section?”

Woodward’s archives at Harvard contain many bulging files on the *Conservation of Orbital Symmetry*. Included among these papers are several hundred pages of Woodward’s chemical pictographs, often written on his favorite paper for that purpose: yellow note paper of uncertain but not archival quality, now long faded and quite fragile. Even a cursory skimming through the pages of this paper reveals not a single figure containing notes of Woodward during the period between May 5, 1964, when Hoffmann was invited by Woodward to collaborate on *The Woodward Challenge*, and the writing of their first publication. In part, that is because Woodward’s chemical pictographs are undated—as are most of Hoffmann’s laboratory notebooks and his notes of Applequist’s class on small ring chemistry. But at least Hoffmann’s notebooks and class notes are bound together, even if loosely, and there is some sporadic dating!

Woodward is a ghost in most of this story, hovering like the genius he was and constantly though silently present. It is Hoffmann who is the main character, and the story as presented herein is somewhat biased by having Hoffmann’s laboratory notebooks as the main source of information. Perhaps Woodward said, in his May 5, 1964 meeting with Hoffmann, all he wanted and needed to contribute to *Stereochemistry of Electrocyclic Reactions* until the time came for writing the paper. Even Woodward’s challenge is documented in Hoffmann’s hand (Figure 4). Due to the scant presence of dates in Hoffmann’s notebooks, that meeting is chronologically uncertain, because of both Corey’s assertion that the May 5, 1964, date is in error and Hoffmann’s representations in the early 1980s that he began research on the no-mechanism problem *before* his substantive meeting with Woodward. And Hoffmann cannot confirm that the May 5, 1964 meeting actually occurred, as he does not remember it! Other than the evidence in Hoffmann’s laboratory notebook (Figure 4), one might even ask: Did Woodward and Hoffmann even meet in early May 1964? Without some documentation and some primary sources of reliable information, all we have is speculation, Hoffmann’s uncertain memory, and Corey’s representations without substantiation.

We cannot know whether there is more to know of this time period and of Woodward’s intellectual growth therein or not. As the reviewer said, we know much of Hoffmann’s intellectual and “experimental” journey from May to November 1964, but not of Woodward’s. How often do scientists—or anyone in any field—keep rigorous and thorough notes for the benefit of history? Perhaps additional information will become available with time. I have much more to say about Woodward and Hoffmann pre-May 5, 1964, and about Woodward and Hoffmann post-November 1964. But these stories are for future publications.

## XIII Coda to a Coda

In his 1973 Cope Award address, Woodward basked in the spotlight and sunshine of his extraordinary position in science (Figure 6). Those moments must surely have been as marvelous as his memory of the moments of joy in discovering—and naming—the expansiveness of the Rules of Conservation of Orbital Symmetry. To relive those marvelous, thrilling moments is “supererogatory,” superfluous, Woodward tells the audience. And then, he does just that!

“Neither Professor Hoffmann nor I any longer find it à propos to expound principles which have already, in a spectacularly short time, become an integral, indispensable and powerful part of the basic theoretical structure of organic chemistry. Let me not be misunderstood here: for my part, at least, I still find it most thrilling to relive those marvelous moments of discovery and personal enlightenment. But, to expound the details in public has become, at best, supererogatory [superfluous].”^51^ – R. B. Woodward

But from a study of the written record, we now know how isolated Woodward was during the first stages of the development of the Rules of Conservation of Orbital Symmetry, and how long it was, and how unnecessary the delays were, from the first moments of discovery to the publication of *Stereochemistry of Electrocyclic Reactions*.

In contrast, Hoffmann recently mused,

“I don’t have any pretensions that what I did was great, even if the orbital symmetry story outcome was important for the science. But it is a story of how science is really done. What you [Seeman] add to it, not just in this paper, but in papers to come, is an unusual examination of alternatives. (Who else could have done it, so carefully examined by you?) That’s so important. [The eminent sociologist of science Robert K.] Merton would have loved it. It is also a story of how I was changed—my transformation from calculator to explainer—by what we found, and by “growing into” organic chemistry, and by the responses of the community to the work.”^178^ – Roald Hoffmann

In my many discussions with Hoffmann—often, really question and answer sessions that might resemble depositions to some onlookers—I have searched for constancy in behavior and in pattern by him and by Woodward and others involved in the development of the Woodward–Hoffmann rules. Hoffmann urges me to be less like an attorney. He says to me,

“Life is messy. Science in not all straight logic. And all scientists are not always logical. We’re just scrabblers for knowledge and understanding.”^68^

## XIV Source Materials

The information contained in this report is based on documents from the R. B. Woodward papers at the Harvard University Archives, the Roald Hoffmann papers at the Cornell University Archives, and in particular, within the papers of Roald Hoffmann at Cornell. Documents were also obtained from the Jerome A. Berson, Paul D. Bartlettt, and John D. Roberts papers at the Chemical Heritage Foundation Archives. The author has conducted multiday interviews with Hoffmann at the University of Richmond (February 9, 2008, and September 16, 2009) and at Cornell University (May 19, 2010, April 4–5, 2012, and May 18–21, 2015) and has conducted e-mail-based interviews with Hoffmann for nearly a decade. Furthermore, Hoffmann made his entire office files open to the author’s review and reproduction, without restriction. Indeed, Hoffmann provided copying assistance on several occasions. Figure SI-3 is the “map” of his Cornell University office that Hoffmann drew and gave to this author in 2012 to assist in the identification and location of papers related to the orbital symmetry story.^179^ Hoffmann also placed yellow Post-it Notes on the draws of the file cabinets to assist in the location of files. The author spent many days alone in Hoffmann’s office reading and copying these files when Hoffmann was out of town. These interviews and documents made it possible to examine the intimate details during the conduct of this research—including the kinds of information not generally available even in these days of data sharing and open access.^180−182^

## Supporting Information Available

Pages 36 and 37, respectively, of Roald Hoffmann’s *Summer → Nov 1964* laboratory notebook, where he qualitatively considers the relative rates of bond cleavage in photochemical reactions. Map^179^ drawn by Roald Hoffmann of his Cornell University office and given to J. I. Seeman in April 2012 for his use to locate, identify, examine and copy documents when Hoffmann was out of town(PDF)

## Dedication

Dedicated to those synthetic and physical organic chemists who, from the 1950s to the early 1960s, provided experimental data that formed the basis of the “no-mechanism” challenge and to my friend William von Eggers Doering (1917–2011) who coined this term and was so very close to solving it himself.
