# Supplementary material for: Woodward–Hoffmann’s Stereochemistry of Electrocyclic Reactions: From Day 1 to the JACS Receipt Date (May 5, 1964 to November 30, 1964)
Source: J Org Chem. 2015 Oct 27;80(23):11632–71. doi: 10.1021/acs.joc.5b01792 (PMC11393748; doi:10.1021/acs.joc.5b01792)
Supplement: Supplementary file 1 — jo5b01792_si_001.pdf [file jo5b01792_si_001.pdf]

SUPPORTING INFORMATION

Woodward-Hoffmann's *Stereochemistry of Electrocyclic Reactions*: From Day 1 to the *JACS* Receipt Date (May 5, 1964 to November 30, 1964)

Jeffrey I. Seeman*

Department of Chemistry, University of Richmond, Richmond, Virginia 23173, United States

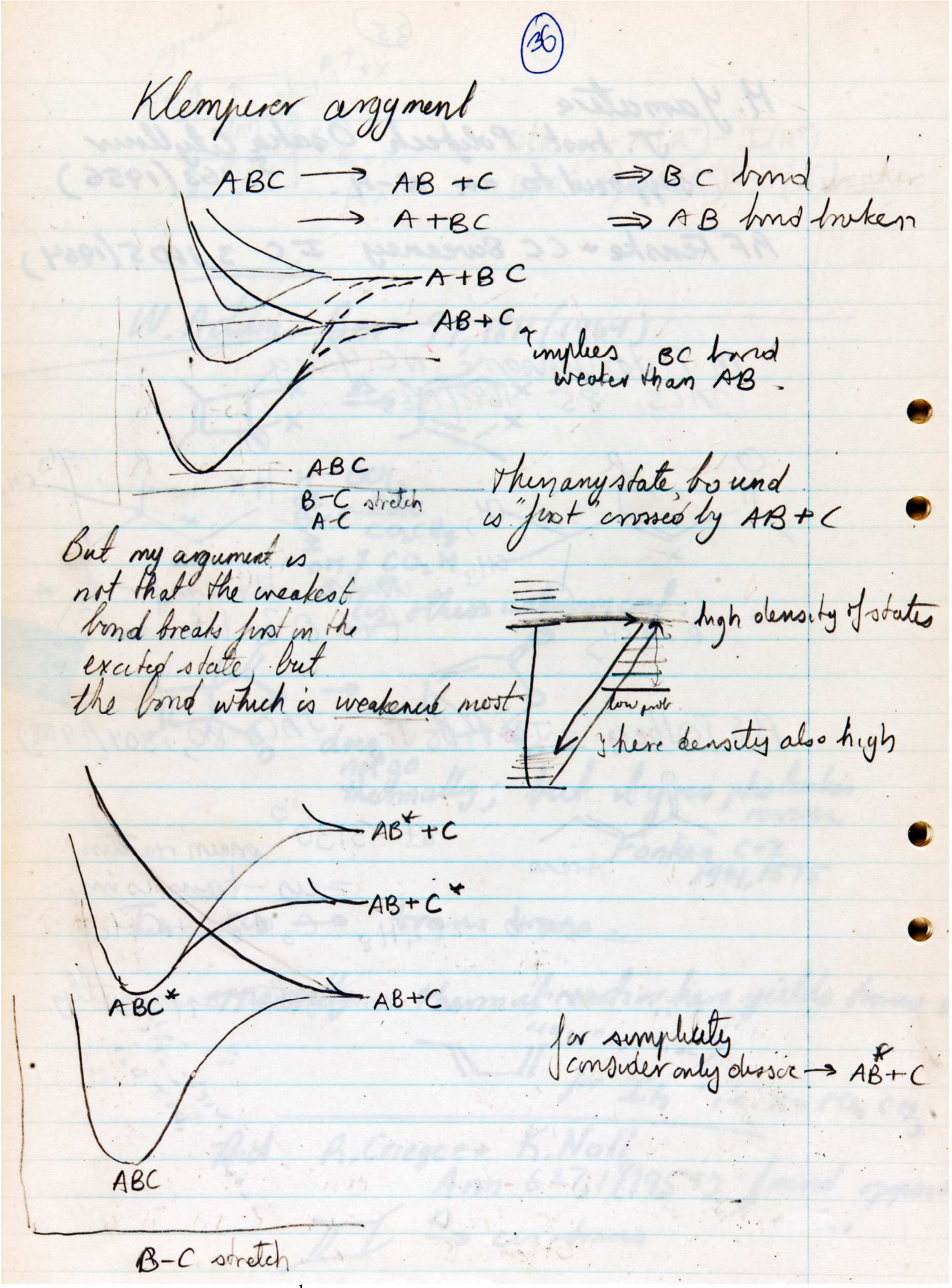

Figure SI-1. Page 36[1] of Hoffmann's laboratory notebook *Summer → Nov 1964* in which, together with page 37 (see Figure SI-2), he qualitatively considers the relative rates of bond cleavage in photochemical reactions.

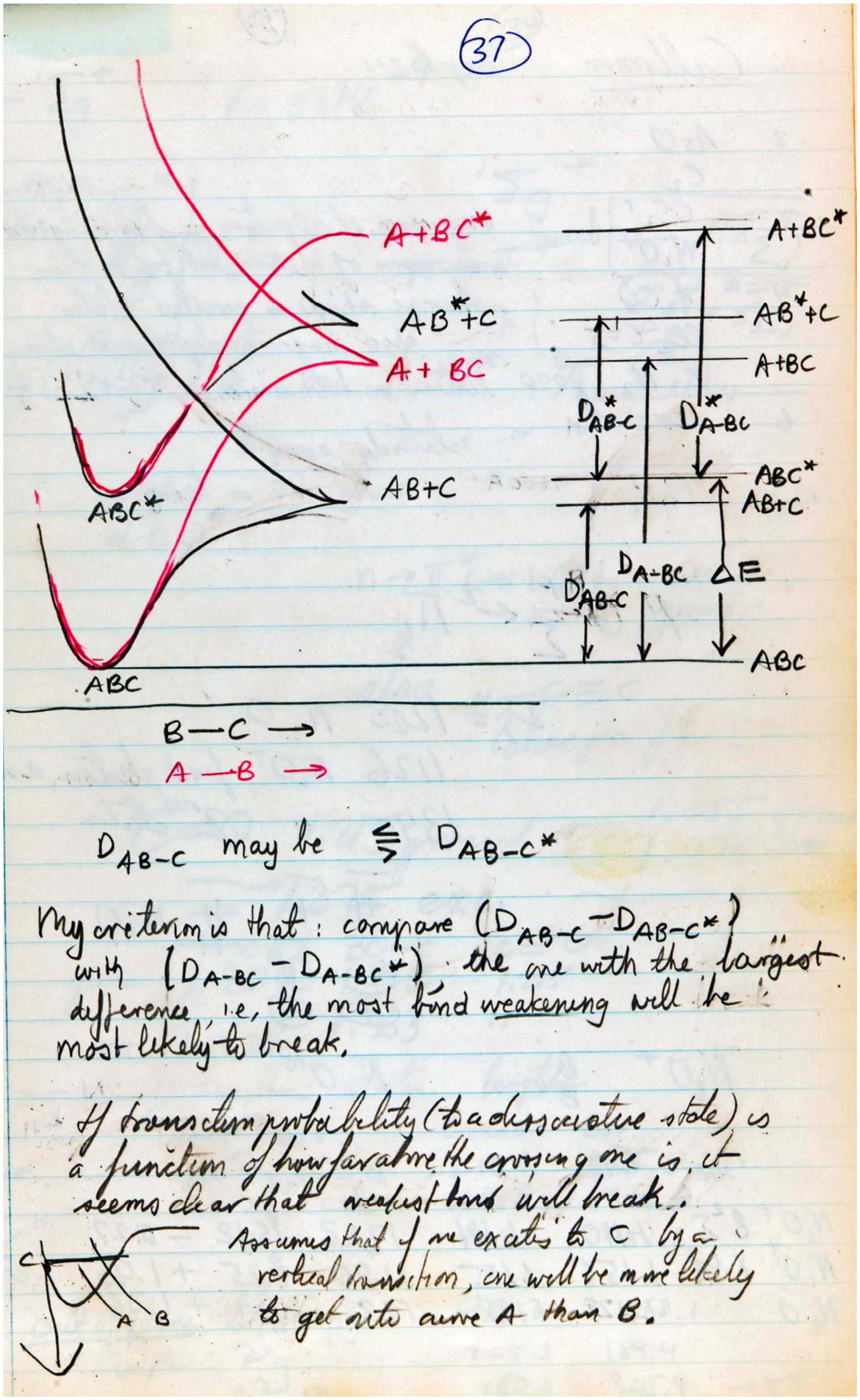

Figure SI-2. Page 37[2] of Hoffmann's laboratory notebook *Summer → Nov 1964* in which, together with page 36 (see Figure SI-1), he qualitatively considers the relative rates of bond cleavage in photochemical reactions.

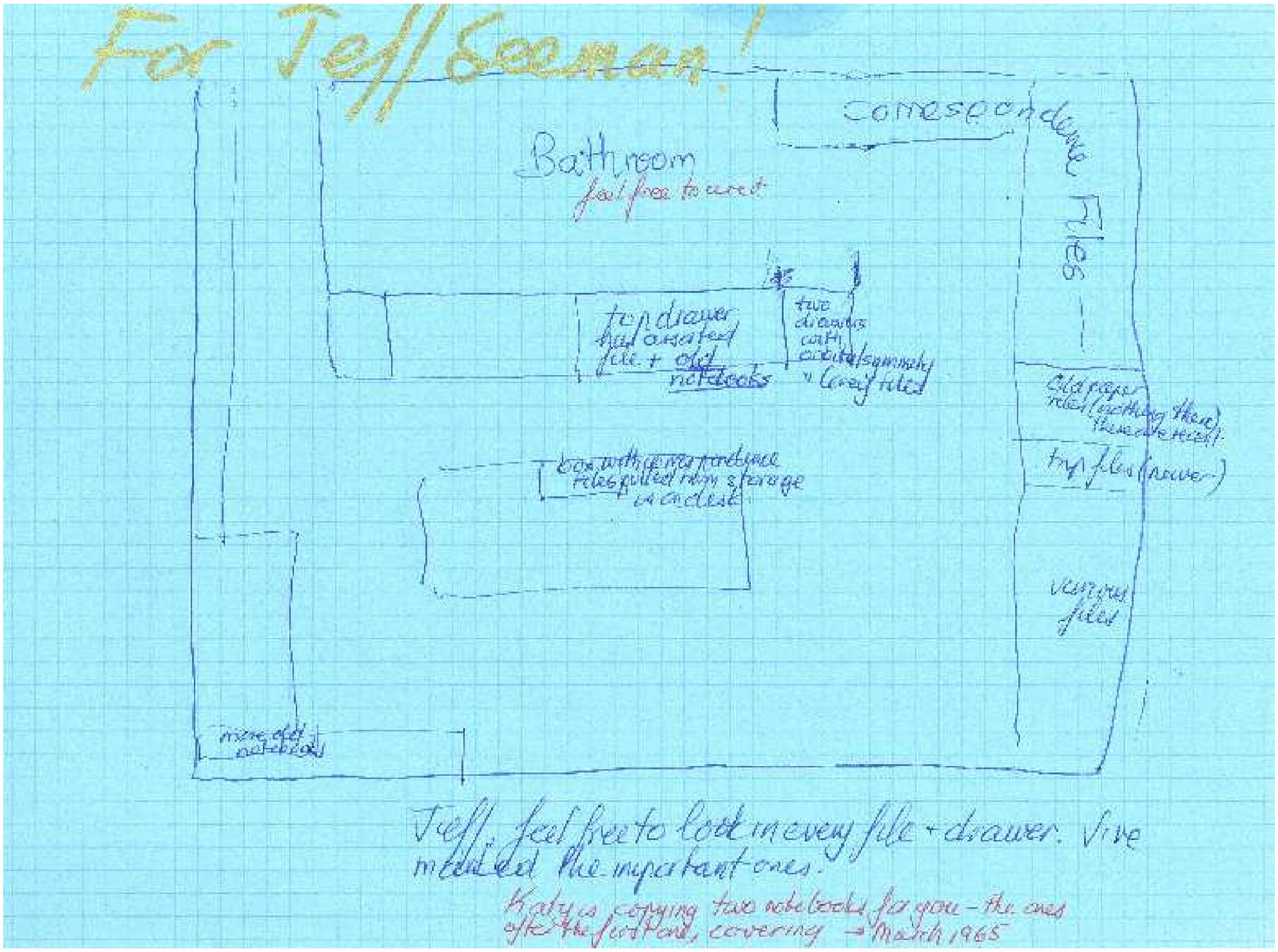

Figure SI-3. Map[3] drawn by Roald Hoffmann of his Cornell University office and given to J. I. Seeman in March 2012 for his use to locate, identify, examine and copy documents when Hoffmann was out of town. Clockwise from top, in gold ink, "For Jeff Seeman." In blue ink, "Bathroom, feel free to use it, correspondence files, old paper files, top files (newer), various files, more old notebooks, top drawer has assorted files + old notebooks, two drawers with orbital symmetry & Corey files, box with correspondence files pulled from storage is on desk." At the bottom, "Jeff, feel free to look in every file + drawer. I've marked the important ones." In red ink, "Katy is copying two notebooks for you – the ones after the first one, covering → March 1965."

(1) Hoffmann, R. *Laboratory Notebook (Summer --> November 1964)* Cambridge, MA, **1964**; p 36.

(2) Hoffmann, R. *Laboratory Notebook (Summer --> November 1964)* Cambridge, MA, **1964**; p 37.

(3) Hoffmann, R., Drawing of office for Seeman, J. I., March 29, 2012.
